# Synthesis and Antimicrobial Evaluation of (+)-Neoisopulegol-Based Amino and Thiol Adducts

**DOI:** 10.3390/ijms26104791

**Published:** 2025-05-16

**Authors:** Reem Moustafa, Attila Márió Remete, Zsolt Szakonyi, Nikoletta Szemerédi, Gabriella Spengler, Tam Minh Le

**Affiliations:** 1 Institute of Pharmaceutical Chemistry, University of Szeged, Eötvös u. 6, H-6720 Szeged, Hungaryszakonyi.zsolt@szte.hu (Z.S.); 2 Department of Medical Microbiology, Albert Szent-Györgyi Health Center and Albert Szent-Györgyi Medical School, University of Szeged, Semmelweis u. 6, H-6725 Szeged, Hungaryspengler.gabriella@med.u-szeged.hu (G.S.); 3 HUN-REN–SZTE Stereochemistry Research Group, University of Szeged, Eötvös u. 6, H-6720 Szeged, Hungary

**Keywords:** (+)-neoisopulegol, *α*-methylene-*γ*-butyrolactone, amino, thiol, antimicrobial

## Abstract

A library of neoisopulegol-based amino and thiol adducts was developed from (+)-neoisopulegol, derived from commercially available (−)-isopulegol. Michael addition of different nucleophiles towards its highly active *α*,*β*-unsaturated *γ*-lactone motif was accomplished, resulting in diverse amino and thiol analogs in stereoselective reactions. Then, the lactone ring was opened, with NH_3_ and benzylamine furnishing primary amide and *N*-benzyl-substituted amide derivatives, respectively. The in vitro antimicrobial effect of prepared compounds was also explored. The results revealed that naphthylmethyl-substituted *β*-aminolactone, the most promising compound, displayed selective inhibition for the Gram-positive bacteria *S. aureus* with an MIC (minimum inhibitory concentration) value of 12.5 μM. A docking study was performed to interpret the obtained results.

## 1. Introduction

The druglike chemical space contains an immense number of small molecules (>10^60^ possibilities); therefore, finding bioactive compounds among these small molecules is challenging. A possible solution to this problem is biology-oriented synthesis (BIOS). BIOS is based on the realization that 3D structures of protein domains as well as scaffolds of natural products (which evolved to interact with these proteins) are highly conserved in biology. Hence, it is reasonable to assume that analogs of bioactive natural products are more likely to inhabit a biologically relevant chemical space than other compounds. Indeed, compound libraries created according to BIOS principles show increased hit rates in biochemical and biological screens compared to traditional compound libraries [1,2].

**Figure 1:**
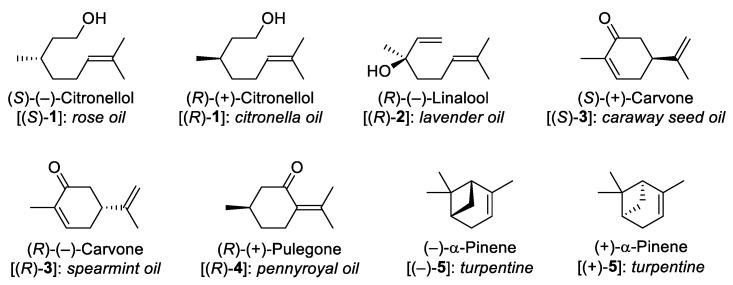
Examples of monoterpenes and monoterpenoids which are commonly used in chiral pool syntheses.

Monoterpenes and monoterpenoids (C_10_ compounds containing two isoprene units) are abundant in nature. They show great structural diversity (including various extents of functionalization), often contain a number of chiral centers, and many of them are available at affordable prices (importantly, in a number of cases, both enantiomers are accessible) [3,4]. Therefore, they are often employed as enantiopure starting compounds for the synthesis of various complex molecules, such as natural products [3,4,5], *β*-amino acids [6], etc. Some synthetically useful members of this compound family are depicted in Figure 1. It is also worth mentioning that monoterpenes and monoterpenoids often show biological activity [7], making them promising agents from the viewpoint of BIOS.

**Figure 2:**
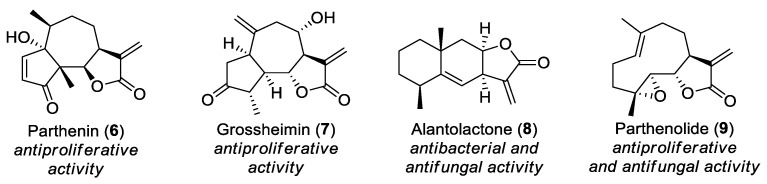
Examples of sesquiterpene lactones.

Sesquiterpene lactones are a large and diverse subclass of C_15_ terpenoids (Figure 2). Their characteristic structural element is a *γ*-lactone ring (in most cases, it is an *α*-methylene-*γ*-lactone motif). The main sources of sesquiterpene lactones are plants of the *Asteraceae* family, but they are also found in numerous other plants and in some fungi. Sesquiterpene lactones usually show cytotoxic, antitumor, or antibacterial activities [8,9,10].

**Figure 3:**
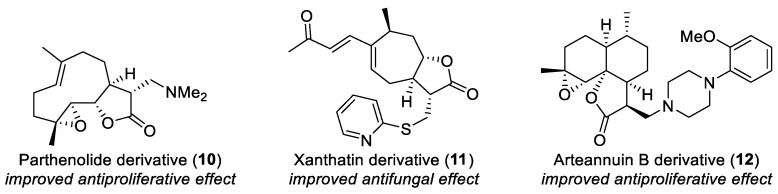
Bioactive Michael adducts of sesquiterpene lactones.

Many sesquiterpene lactones are reasonably accessible, and their *α*-methylene-*γ*-lactone motif can be transformed by a variety of ways. Therefore, from the viewpoint of BIOS, these terpenoids can serve as valuable starting compounds in the synthesis of compound libraries. Indeed, Michael adducts of sesquiterpene lactones have been widely studied, and diverse biological activities were reported (Figure 3) [11,12,13,14,15,16,17,18,19,20].

**Figure 4:**
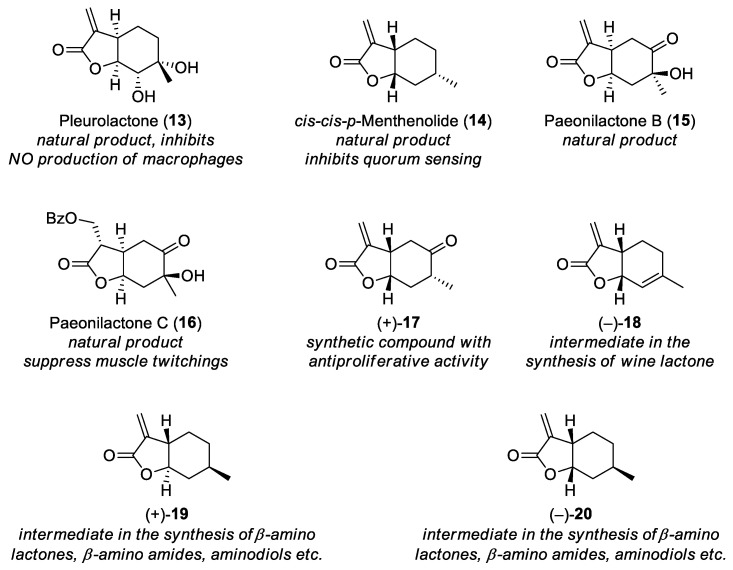
Monoterpene lactones and their relevant properties.

In contrast with sesquiterpene lactones, monoterpene lactones are rare in nature (Figure 4). Pleurolactone (**13**) was isolated from two edible *Pleurotus* mushrooms (*P. eryngii* and *P. cornucopiae*) [21,22], and shows moderate inhibitory activity against nitric oxide production in lipopolysaccaride-activated macrophages [22]. *cis,cis-p*-Menthenolide (**14**) is the second major component of the essential oil of *Mentha suaveolens ssp. insularis*, and it inhibits quorum sensing of *Chromobacterium violaceum* [23]. Paeonilactone B (**15**) was isolated from the roots of the plant *Paeonia albiflora* (also known as *P. lactiflora*), together with paeonilactone C (**16**). The latter compound can be considered the *O*-benzoylated *oxa*-Michael adduct of paeonilactone B and water, and both directly and indirectly suppresses stimulated muscle twitchings of frog sciatic nerve–sartorius muscle preparations [24].

Synthetic exomethylene lactones with a monoterpene skeleton are also known (Figure 4). Compound (+)-**17** possesses antiproliferative activity [25], while other compounds are useful intermediates in organic synthesis, e.g., lactone (−)-**18** can be transformed into the natural compound wine lactone [26,27] or its epimer [27]. Lactones (+)-**19** [5,28,29,30,31,32] and (−)-**20** [5,29,33] are especially versatile. These can be obtained in some steps [5,34,35,36,37] from readily available (−)-isopulegol (an intermediate in the synthesis of menthol [5]), and our research group utilized them extensively to prepare *β*-amino lactones [29,33], *β*-amino amides [29], aminodiols [29,33], and other products [5,29,30,31,32,33].

Based on our previous experiences with (+)-neoisopulegol-derived *α*-methylene-*γ*-lactone (−)-**20** and the importance of bioactive Michael adducts of sesquiterpene lactones, our aim was the synthesis of Michael adducts of compound (−)-**20** and investigation of the antimicrobial effect of the obtained products. A docking model was also developed for the most potent analogs.

## 2. Results and Discussion

### 2.1. Synthesis of (−)-α-Methylene-γ-Butyrolactone (−)-***20***

**Scheme 1:**
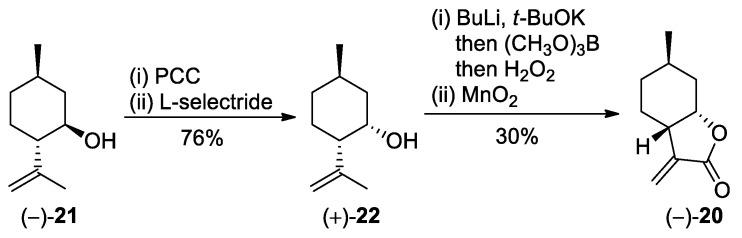
Preparation of (−)-*α*-methylene-*γ*-butyrolactone (−)-**20**.

(+)-Neoisopulegol **22** was synthesized in two steps from commercially available (−)-isopulegol **21** according to modified literature methods. First, oxidation of the hydroxy group of (−)-**21** with pyridinium chlorochromate (PCC) provided (−)-isopulegone [5,38], which, in turn, was diastereoselectively reduced with a stoichiometric amount of L-Selectride into the desired (+)-neoisopulegol (+)-**22** (Scheme 1) [5,36].

From (+)-neoisopulegol **22**, the key intermediate (−)-*α*-methylene-*γ*-butyrolactone (−)-**20** was prepared by modified literature methods. First, allylic borylation and subsequent treatment with H_2_O_2_ resulted in regioselective hydroxylation [5,37]. Then, treatment of the obtained diol with MnO_2_ led to the oxidation of the primary alcohol motif to the carboxylic acid group, followed by spontaneous ring closure to form lactone (−)-**20** (Scheme 1) [5,36].

### 2.2. Synthesis of (+)-Neoisopulegol-Based Amino Adducts

**Scheme 2:**
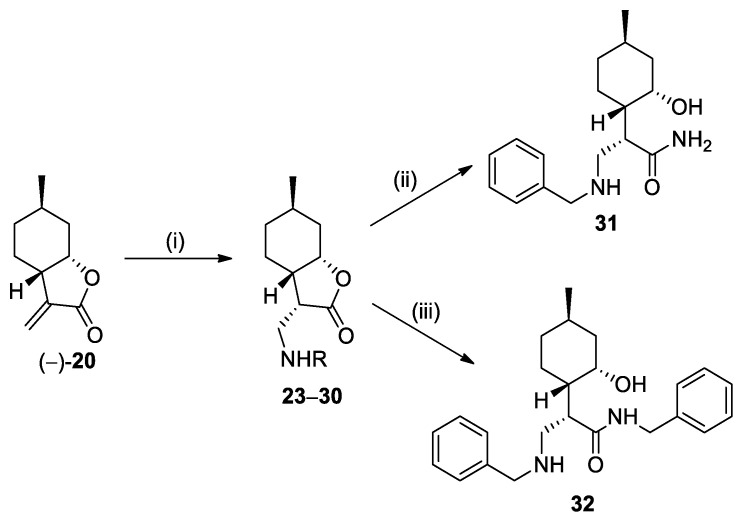
Preparation of (+)-neoisopulegol-based *β*-aminolactones and amides. Conditions: (**i**) RNH_2_ (1.5 equiv.), dry EtOH, 25 °C, 24 h, 42–90%; (**ii**) 25% NH_3_ in MeOH, 25 °C, 24 h, 94%; (**iii**) BnNH_2_ (2 equiv.), dry THF, 70 °C, 48 h, 70%.

Nucleophilic addition of benzylamines to (−)-*α*-methylene-*γ*-butyrolactone (−)-**20** provided (+)-neoisopulegol-based *β*-aminolactone **23** [29] and its analogs **24**–**30** with high diastereoselectivity (Scheme 1, Table 1). Previously, we observed that transformation of *β*-aminolactones into *β*-amino amides sometimes improves biological activity [29]. As a consequence, the ring opening of aminolactone **23** was performed with NH_3_/MeOH and benzylamine. The products were primary amide **31** and benzyl-substituted *β*-aminoamide **32** [29], respectively (Scheme 2).

### 2.3. Synthesis of (+)-Neoisopulegol-Based Thiol Adducts

**Scheme 3:**
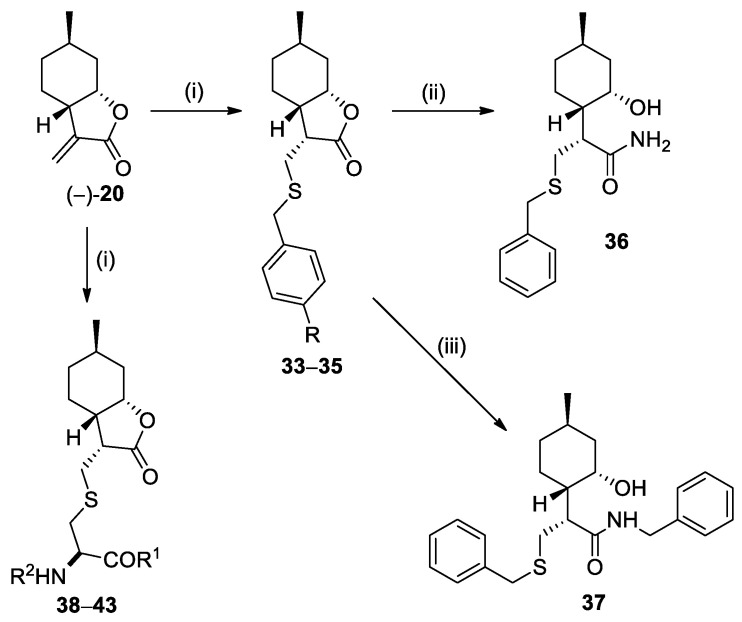
Preparation of (+)-neoisopulegol-based thiol adducts. Conditions: (**i**) RSH or cysteine derivatives (1.5 equiv.), Et_3_N (2.8 equiv.), EtOH, 25 °C, 24 h, 41–93%; (**ii**) 25% NH_3_ in MeOH, 25 °C, 24 h, 95%; (**iii**) BnNH_2_ (2 equiv.), dry THF, 70 °C, 48 h, 54%.

Besides amines, Michael addition of (−)-*α*-methylene-*γ*-butyrolactone (−)-**20** was also performed with various thiols. Unlike the conjugate addition of amines, formation of thiol adducts required Et_3_N as base [14,31]. Utilizing substituted benzyl mercaptans, the corresponding thiol adducts **33**–**35** were obtained (Scheme 3, Table 2). Nucleophilic addition was also successfully performed with cysteine derivatives as sulfur nucleophiles [39], yielding thiol adducts **38**–**43** (Scheme 3, Table 3). Similar to our previous work [31], ring opening of lactone **33** with NH_3_ and benzylamine was also performed, providing primary amide **36** and benzyl-substituted amide **37** (Scheme 3).

### 2.4. Synthesis of (+)-Neoisopulegol-Based Azole Adducts

**Scheme 4:**
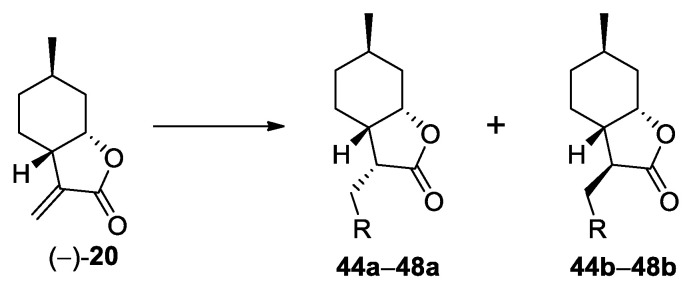
Preparation of (+)-neoisopulegol-based azole adducts. Conditions: azole (2 equiv.), DBU (1 equiv.), dry acetonitrile, 70 °C, 24 h, 12–57%.

A simple method to obtain the azole adducts of an *α*-methylene-*γ*-butyrolactone in the presence of 1,8-diazabicyclo[5.4.0]undec-7-ene (DBU) as a base was previously described in the literature [32,40]. When this procedure was applied to (−)-*α*-methylene-*γ*-butyrolactone (−)-**20**, the desired products were obtained in moderate yields (Scheme 4, Table 4). In contrast with the reactions depicted in Scheme 2 and Scheme 3, these reactions were not completely diastereoselective.

**Scheme 5:**
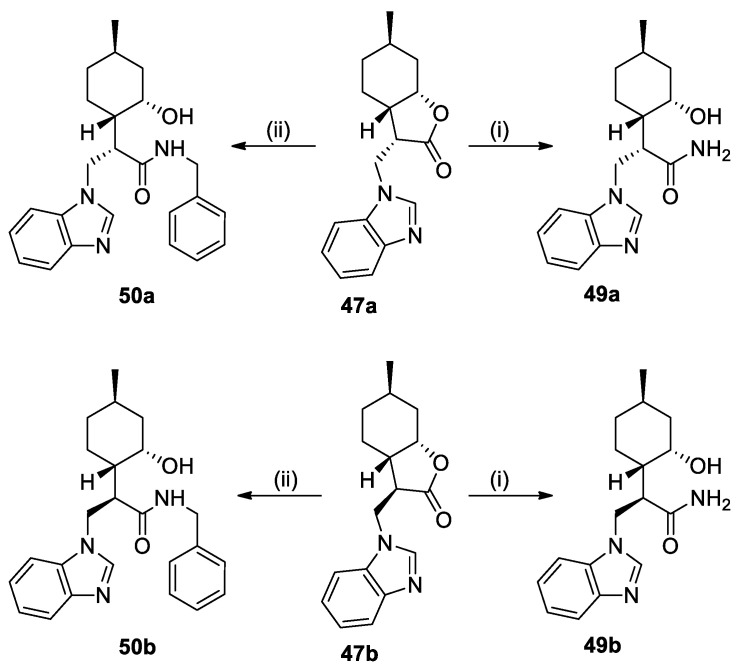
Lactone ring opening of azole adducts **47a** and **47b**. Conditions: (**i**) 25% NH_3_ in MeOH, 25 °C, 24 h, 59% (**49a**) or 69% (**49b**); (**ii**) BnNH_2_ (2 equiv.), dry THF, 70 °C, 72 h, 39% (**50a**) or 60% (**50b**).

Similar to our previous work [32], the ring opening of lactones **47a** and **47b** was successfully performed to obtain primary amides **49a** and **49b** and benzyl-substituted amides **50a** and **50b**, respectively (Scheme 5).

### 2.5. Determination of Relative Configuration of Synthesized (+)-Neoisopulegol-Based Amino and Thiol Adducts

**Figure 5:**
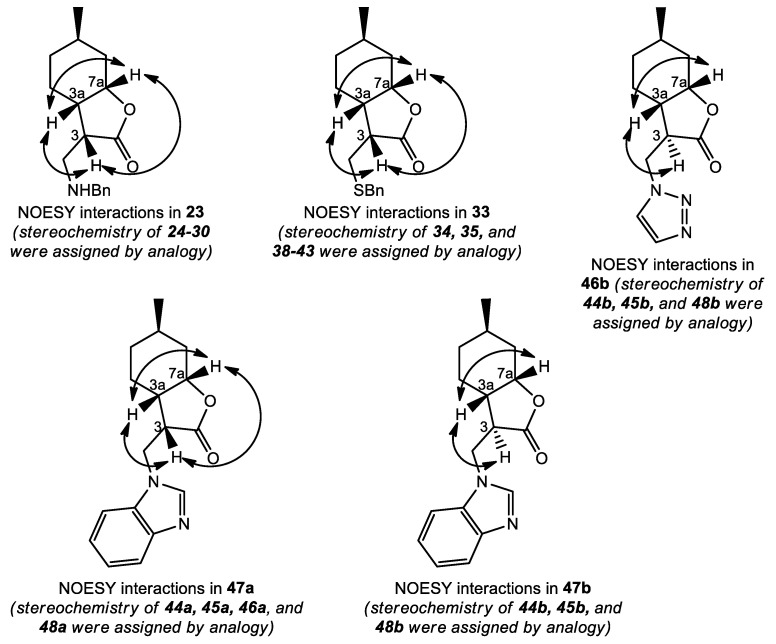
Determination of the relative configurations of (+)-neoisopulegol-based Michael adducts.

In order to determine the relative configuration of the synthesized (+)-neoisopulegol-based Michael adducts, from each compound family, some selected compounds were subjected to NOESY experiments. Once their stereochemistry was determined using NOE signals (or the lack of NOE signals) between H-3, H-3a, and H-7a, the configurations of related compounds were assigned by analogy. The results are summarized in Figure 5. Amino adducts, thiol adducts, and the major product azole adducts had the same stereochemistry at C-3, while the minor product azole adducts are C-3 epimers of the major product azole adducts.

### 2.6. Antimicrobial Assay

Since several sesquiterpene lactone-derived amino and thiol analogs exerted a significant antimicrobial effect [16,20], in vitro antimicrobial effects of the prepared amino and thiol analogs were tested [41] on three bacterial strains (*Staphylococcus aureus* ATCC 25923, *S. aureus* MRSA ATCC 43300, and *Escherichia coli* ATCC 25922). The results indicated that *β*-aminolactones **23**–**30** showed moderate activity on the *S. aureus* ATCC 25923 strain (the most active compound, naphthylmethyl-substituted *β*-aminolactone **30**, exhibited an MIC value of 12.5 μM on this Gram-positive bacterium), but they were not effective on the other two strains (Table 5). All other compounds lacked antibacterial activity (see Table S1 in Supplementary Materials for biological screening data of all compounds).

### 2.7. Docking Study

**Figure 6:**
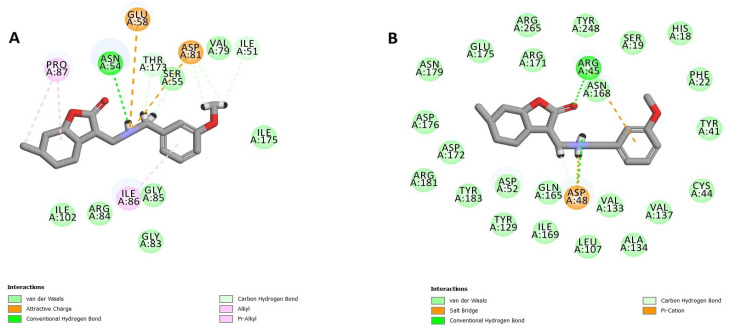
A 2D diagram of the interactions between compound **28** and the binding site of the protein templates ((**A**): 3U2D, (**B**): 2ZCQ).

**Figure 7:**
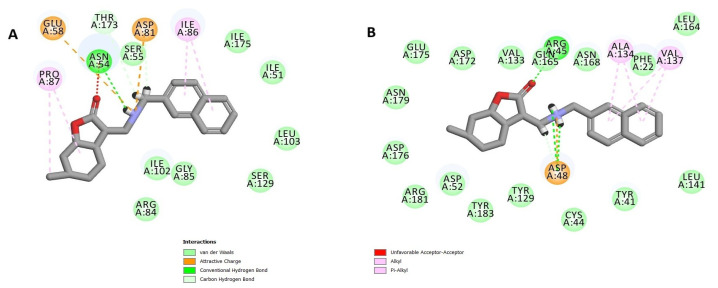
A 2D diagram of the interactions between compound **30** and the binding site of the protein templates ((**A**): 3U2D, (**B**): 2ZCQ).

Molecular docking was conducted to predict the possible protein targets of the most promising compounds **28** and **30** (see Table 5). To explore potential binding sites, a variety of *S. aureus* proteins with key roles in bacterial survival and resistance mechanisms were selected to be used as templates, including penicillin-binding protein 2a (PBP2a) (PDB code: 4CJN, resolution: 1.95 Å) [42], dihydrofolate reductase (DHFR) (PDB code: 2W9S, resolution: 1.80 Å) [43], DNA gyrase (GyrB ATPase domain) (PDB code: 3U2D, resolution: 1.85 Å) [44], and the C(30) carotenoid dehydrosqualene synthase (PDB code: 2ZCQ, resolution: 2.38 Å) [45]. The CDOCKER method [46] allowed us to investigate the ligand–receptor interactions and to predict binding energies (Table S2 in Supplementary Materials). Both compounds demonstrated favorable binding interactions with DNA gyrase (PDB code: 3U2D) and dehydrosqualene synthase (PDB code: 2ZCQ) (Figure 6 and Figure 7). Compound **28** showed robust binding to 2ZCQ through hydrogen bonding with ASP A:48 and ASN A:168, and a *π*–cation bonding with ARG A:45, achieving a CDOCKER interaction energy range of −47.5769 to −56.8457 kcal/mol. Similarly, compound **30** exhibited strong hydrogen bonding with ASN A:54, THR A:173, and ASP A:81 in 3U2D, accompanied by notable van der Waals interactions, resulting in a CDOCKER interaction energy range of −40.2136 to −47.49286 kcal/mol. These results suggest that the amino group plays a major role in forming strong interactions, such as hydrogen bonds and ionic interactions, with the residues within the binding site. Additionally, it should be noted that the naphthyl group contributes to forming further van der Waals bonds, which may explain why compound **30** has the highest activity among the other compounds. However, the role of the remaining functional groups is limited to minor interactions, which may explain the in vitro assay results. Furthermore, the in silico ADMET analysis [47] proposes promising pharmacokinetic and safety profiles for the tested compounds (Table S3 in Supplementary Materials).

## 3. Materials and Methods

### 3.1. General Methods

Commercially available solvents were used as obtained from suppliers (Molar Chemicals Ltd., Halásztelek, Hungary; Merck Ltd., Budapest, Hungary and VWR International Ltd., Debrecen, Hungary), while applied solvents were dried according to standard procedures. Optical rotations were measured in MeOH at 20 °C with a Perkin-Elmer 341 polarimeter (PerkinElmer Inc., Shelton, CT, USA). Chromatographic separations and monitoring of reactions were carried out on a Merck Kieselgel 60 (Merck Ltd., Budapest, Hungary). HRMS flow injection analysis was performed with a Thermo Scientific Orbitrap Exploris 240 hybrid quadrupole-Orbitrap (Thermo Fischer Scientific, Waltham, MA, USA) mass spectrometer coupled to a Waters Acquity I-Class UPLC TM (Waters, Manchester, UK). GC measurements for direct separation of commercially available enantiomers of isopulegol, to determine the enantiomeric purity of the starting material (−)-**21** and its enantiomer (+)-**21**, were performed on a Chirasil-DEX CB column (2500 × 0.25 mm I.D.) on a Perkin-Elmer Autosystem XL GC consisting of a Flame Ionization Detector (Perkin-Elmer Corp., Norwalk, CT, USA) and a Turbochrom Workstation data system (Perkin-Elmer Corp., Norwalk, CT, USA). Melting points were determined on a Kofler apparatus (Nagema, Dresden, Germany) and they are uncorrected. ^1^H NMR, ^13^C NMR, and ^19^F NMR were recorded on a Brucker Avance DRX 500 spectrometer [500 MHz (^1^H), 471 MHz (^19^F), and 125 MHz (^13^C)]. The ^1^H and ^13^C chemical shifts are given relative to TMS and ^19^F to CFCl_3_ (0.00 ppm). J values are given in Hz. Images of NMR spectra are found in Figures S1–S86 in the Supplementary Materials.

(−)-Isopulegol (−)-**21** is available commercially from Merck Co with *ee*% = 95%. (+)-Neoisopulegol (+)-**22** [36,38], (−)-*α*-methylene-*γ*-butyrolactone (–)-**20** [36,37], and cysteine derivatives (WO 2015148880 A1) were prepared according to modified literature procedures.

### 3.2. Preparation of (+)-Neoisopulegol (+)-***22***

Oxidation of (−)-isopulegol was based on Ref. [38]. To a slurry of PCC (27.6 g, 2 equiv.) and silica gel (55 g) in DCM (200 mL), (−)-isopulegol **9** (10 g, 64 mmol) was added. The reaction mixture was stirred at room temperature for 48 h. Upon completion of the reaction (as monitored by TLC), the mixture was filtered and the filtrate was evaporated to dryness. The crude product was purified by column chromatography on silica gel using *n*-hexane/EtOAc 9:1 as the eluent. This provided (–)-isopulegone as a pale yellow oil (yield: 80%).

Reduction of (–)-isopulegone was based on Ref. [36]. (–)-Isopulegone (2 g, 12.5 mmol) was dissolved in dry THF (40 mL). L-Selectride (15 mL, 1.2 equiv.) was dissolved in dry THF (20 mL), and added dropwise to the previous solution under an argon atmosphere at −78 °C. After 1–2 h, the reaction mixture was allowed to warm up to RT, and then it was treated with H_2_O_2_ in 5% NaOH (40 mL) and extracted with Et_2_O (3 × 100 mL). The organic layer was dried over Na_2_SO_4_ and evaporated to dryness. The crude product was purified by column chromatography on silica gel using *n*-hexane/EtOAc 9:1 as the eluent. This provided (+)-neoisopulegol (+)-**22** as a pale yellow oil (yield: 95%).

### 3.3. Preparation of (–)-α-Methylene-γ-Butyrolactone (−)-***20***

Hydroxylation of (+)-neoisopulegol was based on Ref. [37]. *t*-BuOK (2.2 g, 1.5 equiv.) was dissolved in dry *n*-hexane (15 mL). Then, *n*-BuLi (2 g, 2 equiv.) was added dropwise under an argon atmosphere at 0 °C. The mixture was stirred for one hour, during which the color changed from white to light orange. After the dropwise addition of (+)-neoisopulegol (+)-**22** (2 g, 13 mmol), the reaction mixture was stirred under an argon atmosphere at RT overnight. B(OMe)_3_ (4.5 mL, 3 equiv.) was added dropwise at −78 °C and stirred with the mixture for one hour. Upon reaching −20 °C, 30% aqueous H_2_O_2_ (7 mL) was introduced into the mixture and stirred for one hour. Water was then added to quench the reaction, and the mixture was extracted with Et_2_O (3 × 100 mL). The organic layer was dried over Na_2_SO_4_, filtered, and evaporated to dryness. The crude product was crystallized from *n*-hexane to give (1*S*,2*S*,5*R*)-2-(3-hydroxyprop-1-en-2-yl)-5-methylcyclohexanol as white crystals (yield: 50%).

Oxidation of (1*S*,2*S*,5*R*)-2-(3-hydroxyprop-1-en-2-yl)-5-methylcyclohexanol is based on Ref. [36]. To a suspension of MnO_2_ (29.6 g, 20 equiv.) in DCM (50 mL), a solution of (1*S*,2*S*,5*R*)-2-(3-hydroxyprop-1-en-2-yl)-5-methylcyclohexanol (3 g, 17 mmol) in DCM (50 mL) was added. The reaction mixture was vigorously stirred at 50–60 °C under reflux for 48 h. Upon completion of the reaction (monitored by TLC), the mixture was filtered through a Celite pad, and the filtrate was concentrated to afford the corresponding lactone (−)-**20** as a yellow oil (yield: 60%).

### 3.4. General Procedure for the Preparation of (+)-Neoisopulegol-Based β-Aminolactone Compounds ***23***–***30***

To a solution of compound (−)-**20** (50 mg, 0.3 mmol) in dry EtOH (1.0 mL), the appropriate amines (0.45 mmol, 1.5 equiv.) were added. The reaction mixture was stirred at room temperature for 24 h. After the completion of the reaction (as monitored by TLC), the mixture was evaporated to dryness. The crude product was purified by column chromatography on silica gel using CHCl_3_:MeOH = 39:1 as the eluent.

*(3S,3aS,6R,7aS)-3-((Benzylamino)methyl)-6-methylhexahydrobenzofuran-2(3H)-one (***23***)*: The reaction was accomplished with benzylamine. Yield: 89%; yellow oil; $\left[\alpha \right]\begin{array}{c} 20\\ D \end{array}$ = −44.45 (*c* = 0.15, MeOH). ^1^H NMR (500 MHz, CDCl_3_): δ = 7.34–7.30 (m, 4H), 7.28–7.22 (m, 1H), 4.45 (d, 1H, *J* = 2.7 Hz), 3.87–3. 76 (m, 2H), 2.99 (dd, *J* = 11.8, 7.1 Hz, 1H), 2.94–2.87 (1H, m), 2.69 (dd, *J* = 11.8, 7.4 Hz, 1H), 2.32 (ddd, *J* = 11.6, 9.9, 5.7 Hz, 1H), 2.22 (1H, d, *J* = 15 Hz), 1.66–1.60 (m, 2H), 1.60–1.51 (m, 1H), 1.08–1.28 (m, 2H), 0.94–0.81 (m, 4H). ^13^C NMR (125 MHz, CDCl_3_): δ = 178.6, 140.2, 128.6, 128.3, 127.2, 78.8, 54.4, 48.3, 44.9, 38.0, 36.2, 32.1, 26.4, 23.1, 22.1. HRMS (ESI): *m*/*z* calcd for C_17_H_24_NO_2_ [M+H]^+^: 274.1807; found: 274.1798.

*(3S,3aS,6R,7aS)-3-(((2-Fluorobenzyl)amino)methyl)-6-methylhexahydrobenzofuran-2(3H)-one (***24***)*: The reaction was accomplished with 2-fluorobenzylamine. Yield: 42%; yellow oil; $\left[\alpha \right]\begin{array}{c} 20\\ D \end{array}$ = −33.80 (*c* = 0.17, MeOH). ¹H NMR (500 MHz, CDCl_3_): δ = 7.34 (t, *J* = 7.5 Hz, 1H), 7.25–7.21 (m, 1H), 7.11 (t, *J* = 7.4 Hz, 1H), 7.07–7.01 (m, 1H), 4.45 (d, *J* = 2.6 Hz, 1H), 3.92–3.82 (m, 2H), 3.00 (dd, *J* = 11.8, 6.9 Hz, 1H), 2.91 (dd, *J* = 13.7, 6.8 Hz, 1H), 2.68 (dd, *J* = 11.7, 7.7 Hz, 1H), 2.33 (ddd, *J* = 11.4, 10.0, 5.7 Hz, 1H), 2.22 (d, *J* = 14.9 Hz, 1H), 1.69–1.61 (m, 2H), 1.61–1.51 (m, 1H), 1.23–1.08 (m, 2H), 0.94–0.81 (m, 4H). ^13^C NMR (125 MHz, CDCl_3_): δ 178.5, 161.4 (d, ^1^*J*_C-F_ = 245.1 Hz), 130.5 (d, ^3^*J*_C-F_ = 4.6 Hz), 129 (d, ^3^*J*_C-F_ = 8.1 Hz), 127 (d, ^2^*J*_C-F_ = 14.5 Hz), 124.3 (d, ^4^*J*_C-F_ = 3.6 Hz), 115.5 (d, ^2^*J*_C-F_ = 21.8 Hz), 78.8, 48.2, 47.6 (d, ^3^*J*_C-F_ = 2.9 Hz), 44.7, 37.9, 36.2, 32.1, 26.4, 23.1, 22.1. ^19^F NMR (470 MHz, CDCl_3_) δ = −119.46. HRMS (ESI): *m*/*z* calcd for C_17_H_23_FNO_2_ [M+H]^+^: 292.1713; found: 292.1701.

*(3S,3aS,6R,7aS)-3-(((3-Fluorobenzyl)amino)methyl)-6-methylhexahydrobenzofuran-2(3H)-one (***25***)*: The reaction was accomplished with 3-fluorobenzylamine. Yield: 39%; yellow oil; $\left[\alpha \right]\begin{array}{c} 20\\ D \end{array}$ = −36.11 (*c* = 0.18, MeOH). ¹H NMR (500 MHz, CDCl_3_): δ = 7.31–7.26 (m, 1H), 7.12–7.03 (m, 2H), 6.94 (td, *J* = 8.5, 2.1 Hz, 1H), 4.46 (d, *J* = 2.6 Hz, 1H), 3.87–3.75 (m, 2H), 2.98 (dd, *J* = 11.6, 7.3 Hz, 1H), 2.90 (dd, *J* = 13.6, 6.8 Hz, 1H), 2.67 (dd, *J* = 11.6, 7.1 Hz, 1H), 2.32 (ddd, *J* = 11.4, 10.0, 5.7 Hz, 1H), 2.22 (d, *J* = 14.9 Hz, 1H), 1.70–1.63 (m, 2H), 1.59–1.51 (m, 1H), 1.25–1.09 (m, 2H), 0.95–0.81 (m, 4H). ^13^C NMR (125 MHz, CDCl_3_): δ = 178.5, 163.0 (d, ^1^*J*_C-F_ = 245.7 Hz), 142.7 (d, ^3^*J*_C-F_ = 6.9 Hz), 129.9 (d, ^3^*J*_C-F_ = 8.2 Hz), 123.5 (d, ^4^*J*_C-F_ = 2.7 Hz), 114.8 (d, ^2^*J*_C-F_ = 21.2 Hz), 113.9 (d, ^2^*J*_C-F_ = 21.1 Hz), 78.7, 53.6 (d, ^4^*J*_C-F_ = 1.4 Hz), 48.1, 44.7, 37.9, 36.0, 31.9, 26.2, 23.0, 21.9. ^19^F NMR (470 MHz, CDCl_3_) δ = −113.46. HRMS (ESI): *m*/*z* calcd for C_17_H_23_FNO_2_ [M+H]^+^: 292.1713; found: 292.1703.

*(3S,3aS,6R,7aS)-3-(((4-Fluorobenzyl)amino)methyl)-6-methylhexahydrobenzofuran-2(3H)-one (***26***)*: The reaction was accomplished with 4-fluorobenzylamine. Yield: 52%; yellow oil; $\left[\alpha \right]\begin{array}{c} 20\\ D \end{array}$ = −44.45 (*c* = 0.16, MeOH). ^1^H NMR (500 MHz, CDCl_3_): δ = 7.29 (dd, *J* = 8.3, 5.6 Hz, 2H), 7.01 (t, *J* = 8.7 Hz, 2H), 4.46 (d, *J* = 2.7 Hz, 1H), 3.84–3.73 (m, 2H), 2.97 (dd, *J* = 11.5, 7.3 Hz, 1H), 2.91 (dd, *J* = 13.4, 6.7 Hz, 1H), 2.68 (dd, *J* = 11.5, 6.9 Hz, 1H), 2.32 (ddd, *J* = 11.5, 9.8, 5.7 Hz, 1H), 2.22 (d, *J* = 15.2 Hz, 1H), 1.69–1.59 (m, 2H), 1.59–1.51 (m, 1H), 1.28–1.07 (m, 2H), 0.94–0.81 (m, 4H). ^13^C NMR (125 MHz, CDCl_3_): δ = 178.5, 162.0 (d, ^1^*J*_C-F_ = 245.7 Hz), 135.5 (d, ^4^*J*_C-F_ = 3.2 Hz), 129.7 (d, ^3^*J*_C-F_ = 8.4 Hz), 115.3 (d, ^2^*J*_C-F_ = 21.3 Hz), 78.7, 53.4, 47.9, 44.6, 37.9, 36.0, 31.9, 26.2, 23.0, 21.9. ^19^F NMR (470 MHz, CDCl_3_) δ = −115.78. HRMS (ESI): *m*/*z* calcd for C_17_H_23_FNO_2_ [M+H]^+^: 292.1713; found: 292.1703.

*(3S,3aS,6R,7aS)-3-(((2-Methoxybenzyl)amino)methyl)-6-methylhexahydrobenzofuran-2(3H)-one (***27***)*: The reaction was accomplished with 2-methoxybenzylamine. Yield: 48% (35 mg); white powder, mp. 95.8–97.1 °C; $\left[\alpha \right]\begin{array}{c} 20\\ D \end{array}$= −31.60 (*c* = 0.19, MeOH). ^1^H NMR (500 MHz, CDCl_3_): δ = 7.25–7.21 (m, 2H), 6.91 (t, *J* = 7.4 Hz, 1H), 6.88–6.85 (m, 1H), 4.44 (d, *J* = 2.6 Hz, 1H), 3.84 (s, 3H), 3.81 (s, 2H), 2.98 (dd, *J* = 11.7, 6.5 Hz, 1H), 2.92 (dd, *J* = 6.5, 13.9 Hz, 1H), 2.66 (dd, *J* = 11.7, 7.9 Hz, 1H), 2.33 (ddd, *J* = 11.4, 9.9, 5.6 Hz, 1H), 2.21 (d, *J* = 13.3 Hz, 1H), 1.67–1.64 (m, 3H), 1.23–1.16 (m, 1H), 1.15–1.07 (m, 1H), 0.93–0.82 (m, 4H). ^13^C NMR (125 MHz, CDCl_3_): δ = 178.3, 129.8, 128.4, 128.0, 120.5, 110.3, 78.6, 55.3, 49.6, 48.1, 44.5, 37.8, 36.1, 32.0, 26.2, 22.8, 21.9. HRMS (ESI): *m*/*z* calcd for C_18_H_26_NO_3_ [M+H]^+^: 304.1913; found: 304.1901.

*(3S,3aS,6R,7aS)-3-(((3-Methoxybenzyl)amino)methyl)-6-methylhexahydrobenzofuran-2(3H)-one (***28***)*: The reaction was accomplished with 3-methoxybenzylamine. Yield: 66%; yellow oil; $\left[\alpha \right]\begin{array}{c} 20\\ D \end{array}$= −37.92 (*c* = 0.19, MeOH). ¹H NMR (500 MHz, CDCl_3_): δ = 7.23 (t, *J* = 7.9 Hz, 1H), 6.92–6.88 (m, 2H), 6.81–6.78 (m, 1H), 4.45 (d, *J* = 2.7 Hz, 1H), 3.84–3.74 (m, 5H), 2.99 (dd, *J* = 11.8, 7.1 Hz, 1H), 2.90 (q, *J* = 6.8 Hz, 1H), 2.68 (dd, *J* = 11.8, 7.3 Hz, 1H), 2.35–2.28 (m, 1H), 2.21 (d, *J* = 15.1 Hz, 1H), 1.69–1.63 (m, 2H), 1.58–1.51 (m, 1H), 1.24–1.09 (m, 2H), 0.91 (d, *J* = 6.6 Hz, 3H), 0.89–0.82 (m, 1H). ^13^C NMR (125 MHz, CDCl_3_): δ = 178.6, 160.0, 141.9, 129.6, 120.6, 113.7, 112.7, 78.8, 55.4, 54.3, 48.3, 44.9, 38.1, 36.2, 32.1, 26.4, 23.2, 22.1. HRMS (ESI): *m*/*z* calcd for C_18_H_26_NO_3_ [M+H]^+^: 304.1913; found: 304.1902.

*(3S,3aS,6R,7aS)-3-(((4-Methoxybenzyl)amino)methyl)-6-methylhexahydrobenzofuran-2(3H)-one (***29***)*: The reaction was accomplished with 4-methoxybenzylamine. Yield: 69%; white powder, mp. 48.5–50.5 °C; $\left[\alpha \right]\begin{array}{c} 20\\ D \end{array}$= −10.96 (*c* = 0.17, MeOH). ^1^H NMR (500 MHz, CDCl_3_): δ = 7.23 (d, *J* = 8.4 Hz, 2H), 6.86 (d, *J* = 8.5 Hz, 2H), 4.45 (d, *J* = 2.6 Hz, 1H), 3.80 (s, 3H), 3.79–3.70 (m, 2H), 2.97 (dd, *J* = 11.7, 7.0 Hz, 1H), 2.89 (dd, *J* = 13.5, 6.9Hz, 1H), 2.67 (dd, *J* = 11.7, 7.4Hz, 1H), 2.34–2.27 (m, 1H), 2.21 (d, *J* = 14.9 Hz, 1H), 1.68–1.63 (m, 2H), 1.58–1.50 (m, 1H), 1.23–1.08 (m, 2H), 0.91 (d, *J* = 6.5 Hz, 3H), 0.88–0.81 (m, 1H). ^13^C NMR (125 MHz, CDCl_3_): δ = 178.6, 158.9, 132.3, 129.4, 114.0, 78.8, 55.4, 53.8, 48.3, 44.7, 38.0, 36.2, 32.1, 26.4, 23.1, 22.1. HRMS (ESI): *m*/*z* calcd for C_18_H_26_NO_3_ [M+H]^+^: 304.1913; found: 304.1902.

*(3S,3aS,6R,7aS)-6-Methyl-3-(((naphthalen-1-ylmethyl)amino)methyl)hexahydro-benzofuran-2(3H)-one (***30***)*: The reaction was accomplished with 1-naphthylmethylamine. Yield: 90%; white crystals, mp. 62.1–64.5 °C; $\left[\alpha \right]\begin{array}{c} 20\\ D \end{array}$ = −40.88 (*c* = 0.18, MeOH). ^1^H NMR (500 MHz, CDCl_3_): δ = 8.13 (d, *J* = 8.3 Hz, 1H), 7.86 (d, *J* = 8.0 Hz, 1H), 7.77 (d, *J* = 8.1 Hz, 1H), 7.56–7.45 (m, 3H), 7.45–7.39 (m, 1H), 4.46–4.41 (m, 1H), 4.33–4.19 (m, 2H), 3.11 (dd, *J* = 11.8, 6.8Hz, 1H), 2.93 (dd, *J* = 13.8, 6.8 Hz, 1H), 2.80 (dd, *J* = 11.8, 7.8 Hz, 1H), 2.33–2.26 (m, 1H), 2.21 (d, *J* = 14.8 Hz, 1H), 1.65–1.48 (m, 3H), 1.23–1.10 (m, 2H), 0.93–0.80 (m, 4H). ^13^C NMR (125 MHz, CDCl_3_): δ = 178.6, 135.6, 134.1, 131.9, 128.9, 128.0, 126.3, 126.2, 125.8, 125.5, 123.8, 78.8, 52.1, 48.3, 45.3, 38.0, 36.2, 32.1, 26.4, 23.1, 22.1. HRMS (ESI): *m*/*z* calcd for C_21_H_26_NO_2_ [M+H]^+^: 324.1964; found: 324.1953.

### 3.5. General Procedure for the Preparation of (+)-Neoisopulegol-Based Thiol Adducts ***33***–***35*** and ***38***–***43***

To a solution of compound (−)-**20** (50 mg, 0.3 mmol) in dry EtOH (1.0 mL), the appropriate benzyl mercaptan derivatives or cysteine derivatives (0.45 mmol, 1.5 equiv.) and Et_3_N (0.84 mmol, 2.8 equiv.) were added. After stirring for 24 h at room temperature (as monitored by TLC), the mixture was evaporated to dryness. The crude product was purified by column chromatography on silica gel, eluted with appropriate solvent.

*(3R,3aS,6R,7aS)-3-((Benzylthio)methyl)-6-methylhexahydrobenzofuran-2(3H)-one (***33***)*: The reaction was accomplished with benzyl mercaptan and chromatographed by *n*-hexane/EtOAc 9:1. Yield: 80%, white powder, mp. 88.7–90.1 °C; $\left[\alpha \right]\begin{array}{c} 20\\ D \end{array}$ = −116.51 (*c* = 0.17, MeOH). ^1^H NMR (500 MHz, CDCl_3_): δ = 7.31 (d, *J* = 4.3 Hz, 4H), 7.27–7.22 (m, 1H), 4.37 (dd, *J* = 6.0, 3.2 Hz, 1H), 3.75 (s, 2H), 2.91 (dd, *J* = 13.0, 4.1 Hz, 1H), 2.78–2.71 (m, 1H), 2.46 (t, *J* = 12.3 Hz, 1H), 2.35–2.27 (m, 1H), 2.19 (d, *J* = 15.0 Hz, 1H), 1.68–1.59 (m, 2H), 1.56–1.48 (m, 1H), 1.22–1.14 (m, 1H), 1.02–0.92 (m, 1H), 0.91–0.80 (m, 4H). 13C NMR (125 MHz, CDCl3): δ = 177.3, 138.2, 128.9, 128.8, 127.4, 78.6, 47.7, 37.6, 37.2, 36.2, 31.9, 26.8, 26.3, 22.4, 22.0. HRMS (ESI): *m*/*z* calcd for C_17_H_23_O_2_S [M+H]^+^: 291.1419; found: 291.1410.

*(3R,3aS,6R,7aS)-3-(((4-Fluorobenzyl)thio)methyl)-6-methylhexahydrobenzofuran-2(3H)-one (***34***)*: The reaction was accomplished with 4-fluorobenzyl mercaptan and chromatographed by *n*-hexane/EtOAc 9:1. Yield: 76%; white powder, mp. 58.4–61.2 °C; $\left[\alpha \right]\begin{array}{c} 20\\ D \end{array}$ = −107.25 (*c* = 0.16, MeOH). ^1^H NMR (500 MHz, CDCl_3_): δ = 7.31–7.26 (m, 2H), 7.00 (t, *J* = 8.6 Hz, 2H), 4.40 (d, *J* = 2.5 Hz, 1H), 3.72 (s, 2H), 2.89 (dd, *J* = 12.9, 4.1 Hz, 1H),), 2.82–2.73 (m, 1H), 2.45 (t, *J* = 12.2 Hz, 1H), 2.32 (ddd, *J* = 11.5, 9.9, 5.5 Hz, 1H), 2.20 (d, *J* = 14.9 Hz, 1H), 1.70–1.61 (m, 2H), 1.54–1.49 (m, 1H), 1.24–1.14 (m, 1H), 1.05–0.93 (m, 1H), 0.92–0.83 (m, 4H). ^13^C NMR (125 MHz, CDCl_3_): δ = 177.2, 162.1 (d, ^1^*J*_C-F_ = 245.8 Hz), 133.9 (d, ^4^*J*_C-F_ = 3.0 Hz), 130.5 (d, ^3^*J*_C-F_ = 8.2 Hz), 115.6 (d, ^2^*J*_C-F_ = 21.5 Hz), 78.7, 47.7, 37.6, 36.5, 36.2, 31.9, 26.8, 26.3, 22.4, 22.0. ^19^F NMR (470 MHz, CDCl_3_) δ = −115.20. HRMS (ESI): *m*/*z* calcd for C_17_H_22_FO_2_S [M+H]^+^: 309.1325; found: 309.1317.

*(3R,3aS,6R,7aS)-3-(((4-Methoxybenzyl)thio)methyl)-6-methylhexahydrobenzofuran-2(3H)-one (***35***)*: The reaction was accomplished with 4-methoxybenzyl mercaptan and chromatographed by *n*-hexane/EtOAc 9:1. Yield: 73%; white powder, mp. 60.7–62.3 °C; $\left[\alpha \right]\begin{array}{c} 20\\ D \end{array}$ = −114.78 (*c* = 0.15, MeOH). ^1^H NMR (500 MHz, CDCl_3_): δ = 7.22 (d, *J* = 8.5 Hz, 2H), 6.84 (d, *J* = 8.5 Hz, 2H), 4.39 (d, *J* = 2.6 Hz, 1H), 3.80 (s, 3H), 3.70 (s, 2H), 2.90 (dd, *J* = 13.0, 4.1 Hz, 1H), 2.80–2.72 (m, 1H), 2.45 (t, *J* = 12.3 Hz, 1H), 2.37–2.28 (m, 1H), 2.20 (d, *J* = 15.1 Hz, 1H), 1.69–1.63 (m, 2H), 1.53–1.50 (m, 1H), 1.23–1.14 (m, 1H), 1.02–0.94 (m, 1H), 0.93–0.82 (m, 4H). ^13^C NMR (125 MHz, CDCl_3_): δ = 177.3, 159.0, 130.1, 130.0, 114.2, 78.7, 55.5, 47.8, 37.6, 36.6, 36.2, 31.9, 26.7, 26.3, 22.4, 22.0. HRMS (ESI): *m*/*z* calcd for C_18_H_25_O_3_S [M+H]^+^: 321.1524; found: 321.1514.

*(R)-2-Acetamido-3-((((3R,3aS,6R,7aS)-6-methyl-2-oxooctahydrobenzofuran-3-yl)-methyl)-thio)propanoic acid (***38***)*: The reaction was accomplished with *N*-acetyl-L-cysteine and chromatographed by CHCl_3_/CH_3_OH 19:1. Yield: 71%; white powder, mp. 136.2–138.3 °C; $\left[\alpha \right]\begin{array}{c} 20\\ D \end{array}$ = −51.80 (*c* = 0.17, MeOH). ^1^H NMR (500 MHz, DMSO-*d_6_*): δ = 7.63 (d, *J* = 6.5 Hz, 1H), 4.51 (s, 1H), 4.12 (d, *J* = 3.8 Hz, 1H), 3.13–3.06 (m, 1H), 3.04 (dd, *J* = 13.3, 3.7 Hz, 1H), 2.79 (dd, *J* =13.1, 3.9 Hz, 1H), 2.71 (dd, *J* = 13.2, 7.2 Hz, 1H),), 2.42 (t, *J* = 12.3 Hz, 1H), 2.34 (d, *J* = 4.6 Hz, 1H), 2.04 (d, *J* = 14.5 Hz, 1H), 1.84 (s, 3H), 1.67 (s, 1H), 1.58 (s, 1H), 1.38 (s, 1H), 1.23 (t, *J* = 12.1 Hz, 1H), 1.09 (t, *J* = 6.9 Hz, 1H), 0.87 (d, *J* = 6.3 Hz, 5H). ^13^C NMR (125 MHz, DMSO-*d_6_*): δ = 177.7, 173.4, 169.1, 78.1, 54.3, 47.2, 37.2, 35.8, 35.3, 31.8, 27.3, 26.5, 23.3, 22.5, 22.3. HRMS (ESI): *m*/*z* calcd for C_15_H_24_NO_5_S [M+H]^+^: 330.1375; found: 330.1367.

*(R)-Methyl 2-amino-3-((((3R,3aS,6R,7aS)-6-methyl-2-oxooctahydrobenzofuran-3-yl)-methyl)thio)propanoate (***39***)*: The reaction was accomplished with L-cysteine methyl ester and chromatographed by CHCl_3_/CH_3_OH 19:1. Yield: 50%; orange powder, mp. 78.4–80.3 °C; $\left[\alpha \right]\begin{array}{c} 20\\ D \end{array}$ = −134.73 (*c* = 0.15, MeOH). ^1^H NMR (500 MHz, CDCl_3_): δ =4.46 (dd, *J* = 5.9, 3.1 Hz, 1H), 3.75 (s, 3H), 3.68 (dd, *J* = 7.4, 4.6 Hz, 1H), 2.98 (ddd, *J* = 13.5, 6.7, 4.4 Hz, 2H), 2.94–2.88 (m, 1H), 2.80 (dd, *J* = 13.5, 7.4 Hz, 1H), 2.60 (dd, *J* = 12.8, 11.5 Hz, 1H), 2.40 (ddd, *J* = 11.9, 9.8, 5.9 Hz, 1H), 2.26–2.19 (m, 1H), 1.82–1.74 (m, 1H), 1.74–1.65 (m, 1H), 1.61–1.51 (m, 1H), 1.21 (ddd, *J* = 15.6, 12.5, 3.5 Hz, 1H), 1.06 (ddd, *J* = 25.6, 13.3, 3.3 Hz, 1H), 0.95–0.84 (m, 4H). ^13^C NMR (125 MHz, CDCl_3_): δ = 177.1, 174.5, 78.7, 54.3, 52.5, 48.0, 38.0, 37.5, 36.2, 31.9, 27.8, 26.3, 22.5, 22.0. HRMS (ESI): *m*/*z* calcd for C_14_H_24_NO_4_S [M+H]^+^: 302.1426; found: 302.1420.

*(R)-Methyl 2-acetamido-3-((((3R,3aS,6R,7aS)-6-methyl-2-oxooctahydrobenzofuran-3-yl)methyl)thio)propanoate (***40***)*: The reaction was accomplished with *N*-acetyl-L-cysteine methyl ester and chromatographed by *n*-hexane/EtOAc 1:2. Yield: 62%; white powder, mp. 105.4–107.6 °C; $\left[\alpha \right]\begin{array}{c} 20\\ D \end{array}$ = −104.50 (*c* = 0.16, MeOH). ^1^H NMR (500 MHz, CDCl_3_): δ = 6.32 (d, *J* = 6.9 Hz, 1H), 4.85 (dt, *J* = 7.4, 4.9 Hz, 1H), 4.46 (dd, *J_1_* = 5.8, 3.1 Hz, 1H), 3.79 (s, 3H), 3.13 (dd, *J* = 13.9, 4.8 Hz, 1H), 3.03–2.92 (m, 2H), 2.87 (ddd, *J* = 10.7, 5.9, 4.5 Hz, 1H), 2.58 (dd, *J* = 12.9, 11.1 Hz, 1H), 2.37 (ddd, *J* = 11.9, 9.8, 5.9 Hz, 1H), 2.26–2.18 (m, 1H), 2.06 (s, 3H), 1.78–1.66 (m, 2H), 1.60–1.51 (m, 1H), 1.25–1.17 (m, 1H), 1.06 (ddd, *J* = 25.4, 13.2, 3.2 Hz, 1H), 0.95–0.82 (m, 4H). ^13^C NMR (125 MHz, CDCl_3_): δ = 177.0, 171.3, 170.0, 78.6, 53.0, 52.4, 48.0, 37.6, 36.1, 34.9, 31.9, 28.1, 26.3, 23.3, 22.5, 22.0. HRMS (ESI): *m*/*z* calcd for C_16_H_26_NO_5_S [M+H]^+^: 344.1532; found: 344.1522.

*(R)-2-Acetamido-3-((((3R,3aS,6R,7aS)-6-methyl-2-oxooctahydrobenzofuran-3-yl)-methyl)thio)propanamide (***41***)*:The reaction was accomplished with *N*-acetyl-L-cysteine amide and chromatographed by *n*-hexane/EtOAc 1:2. Yield: 41%; white powder, mp. 99.3–101.2 °C; $\left[\alpha \right]\begin{array}{c} 20\\ D \end{array}$ = −116.83 (*c* = 0.12, MeOH). ^1^H NMR (500 MHz, DMSO-*d_6_*): δ = 8.04 (d, *J* = 8.3 Hz, 1H), 7.52 (s, 1H), 7.12 (s, 1H), 4.51 (s, 1H), 4.38 (dd, *J* = 12.6, 6.8 Hz, 1H), 3.15–3.08 (m, 1H), 2.88 (ddd, *J* = 17.5, 13.4, 4.7 Hz, 2H), 2.61 (dd, *J* = 13.6, 8.8 Hz, 1H), 2.46 (d, *J* = 12.4 Hz, 1H), 2.39–2.30 (m, 1H), 2.05 (d, *J* = 14.6 Hz, 1H), 1.85 (s, 3H), 1.74–1.62 (m, 1H), 1.58 (d, *J* = 7.9 Hz, 1H), 1.39 (s, 1H), 1.29–1.19 (m, 1H), 0.95–0.82 (m, 5H). ^13^C NMR (125 MHz, DMSO-*d_6_*): δ = 177.5, 172.7, 169.7, 78.1, 52.3, 47.0, 37.2, 35.8, 34.4, 31.8, 27.0, 26.5, 23.0, 22.5, 22.3. HRMS (ESI): *m*/*z* calcd for C_15_H_25_N_2_O_4_S [M+H]^+^: 329.1535; found: 329.1525.

*(R)-Ethyl 2-acetamido-3-((((3R,3aS,6R,7aS)-6-methyl-2-oxooctahydrobenzofuran-3-yl)-methyl)thio)propanoate (***42***)*: The reaction was accomplished with *N*-acetyl-L-cysteine ethyl ester and chromatographed by *n*-hexane/EtOAc 1:2. Yield: 93%; white powder, mp. 98.3–100.7 °C; $\left[\alpha \right]\begin{array}{c} 20\\ D \end{array}$ = −95.53 (*c* = 0.17, MeOH). ^1^H NMR (500 MHz, CDCl_3_): δ = 6.31 (d, *J* = 6.9 Hz, 1H), 4.82 (dt, *J* = 7.3, 4.9 Hz,1H), 4.46 (d, *J* = 2.8 Hz, 1H), 4.24 (q, *J* = 7.1 Hz, 2H), 3.13 (dd, *J* = 13.9, 4.7 Hz, 1H), 2.97 (dd, *J_1_* = 13.2, 4.5 Hz, 2H), 2.88–2.84 (m, 1H), 2.58 (dd, *J* = 12.9, 11.3 Hz, 1H), 2.37 (ddd, *J* = 11.9, 9.8, 5.8 Hz, 1H), 2.22 (dd, *J* = 15.0, 3.0 Hz, 1H), 2.05 (s, 3H), 1.77–1.72 (m, 1H), 1.72–1.65 (m, 1H), 1.60–1.50 (m, 1H), 1.31 (t, *J* = 7.1 Hz, 3H), 1.24–1.18 (m, 1H), 1.05 (qd, *J* = 13.2, 3.1 Hz, 1H), 0.96–0.84 (m, 4H). ^13^C NMR (125 MHz, CDCl_3_): δ = 177.0, 170.8, 170.0, 78.6, 62.2, 52.5, 48.0, 37.6, 36.2, 34.8, 31.9, 28.1, 26.3, 23.3, 22.5, 22.0, 14.3. HRMS (ESI): *m*/*z* calcd for C_17_H_28_NO_5_S [M+H]^+^: 358.1688; found: 358.1677.

*(R)-Methyl 2-((tert-butoxycarbonyl)amino)-3-((((3R,3aS,6R,7aS)-6-methyl-2-oxooctahydrobenzofuran-3-yl)methyl)thio)propanoate (***43***)*: The reaction was accomplished with N-Boc-L-cysteine methyl ester and chromatographed by *n*-hexane/EtOAc 2:1. Yield: 54%; white powder, mp. 88.3–90.3 °C; $\left[\alpha \right]\begin{array}{c} 20\\ D \end{array}$ = −82.13 (c = 0.19, MeOH). ^1^H NMR (500 MHz, CDCl_3_): δ = 5.33 (d, *J* = 6.2 Hz, 1H), 4.56 (d, *J* = 3.2 Hz, 1H), 4.45 (d, *J* = 2.7 Hz, 1H), 3.78 (s, 3H), 3.08 (dd, *J* = 13.8, 4.2 Hz, 1H), 3.01–2.92 (m, 2H), 2.91–2.84 (m, 1H), 2.65–2.58 (m, 1H), 2.37 (ddd, *J* = 11.8, 9.8, 5.8 Hz, 1H), 2.25–2.19 (m, 1H), 1.81–1.74 (m, 1H), 1.72–1.64 (m, 1H), 1.61–1.51 (m, 1H), 1.45 (s, 9H), 1.25–1.16 (m, 1H), p1.06 (ddd, *J* =25.7, 13.2, 3.2 Hz, 1H), 0.97–0.84 (m, 4H). ^13^C NMR (125 MHz, CDCl_3_): δ = 176.8, 171.3, 155.1, 80.3, 78.5, 53.5, 52.7, 47.9, 37.4, 36.0, 35.3, 31.8, 28.3, 28.0, 26.2, 22.3, 21. 9. HRMS (ESI): *m*/*z* calcd for C_19_H_32_NO_6_S [M+H]^+^: 402.1950; found: 402.1941.

### 3.6. General Procedure for the Preparation of (+)-Neoisopulegol-Based Azole Adducts ***44***–***48***

Compound (−)-**20** (50 mg, 0.3 mmol) and the azole derivative (0.6 mmol, 2 equiv.) were dissolved in acetonitrile (5.0 mL), and then DBU (0.3 mmol, 1 equiv.) was added. The mixture was stirred at 70 °C overnight. Then, it was evaporated to dryness, and the crude product was purified by column chromatography on silica gel with an appropriate solvent mixture.

*(3S,3aS,6R,7aS)-3-((1H-Imidazol-1-yl)methyl)-6-methylhexahydrobenzofuran-2(3H)-one (***44a***)*: The reaction was accomplished with imidazole and chromatographed by CHCl_3_/isopropanol 19:1. Yield: 50%; white crystals, mp. 43.5–44.3 °C; $\left[\alpha \right]\begin{array}{c} 20\\ D \end{array}$ = −73.36 (c = 0.15, MeOH). ^1^H NMR (500 MHz, CDCl_3_): δ = 7.52 (s, 1H), 7.10 (s, 1H), 6.98 (s, 1H), 4.48 (d, *J* =2.7 Hz, 1H), 4.41 (dd, *J* = 14.7, 4.4 Hz, 1H), 4.07 (dd, *J* = 14.7, 9.8 Hz, 1H), 3.14–3.08 (m, 1H), 2.33–2.22 (m, 2H), 1.83–1.71 (m, 2H), 1.63–1.61 (m, 1H), 1.28–1.18 (m, 2H), 0.98–0.86 (m, 4H). ^13^C NMR (125 MHz, CDCl_3_): δ = 130.4, 78.7, 49.9, 42.5, 37.4, 36.0, 31.8, 26.1, 23.2, 21.9. HRMS (ESI): *m*/*z* calcd for C_13_H_19_N_2_O_2_ [M+H]^+^: 235.1447; found: 235.1438.

*(3R,3aS,6R,7aS)-3-((1H-Imidazol-1-yl)methyl)-6-methylhexahydrobenzofuran-2(3H)-one (***44b***)*: The reaction was accomplished with imidazole and chromatographed by CHCl_3_/isopropanol 19:1. Yield: 14%; white crystals, mp. 150.2–152.3 °C; $\left[\alpha \right]\begin{array}{c} 20\\ D \end{array}$ = −45.30 (c = 0.20, MeOH). ^1^H NMR (500 MHz, CDCl_3_): δ =7.5 (s, 1H), 7.11 (s, 1H), 6.96 (s, 1H), 4.31–4.21 (m, 2H), 4.18 (q, *J*= 4.2 Hz, 1H), 2.71–2.65 (m, 1H), 2.09–2.02 (m, 2H), 1.82–1.71 (m, 1H), 1.71–1.56 (m, 2H), 1.33–1.19 (m, 2H), 1.00–0.87 (m, 4H). ^13^C NMR (125 MHz, CDCl_3_): δ = 176.5, 130.8, 78.0, 50.8, 45.5, 37.3, 35.7, 31.0, 27.0, 25.9, 21.2. HRMS (ESI): *m*/*z* calcd for C_13_H_19_N_2_O_2_ [M+H]^+^: 235.1447; found: 235.1438.

*(3S,3aS,6R,7aS)-3-((1H-1,2,4-Triazol-1-yl)methyl)-6-methylhexahydrobenzofuran-2(3H)-one (***45a***)*: The reaction was accomplished with 1,2,4-triazole and chromatographed by CHCl_3_/isopropanol 19:1. Yield: 57%; colorless oil; $\left[\alpha \right]\begin{array}{c} 20\\ D \end{array}$ = −75.33 (c = 0.18, MeOH). ^1^H NMR (500 MHz, CDCl_3_): δ = 8.17 (s, 1H), 7.95 (s, 1H), 4.58 (dd, *J* = 14.3, 4.8 Hz, 1H), 4.51 (d, *J*=15.0 Hz, 1H), 4.28 (dd, *J* = 14.3, 9.4 Hz, 1H), 3.34–3.30 (m, 1H), 2.43–2.34 (m, 1H), 2.25 (d, *J* = 15.0 Hz, 1H), 1.92–1.87 (m, 1H), 1.77–1.70 (m, 1H), 1.64–1.56 (m, 1H), 1.26 (s, 1H), 1.24–1.18 (m, 1H), 0.98–0.86 (m, 4H). ^13^C NMR (125 MHz, CDCl_3_): δ = 175.9, 152.6, 78.9, 48.5, 45.0, 37.6, 36.0, 31.8, 26.2, 23.1, 21.9. HRMS (ESI): *m*/*z* calcd for C_12_H_18_N_3_O_2_ [M+H]^+^: 236.1399; found: 236.1390.

*(3R,3aS,6R,7aS)-3-((1H-1,2,4-Triazol-1-yl)methyl)-6-methylhexahydrobenzofuran-2(3H)-one (***45b***)*: The reaction was accomplished with 1,2,4-triazole and chromatographed by CHCl_3_/isopropanol 19:1. Yield: 18%; white crystals, mp. 102.5–103.1 °C; $\left[\alpha \right]\begin{array}{c} 20\\ D \end{array}$ = −42.11 (*c* =0.17, MeOH). ^1^H NMR (500 MHz, CDCl_3_): δ = 8.12 (s, 1H), 7.96 (s, 1H), 4.52–4.41 (m, 2H), 4.38 (q, *J*= 4.3 Hz, 1H), 2.86–2.80 (m, 1H), 2.35–2.30 (m, 1H), 2.07 (d, *J*= 14.7 Hz, 1H), 1.81–1.75 (m, 1H), 1.72–1.60 (m, 2H), 1.36–1.22 (m, 2H), 1.01–0.96 (m, 1H), 0.93 (d, *J*= 6.58 Hz, 3H). ^13^C NMR (125 MHz, CDCl_3_): δ = 176.2, 152.6, 143.7, 78.1, 50.0, 47.7, 37.2, 35.6, 30.9, 26.8, 26.0, 21.2. HRMS (ESI): *m*/*z* calcd for C_12_H_18_N_3_O_2_ [M+H]^+^: 236.1399; found: 236.1390.

*(3S,3aS,6R,7aS)-3-((1H-1,2,3-Triazol-1-yl)methyl)-6-methylhexahydrobenzofuran-2(3H)-one (***46a***)*: The reaction was accomplished with 1,2,3-triazole and chromatographed by CHCl_3_/isopropanol 19:1. Yield: 53%; white crystals, mp. 99.3–101.2 °C; $\left[\alpha \right]\begin{array}{c} 20\\ D \end{array}$ = −82.49 (*c* =0.18, MeOH). ^1^H NMR (500 MHz, CDCl_3_): δ = 7.71 (d, *J*= 11.5 Hz, 2H), 4.69 (dd, *J*= 14.4, 4.9 Hz, 1H), 4.57 (dd, *J*= 14.4, 9.1 Hz, 1H), 4.51 (d, *J*= 2.7 Hz, 1H), 3.34 (dt, *J*= 9.2, 5.5 Hz, 1H), 2.45 (ddd, *J* = 11.9, 9.9, 5.9 Hz, 1H), 2.26–2.25 (d, *J*= 15.2 Hz, 1H), 1.98–1.93 (m, 1H), 1.77–1.70 (m, 1H), 1.65–1.57 (m, 1H), 1.29–1.16 (m, 2H), 0.98–0.87 (m, 4H). ^13^C NMR (125 MHz, CDCl_3_): δ = 175.9, 134.1, 124.4, 79.0, 49.02, 45.7, 37.9, 36.0, 31.8, 26.2, 23.1, 21.9. HRMS (ESI): *m*/*z* calcd for C_12_H_18_N_3_O_2_ [M+H]^+^: 236.1399; found: 236.1390.

*(3R,3aS,6R,7aS)-3-((1H-1,2,3-Triazol-1-yl)methyl)-6-methylhexahydrobenzofuran-2(3H)-one (***46b***)*: The reaction was accomplished with 1,2,3-triazole and chromatographed by CHCl_3_/isopropanol 19:1. Yield: 28%; white crystals, mp. 101.8–104.3 °C; $\left[\alpha \right]\begin{array}{c} 20\\ D \end{array}$ = −46.11 (*c* = 0.15, MeOH). ^1^H NMR (500 MHz, CDCl_3_): δ = 7.73 (s, 1H), 7.64 (s, 1H), 4.75–4.67 (m, 2H), 4.23 (dd, *J* = 8.7, 4.3 Hz, 1H), 2.80 (td, *J* = 6.2, 3.0 Hz, 1H), 2.36–2.31 (m, 1H), 2.03 (d, *J*= 15.0 Hz, 1H), 1.81–1.75 (m, 1H), 1.70–1.58 (m, 2H), 1.35–1.22 (m, 2H), 1.02–0.94 (m, 1H), 0.91 (d, *J*= 6.6 Hz, 3H). ^13^C NMR (125 MHz, CDCl_3_): δ = 176.3, 134.7, 124.1, 78.3, 50.3, 48.2, 37.1, 35.6, 30.8, 26.8, 26.0, 21.1. HRMS (ESI): *m*/*z* calcd for C_12_H_18_N_3_O_2_ [M+H]^+^: 236.1399; found: 236.1390.

*(3S,3aS,6R,7aS)-3-((1H-Benzo[d]imidazol-1-yl)methyl)-6-methylhexahydrobenzofuran-2(3H)-one (***47a***)*: The reaction was accomplished with benzimidazole and chromatographed by CHCl_3_/*t*-BuOH 19:1. Yield: 20%; white crystals, mp. 120.1–122.9 °C; $\left[\alpha \right]\begin{array}{c} 20\\ D \end{array}$ = −74.60 (*c* = 0.22, MeOH). ^1^H NMR (500 MHz, CDCl_3_): δ = 7.97 (s, 1H), 7.83 (d, *J* = 7.3 Hz, 1H), 7.42 (d, *J* = 7.9 Hz, 1H), 7.36–7.29 (m, 2H), 4.64 (dd, *J* = 15.0, 4.6 Hz, 1H), 4.45 (d, *J* = 2.65 Hz, 1H), 4.31 (dd, *J* = 15.0, 9.3 Hz, 1H), 3.31–3.27 (m, 1H), 2.28–2.23 (m, 2H), 1.89–1.86 (m, 1H), 1.77–1.75 (m, 1H), 1.67–1.60 (m, 1H), 1.37–1.16 (m, 2H), 0.94–0.87 (m, 4H). ^13^C NMR (125 MHz, CDCl_3_): δ = 176.0, 144.1, 143.0, 133.4, 123.5, 122.7, 121.0, 109.4, 78.7, 48.1, 40.6, 37.4, 36.0, 31.8, 26.1, 23.3, 21.9. HRMS (ESI): *m*/*z* calcd for C_17_H_21_N_2_O_2_ [M+H]^+^: 285.1603; found: 285.1592.

*(3R,3aS,6R,7aS)-3-((1H-Benzo[d]imidazol-1-yl)methyl)-6-methylhexahydrobenzofuran-2(3H)-one (***47b***)*: The reaction was accomplished with benzimidazole and chromatographed by CHCl_3_/*t*-BuOH 19:1. Yield: 12%; white powder, mp. 125.3–126.5 °C; $\left[\alpha \right]\begin{array}{c} 20\\ D \end{array}$ = −22.77 (*c* = 0.21, MeOH). ^1^H NMR (500 MHz, CDCl_3_): δ = 7.92 (s, 1H), 7.83 (d, *J* = 7.5 Hz, 1H), 7.44 (d, *J* = 7.8 Hz, 1H), 7.36–7.31 (m, 2H), 4.54–4.40 (m, 3H), 2.87 (ddd, *J* = 7.9, 5.1, 2.7 Hz, 1H), 2.14–2.02 (m, 2H), 1.71–1.62 (m, 2H), 1.58 (d, *J* = 3.4 Hz, 1H), 1.32–1.17 (m, 2H), 0.95–0.86 (m, 4H). ^13^C NMR (125 MHz, CDCl_3_): δ = 142.8, 141.7, 123.6, 122.7, 120.8, 109.2, 77.8, 49.7, 43.1, 37.3, 35.5, 30.7, 26.7, 25.7, 20.9. HRMS (ESI): *m*/*z* calcd for C_17_H_21_N_2_O_2_ [M+H]^+^: 285.1603; found: 285.1592.

*(3S,3aS,6R,7aS)-3-((1H-Benzo[d][1,2,3]triazol-1-yl)methyl)-6-methylhexahydrobenzo-furan-2(3H)-one (***48a***)*: The reaction was accomplished with benzotriazole and chromatographed by CHCl_3_/*t*-BuOH 19:1. Yield: 21%; white powder, mp. 129.8–131.7 °C; $\left[\alpha \right]\begin{array}{c} 20\\ D \end{array}$ = −91.04 (*c* = 0.15, MeOH). ^1^H NMR (500 MHz, CDCl_3_): δ = 8.09 (d, *J* = 8.35 Hz, 1H), 7.59 (d, *J* = 8.3 Hz, 1H), 7.54 (t, *J* = 7.6 Hz, 1H), 7.41 (t, *J* = 7.6 Hz, 1H), 4.94 (dd, *J* = 14.6, 3.8 Hz, 1H), 4.77 (dd, *J* = 14.6, 10.7 Hz, 1H), 4.52 (d, *J* = 2.6 Hz, 1H), 3.58–3.52 (m, 1H), 2.41 (ddd, *J* = 11.8, 9.9, 5.9 Hz, 1H), 2.26 (d, *J =* 15.2 Hz, 1H), 2.08–2.02 (m, 1H), 1.78–1.70 (m, 1H), 1.68–1.58 (m, 1H), 1.34–1.18 (m, 2H), 0.97–0.86 (m, 4H). ^13^C NMR (125 MHz, CDCl_3_): δ = 176.0, 127.9, 124.4, 120.4, 109.2, 79.1, 48.3, 43.4, 37.6, 36.0, 31.8, 26.2, 23.1, 22.0. HRMS (ESI): *m*/*z* calcd for C_16_H_20_N_3_O_2_ [M+H]^+^: 286.1556; found: 286.1546.

*(3R,3aS,6R,7aS)-3-((1H-Benzo[d][1,2,3]triazol-1-yl)methyl)-6-methylhexahydrobenzo-furan-2(3H)-one (***48b***)*: The reaction was accomplished with benzotriazole and chromatographed by CHCl_3_/*t*-BuOH 19:1. Yield: 12%; white crystals, mp. 135.9–137.5 °C; $\left[\alpha \right]\begin{array}{c} 20\\ D \end{array}$ = −38.03 (*c* = 0.19, MeOH). ^1^H NMR (500 MHz, CDCl_3_): δ = 8.08 (d, *J*= 8.4 Hz, 1H), 7.61 (d, *J* = 8.3 Hz, 1H), 7.54 (t, *J* = 7.6 Hz, 1H), 7.41 (t, *J* = 7.6 Hz, 1H), 4.94 (qd, *J* = 14.6, 6.7 Hz, 2H), 4.36 (q, *J* = 3.9 Hz, 1H), 2.94–2.92 (m, 1H), 2.4–2.36 (m, 1H), 2.08 (d, *J* = 15.5 Hz, 1H), 1.69–1.58 (m, 2H), 1.28–1.16 (m, 3H), 0.94–0.86 (m, 4H). ^13^C NMR (125 MHz, CDCl_3_): δ = 128.3, 124.6, 120.4, 109.4, 78.5, 50.3, 46.2, 37.2, 35.7, 31.3, 27.3, 25.9, 21.4. HRMS (ESI): *m*/*z* calcd for C_16_H_20_N_3_O_2_ [M+H]^+^: 286.1556; found: 286.1545.

### 3.7. General Procedure for the Ring Opening of Lactones with NH_3_ (Synthesis of ***31***, ***36***, and ***49a***–***b***)

To a solution of lactone **23**, **33**, or **47a**–**b** (0.36 mmol) in MeOH (2.0 mL), a solution of 25% NH_3_ in MeOH (5.0 mL) was added. The mixture was stirred at room temperature for 12 h. After the completion of the reaction (as monitored by TLC), the mixture was evaporated to dryness. The crude product was purified by column chromatography on silica gel then recrystallized in Et_2_O, resulting in compounds **31**, **36,** and **49a**–**b**, respectively.

*(S)-3-(Benzylamino)-2-((1S,2S,4R)-2-hydroxy-4-methylcyclohexyl)propanamide (***31***)*: Recrystallized in Et_2_O. Yield: 94%; white powder; mp. 140.3–142.5 °C; $\left[\alpha \right]\begin{array}{c} 20\\ D \end{array}$ = +27.52 (*c* = 0.20, MeOH). ¹H NMR (500 MHz, CD_3_OD): δ = 7.31 (dd, *J* = 7.3, 5.9 Hz, 4H), 7.27–7.22 (m, 1H), 3.81–3.70 (m, 2H), 3.40 (td, *J* = 10.6, 4.2 Hz, 1H), 2.98–2. 89 (m, 2H), 2.71–2.65 (m, 1H), 1.95 (d, *J* = 12.2 Hz, 1H), 1.71–1.55 (m, 3H), 1.46–1.34 (m, 1H), 1.15–1.04 (m, 1H), 0.96 (dd, *J* = 23.4, 12.0 Hz, 1H), 0.91 (d, *J* = 6.5 Hz, 3H), 0.89–0.79 (m, 1H).¹³C NMR (125 MHz, CD_3_OD) δ = 129.5, 129.5, 128.2, 71.6, 54.6, 48.3, 47.1, 46.7, 45.7, 35.7, 32.8, 27.8, 22.5. HRMS (ESI) *m*/*z*: calcd for C_17_H_27_N_2_O_2_[M+H]^+^: 291.2073; found: 291.2063.

*(R)-3-(Benzylthio)-2-((1S,2S,4R)-2-hydroxy-4-methylcyclohexyl)propanamide (***36***):* Chromatographed by CHCl_3_/MeOH 19:1. Yield: 95%; white powder, mp. 156.3–158.6 °C; $\left[\alpha \right]\begin{array}{c} 20\\ D \end{array}$ = +86.75 (*c* = 0.16, MeOH). ^1^H NMR (500 MHz, CDCl_3_): δ = 7.36–7.29 (m, 4H), 7.27–7.23 (m, 1H), 5.62 (s, 1H), 5.39 (s, 1H), 3.94 (d, *J* = 1.8 Hz, 1H), 3.78–3.69 (m, 2H), 2.71 (d, *J* = 7.1 Hz, 2H), 2.32 (dd, *J* = 15.5, 7.1, 1H), 1.79 (ddd, *J* = 13.8, 5.9, 3.3 Hz, 1H), 1.75–1.63 (m, 2H), 1.59–1.51 (m, 1H), 1.51–1.44 (m, 1H), 1.44–1.33 (m, 1H), 1.17–1.10 (m, 1H), 0.94–0.83 (m, 4H). ^13^C NMR (125 MHz, CDCl_3_): δ = 176.6, 138.7, 129.2, 128.7, 127.3, 67.5, 49.4, 43.3, 42.5, 37.3, 34.6, 31.9, 25.9, 24.6, 22.3. HRMS (ESI) *m*/*z*: calcd for C_17_H_26_NO_2_S [M+H]^+^: 308.1684; found: 308.1675.

*(S)-3-(1H-Benzo[d]imidazol-1-yl)-2-((1S,2S,4R)-2-hydroxy-4-methylcyclohexyl)-propanamide (***49a***)*: Chromatographed by CHCl_3_/acetone 9:1. Yield: 59%; mp. 263.3–265.0 °C; $\left[\alpha \right]\begin{array}{c} 20\\ D \end{array}$ = +89.60 (*c* = 0.20, MeOH). ^1^H NMR (500 MHz, CD_3_OD): δ = 8.00 (s, 1H), 7.64 (t, *J* = 8.2 Hz, 2H), 7.31 (t, *J* = 7.6 Hz, 1H), 7.26 (t, *J* = 7.6 Hz, 1H), 4.58 (dd, *J* = 13.9, 3.8 Hz, 1H), 4.47 (dd, *J* = 22.0, 10.3 Hz, 2H), 4.22 (s, 1H), 2.99 (td, *J_1_* = 10.9, 3.7 Hz, 1H), 1.94 (dd, *J* = 13.9, 2.4 Hz, 1H), 1.89–1.79 (m, 1H), 1.76–1.67 (m, 1H), 1.60–1.52 (m, 2H), 1.52–1.45 (m, 1H), 1.32–1.20 (m, 1H), 0.98 (td, *J* = 12.6, 3.9 Hz, 1H),

0.95–0.90 (m, 3H). ^13^C NMR (125 MHz, CD_3_OD): δ = 124.2, 123.5, 120.0, 111.8, 66.9, 50.3, 43.7, 42.6, 35.7, 26.9, 26.4, 22.7. HRMS (ESI) *m*/*z*: calcd for C_17_H_24_N_3_O_2_ [M+H]^+^: 302.1868; found: 302.1859.

*(R)-3-(1H-Benzo[d]imidazol-1-yl)-2-((1S,2S,4R)-2-hydroxy-4-methylcyclohexyl)-propanamide (***49b***)*: Chromatographed by CHCl_3_/acetone 9:1. Yield: 69%; white crystals, mp. 240.4–243.3 °C; $\left[\alpha \right]\begin{array}{c} 20\\ D \end{array}$ = −48.20 (*c* = 0.20, MeOH). ^1^H NMR (500 MHz, CD_3_OD): δ = 0.92 (d, *J* = 6.3 Hz, 3H), 0.99–1.11 (m, 1H), 1.15–1.21 (m, 1H), 1.66–1.80 (m, 2H), 1.80–1.95 (m, 4H), 3.03 (ddd, *J_1_* = 4.5 Hz, *J_2_* = 8.0 Hz, *J_3_* = 12.0 Hz, 1H), 4.01 (s, 1H), 4.50–4.62 (m, 2H), 7.28 (t, *J* = 7.6 Hz, 1H), 7.34 (t, *J* = 7.6 Hz, 1H), 7.60 (d, *J* = 8.1 Hz, 1H), 7.66 (d, *J* = 8.0 Hz, 1H), 8.04 (s, 1H). ^13^C NMR (125 MHz, CD_3_OD): δ = 124.3, 123.5, 120.1, 111.6, 68.9, 50.8, 46.3, 43.7, 43.5, 35.9, 26.8, 24.8, 22.6. HRMS (ESI) *m*/*z*: calcd for C_17_H_24_N_3_O_2_ [M+H]^+^: 302.1868; found: 302.1859.

### 3.8. General Procedure for the Ring Opening of Lactones with Benzylamine (Synthesis of ***32***, ***37***, and ***50a***–***b***)

To a solution of lactone **23**, **33**, or **47a–b** (0.36 mmol) in dry THF (5.0 mL), benzylamine (0.72 mmol) was added. The mixture was stirred at 70 °C for 48 h. When the reaction was completed, the mixture was evaporated to dryness. The crude product was purified by column chromatography on silica gel mixture, then recrystallized in *n*-hexane/Et_2_O, resulting in compounds **32**, **37**, and **50a–b**, respectively.

*(S)-N-Benzyl-3-(benzylamino)-2-((1S,2S,4R)-2-hydroxy-4-methylcyclohexyl)-propanamide (***32***)*: Chromatographed by CHCl_3_/CH_3_OH 19:1. Yield: 70%; white powder, mp. 211.0–212.7 °C; $\left[\alpha \right]\begin{array}{c} 20\\ D \end{array}$ = +21.05 (*c* = 0.20, MeOH). ^1^H NMR (500 MHz, DMSO-*d_6_*): δ = 9.18 (s, 1H), 9.06 (s, 1H), 8.75 (t, *J* = 5.7 Hz, 1H), 7.54 (d, *J* = 4.0 Hz, 2H), 7.42 (d, *J* = 4.9 Hz, 3H), 7.35–7.20 (m, 5H), 4.74 (s, 1H), 4.43–4.15 (m, 2H), 4.11 (s, 2H), 3.80 (s, 1H), 3.23–3.12 (m, 1H), 3.05 (d, *J* = 11.8 Hz, 1H), 2.79–2.71 (m, 1H), 1.75–1.63 (d, *J* = 11.2 Hz, 2H), 1.60–1.50 (m, 2H), 1.47–1.36 (m, 1H), 1.18 (d, *J* = 10.2 Hz, 1H), 1.01 (t, *J* = 13.0 Hz, 1H), 0.80–0.74 (m, 4H). ^13^C NMR (125 MHz, DMSO-*d_6_*): δ = 172.3, 139.0, 131.8, 130.1, 128.9, 128.6, 128.2, 127.5, 126.9, 64.0, 50.1, 45.3, 44.3, 42.9, 41.9, 41.3, 34.3, 25.1, 24.4, 22.2. HRMS (ESI) *m*/*z*: calcd for C_24_H_33_N_2_O_2_ [M+H]^+^: 381.2542; found: 381.2530.

*(R)-N-Benzyl-3-(benzylthio)-2-((1S,2S,4R)-2-hydroxy-4-methylcyclohexyl)propanamide (***37***)*: Chromatographed by *n*-hexane/EtOAc 2:1. Yield: 54%; white powder, mp. 172.1–175.4 °C; $\left[\alpha \right]\begin{array}{c} 20\\ D \end{array}$ = +84.00 (*c* = 0.17, MeOH). ^1^H NMR (500 MHz, CDCl_3_): δ = 7.38–7.16 (m, 10H), 5.77 (t, *J* = 4.8 Hz, 1H), 4.48 (dd, *J* = 14.7, 5.7 Hz, 1H), 4.39 (dd, *J* = 14.7, 5.5 Hz, 1H), 3.94 (s, 1H), 3.74–3.64 (m, 2H), 2.74 (d, *J* = 7.2 Hz, 2H), 2.20 (dd, *J* = 15.9, 7.2 Hz, 1H), 1.78 (ddd, *J* = 13.8, 5.7, 3.2 Hz, 1H), 1.72–1.58 (m, 3H), 1.44–1.37 (m, 1H), 1.36–1.27 (m, 1H), 1.18–1.06 (m, 1H), 0.93–0.82 (m, 4H). ^13^C NMR (125 MHz, CDCl_3_): δ = 174.0, 138.9, 138.3, 129.1, 128.8, 128.7, 128.2, 127.6, 127.3, 67.2, 50.3, 43.8, 43.5, 42.5, 37.4, 34.6, 32.2, 25.9, 24.8, 22.3. HRMS (ESI) *m*/*z*: calcd for C_24_H_32_NO_2_S [M+H]^+^: 398.2154; found: 398.2144.

*(S)-3-(1H-Benzo[d]imidazol-1-yl)-N-benzyl-2-((1S,2S,4R)-2-hydroxy-4-methylcyclo-hexyl)propanamide (***50a***)*: Chromatographed by CHCl_3_/*t*-BuOH 19:1. Yield: 39%; white powder, mp. 181.0–184.2 °C; $\left[\alpha \right]\begin{array}{c} 20\\ D \end{array}$ = −52.53 (*c* = 0.23, MeOH). ^1^H NMR (500 MHz, CDCl_3_): δ = 7.86 (s, 1H), 7.84–7.77 (m, 1H), 7.42–7.36 (m, 1H), 7.28 (d, *J* = 4.5 Hz, 2H), 7.18 (d, *J* = 3.3 Hz, 3H), 6.89–6.82 (m, 2H), 5.78 (s, 1H), 4.65 (dd, *J* = 13.5, 11.9 Hz, 1H), 4.35 (dd, *J* = 14.0, 3.1 Hz, 1H), 4.26 (dd, *J* = 14.7, 5.6 Hz, 1H), 4.15 (dd, *J* = 14.7, 5.6 Hz, 1H), 3.96 (s, 1H), 2.80–2.70 (m, 1H), 2.05 (s, 1H), 1.86–1.73 (m, 5H), 1.72–1.60 (m, 1H), 1.19 (t, *J* = 13.6 Hz, 1H), 1.06–0.95 (m, 1H), 0.91 (d, *J* = 6.1 Hz, 3H). ^13^C NMR (125 MHz, CDCl_3_): δ = 173.0, 144.0, 143.9, 137.4, 133.6, 128.8, 127.7, 127.6, 123.2, 122.4, 120.7, 109.6, 67.6, 51.3, 45.6, 43.9, 42.4, 42.5, 34.7, 25.8, 24.8, 22.2. HRMS (ESI) *m*/*z*: calcd for C_24_H_30_N_3_O_2_ [M+H]^+^: 392.2338; found: 392.2326.

*(R)-3-(1H-Benzo[d]imidazol-1-yl)-N-benzyl-2-((1S,2S,4R)-2-hydroxy-4-methylcyclo-hexyl)propanamide (***50b***)*: Chromatographed by CHCl_3_/*t*-BuOH 19:1. Yield: 60%; white powder, mp. 258.2–260.2 °C; $\left[\alpha \right]\begin{array}{c} 20\\ D \end{array}$ = +41.23 (*c* = 0.21, MeOH). ^1^H NMR (500 MHz, CD_3_OD): δ = 7.97 (s, 1H), 7.69 (d, *J* = 7.5 Hz, 1H), 7.65 (d, *J* = 7.8 Hz, 1H), 7.34–7.26 (m, 2H), 7.14–7.06 (m, 3H), 6.71–6.66 (m, 2H), 4.60 (dd, *J* = 13.8, 3.9 Hz, 1H), 4.50 (t, *J* = 12.7 Hz, 1H), 4.26–4.15 (m, 2H), 3.95 (d, *J* = 14.9 Hz, 1H), 3.01 (td, *J* = 11.0, 3.9 Hz, 1H), 1.93 (dd, *J* = 13.8, 1.9 Hz, 1H), 1.88–1.80 (m, 1H), 1.80–1.72 (m, 1H), 1.69 (d, *J* = 12.9 Hz, 1H), 1.50 (qd, *J* = 13.0, 3.2 Hz, 1H), 1.37 (dd, *J* = 13.0, 3.2 Hz, 1H), 1.39–1.21 (m, 1H), 1.00–0.88 (m, 4H). ^13^C NMR (125 MHz, CD_3_OD): δ = 175.4, 145.0, 144.0, 139.1, 134.9, 129.3, 128.2, 128.0, 124.3, 123.5, 120.1, 111.9, 66.9, 50.8, 46.0, 43.9, 43.6, 42.9, 35.6, 26.9, 26.4, 22.7. HRMS (ESI) *m*/*z*: calcd for C_24_H_30_N_3_O_2_ [M+H]^+^: 392.2338; found: 392.2327.

### 3.9. Determination of the Antimicrobial Effect

#### 3.9.1. Reagents and Media

DMSO (Sigma-Aldrich, St Louis, MO, USA), phosphate-buffered saline (PBS; pH 7.4), Mueller–Hinton (MH) broth, tryptic soy broth (TSB), tryptic soy agar (TSA), Luria–Bertani broth (LBB), and Luria–Bertani agar (LBA) were used. All reagents were purchased from Sigma.

#### 3.9.2. Bacterial Strains

*Escherichia coli* ATCC 25922 was used as the Gram-negative strain. Gram-positive strains investigated in this study included the following: *Staphylococcus aureus* American Type Culture Collection (ATCC) 25923 as a methicillin-susceptible reference strain, and the methicillin- and oxacillin-resistant *S. aureus* MRSA ATCC 43300 strain.

#### 3.9.3. Antibacterial Activity

The minimum inhibitory concentrations (MICs) of the compounds were determined according to the Clinical and Laboratory Standard Institute guidelines [41]. Two-fold serial dilutions of the compounds were prepared in Mueller–Hinton broth in 96-well plates, and the starting concentration of the compounds was 100 µM (Sigma-Aldrich, St. Louis, MO, USA). The MIC values of the compounds were determined by visual inspection. DMSO as a solvent was also assayed to ensure that it had no antibacterial effect.

### 3.10. Computational Studies

The crystal structures of the protein templates were obtained from PDB (protein data bank), and the tested structures were drawn by ChemBioDraw Ultra 12.0. The Accelrys Discovery Studio 3.5 software was used to perform the docking study and the in silico ADMET prediction.

#### 3.10.1. Preparation of the Crystal Structures of the Protein Targets

The structures were prepared by using the Accelrys Discovery Studio 3.5 software’s Clean Geometry option after eliminating the water molecules. Then, the absent hydrogen atoms were furnished by applying the CHARMm force field. Adopted Basis minimization was utilized to minimize the complex energy and to get the most stable structure without altering the basic protein skeleton. Thereafter, a 10 Å radius sphere was created to define the active site residues [46].

#### 3.10.2. Docking Study (CDOCKER)

The CDOCKER method enables the generation of all possible conformations of the compound within the protein’s active site. These conformations were then assessed according to both the CDOCKER energy and the interactions observed between the ligand and the active site. This approach requires preparing the crystal structure (as previously described) and the examined compound, which involves using the Accelrys Discovery Studio protocol and applying a force field. Before initiating this study, it is crucial to validate the method by comparing the conformation of the reference compound to the conformations generated by the docking approach, ensuring that the RMSD (Root Mean Square Deviation) remains within the acceptable range.

#### 3.10.3. In Silico ADMET prediction

The ADMET properties of compounds **28** and **30** were predicted using ADMET descriptors in the Accelrys Discovery Studio 3.5 software. Multiple mathematical models were employed for quantitative prediction. These models included the following: aqueous solubility (predict solubility in water at 25 °C), blood–brain barrier (BBB) penetration, cytochrome P450 (CYP2D6) inhibition, human intestinal absorption (HIA), and plasma protein binding (PPB) [47]. An ADMET model was also generated to predict the human intestinal absorption (HIA) and blood–brain barrier (BBB) penetration of the tested compounds. The model shows the ADMET_PSA_2D and ADMET_ALogP98 plot with 95 and 99% confidence limit ellipses (Figure S87 in Supplementary Materials).

## 4. Conclusions

In summary, starting from the commercially available (−)-isopulegol, a new family of Michael-type amino and thiol adducts were prepared and characterized through chiral (+)-neoisopulegol as a key intermediate via stereoselective transformations.

The resulting *β*-aminolactones exert selective inhibition for the Gram-positive bacteria *S. aureus*. In vitro pharmacological studies have clearly shown that the *N*-naphthylmethyl substituent on the *β*-aminolactone function is essential.

Finally, molecular docking results exemplified that the *β*-aminolactones **28** and **30** could foster potent affinity by forming significant interactions with DNA gyrase (PDB code: 3U2D) and C(30) carotenoid dehydrosqualene synthase (PDB code: 2ZCQ). Hence, *N*-naphthylmethyl-substituted *β*-aminolactone has the potential to be developed into clinically important therapeutic choices for the treatment of infections caused by *S. aureus*.

While the thiol and azole Michael adducts showed no notable antimicrobial activity in our assays, their structurally diverse and heteroatom-rich frameworks suggest potential for alternative bioactivities. This motivates further pharmacological screening, especially given prior reports of such scaffolds in biomedical research.

## Figures and Tables

**Table 1:** Nucleophilic addition of (−)-**20** with benzylamines.

Entry	Compound	R	Yield (%)
1	**23**	Benzyl	89
2	**24**	*o*-Fluorobenzyl	42
3	**25**	*m*-Fluorobenzyl	39
4	**26**	*p*-Fluorobenzyl	52
5	**27**	*o*-Methoxybenzyl	48
6	**28**	*m*-Methoxybenzyl	66
7	**29**	*p*-Methoxybenzyl	69
8	**30**	1-Naphthylmethyl	90

**Table 2:** Nucleophilic addition of (–)-**20** with benzyl mercaptans.

Entry	Compound	R	Yield (%)
1	**33**	H	80
2	**34**	F	76
3	**35**	OCH_3_	73

**Table 3:** Nucleophilic addition of (−)-**20** with cysteine derivatives.

Entry	Compound	R^1^	R^2^	Yield (%)
1	**38**	OH	COCH_3_	71
2	**39**	OCH_3_	H	50
3	**40**	OCH_3_	COCH_3_	62
4	**41**	NH_2_	COCH_3_	41
5	**42**	OC_2_H_5_	COCH_3_	93
6	**43**	OCH_3_	COO*^t^*Bu	54

**Table 4:** Nucleophilic addition of (–)-**20** with azoles.

Entry	Compound	R	Yield (%)	
			(a)	(b)
1	**44**	Imidazol-1-yl	50	14
2	**45**	1,2,4-Triazol-1-yl	57	18
3	**46**	1,2,3-Triazol-1-yl	53	28
4	**47**	Benzimidazol-1-yl	20	12
5	**48**	Benzotriazol-1-yl	21	12

**Table 5:** Antibacterial activity of *β*-aminolactones on *S. aureus* ATCC 25923. Results of the antimicrobial assay of other compounds are provided in Table S1 in Supplementary Materials.

Entry	Compound	MIC (µM) on the Following Bacteria:		
		*S. aureus* ATCC 25923	*S. aureus* MRSA ATCC 43300	*E. coli* ATCC 25922
1	**23**	100	>100	>100
2	**24**	100	>100	>100
3	**25**	100	>100	>100
4	**26**	100	>100	>100
5	**27**	100	>100	>100
6	**28**	**50**	>100	>100
7	**29**	100	>100	>100
8	**30**	**12.5**	>100	>100
9	**Ciprofloxacin**	4.2	4.2	2.1
10	**Tetracycline**	0.6	0.6	0.4

## Data Availability

The original contributions presented in this study are included in the article and Supplementary Materials. Further inquiries can be directed to the corresponding author.

## Supplementary Materials

The following supporting information can be downloaded at https://www.mdpi.com/article/10.3390/ijms26104791/s1.
