# Supplementary material for: Synthesis and Antimicrobial Evaluation of (+)-Neoisopulegol-Based Amino and Thiol Adducts
Source: Int J Mol Sci. 2025 May 16;26(10):4791. doi: 10.3390/ijms26104791 (PMC12111876; doi:10.3390/ijms26104791)
Supplement: Supplementary file 1 [file ijms-26-04791-s001.zip › ijms-3557973-supplementary.pdf]

Supporting information for

Synthesis and antimicrobial evaluation of (+)-neoisopulegol-based amino and thiol adducts

Reem Moustafa,[a] Attila Márió Remete,[a] Szakonyi Zsolt[a], Nikoletta Szemerédi,[b] Gabriella Spengler,[b] and Tam Minh Le*[,a,c]

[a] Institute of Pharmaceutical Chemistry, University of Szeged, Eötvös u. 6, H-6720 Szeged, Hungary.

[b] Department of Medical Microbiology, Albert Szent-Györgyi Health Center and Albert Szent-Györgyi Medical School, University of Szeged, Semmelweis utca 6, 6725, Szeged, Hungary.

[c] HUN-REN–SZTE Stereochemistry Research Group, University of Szeged, Eötvös u. 6, H-6720 Szeged, Hungary.

Contents

1. **NMR spectra of new compounds** **S3–S50**
2. **Antimicrobial assay (results of all products)** **S51–52**
3. **Molecular docking** **S53**

1. NMR spectra of new compounds

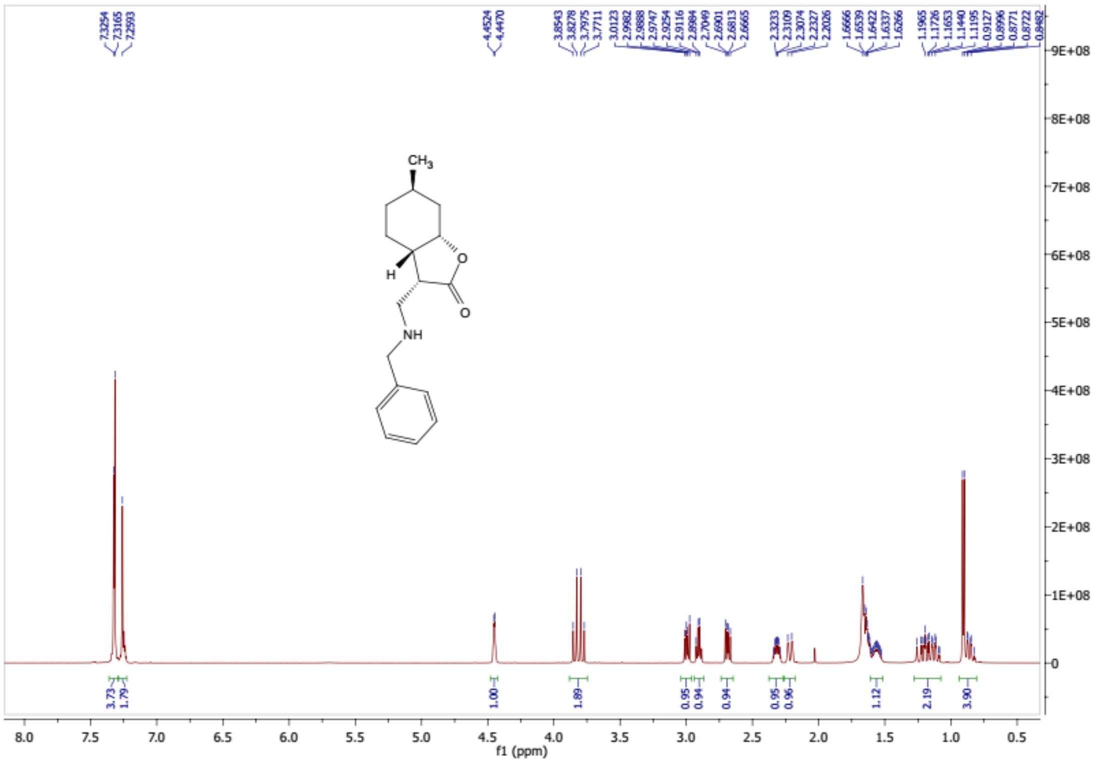

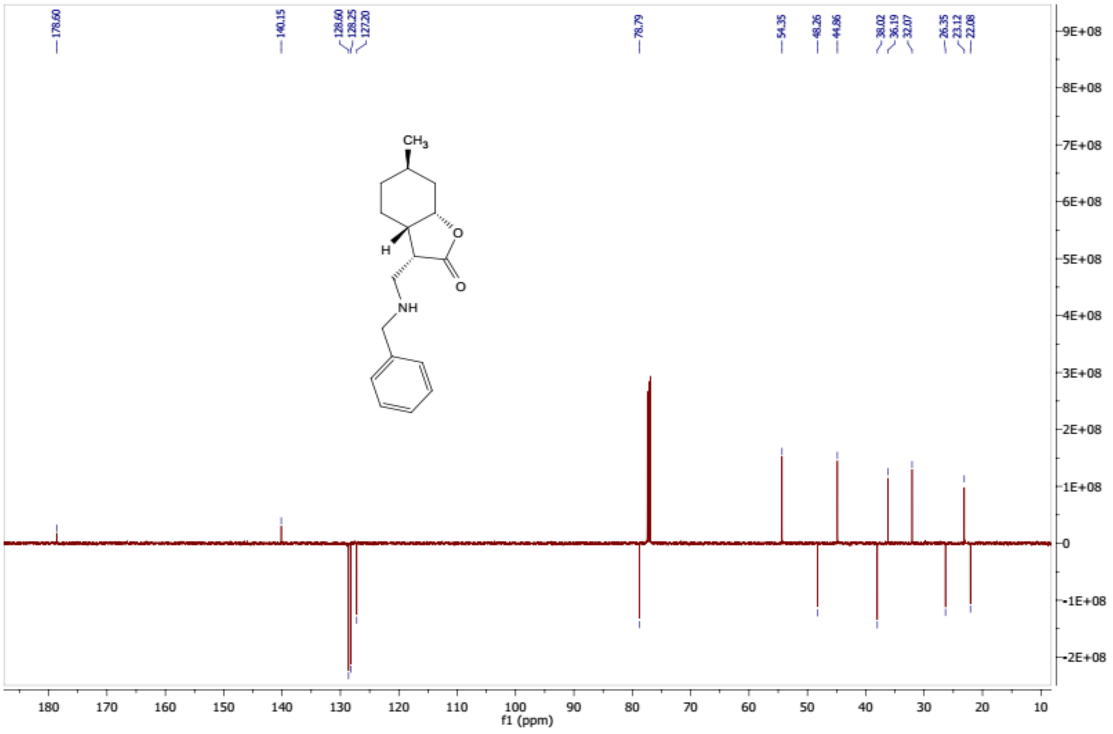

Figures S1-S2. ^{1}H (500 MHz, $CDCl_3$) and ^{13}C (125 MHz, $CDCl_3$) NMR spectra of **23.**

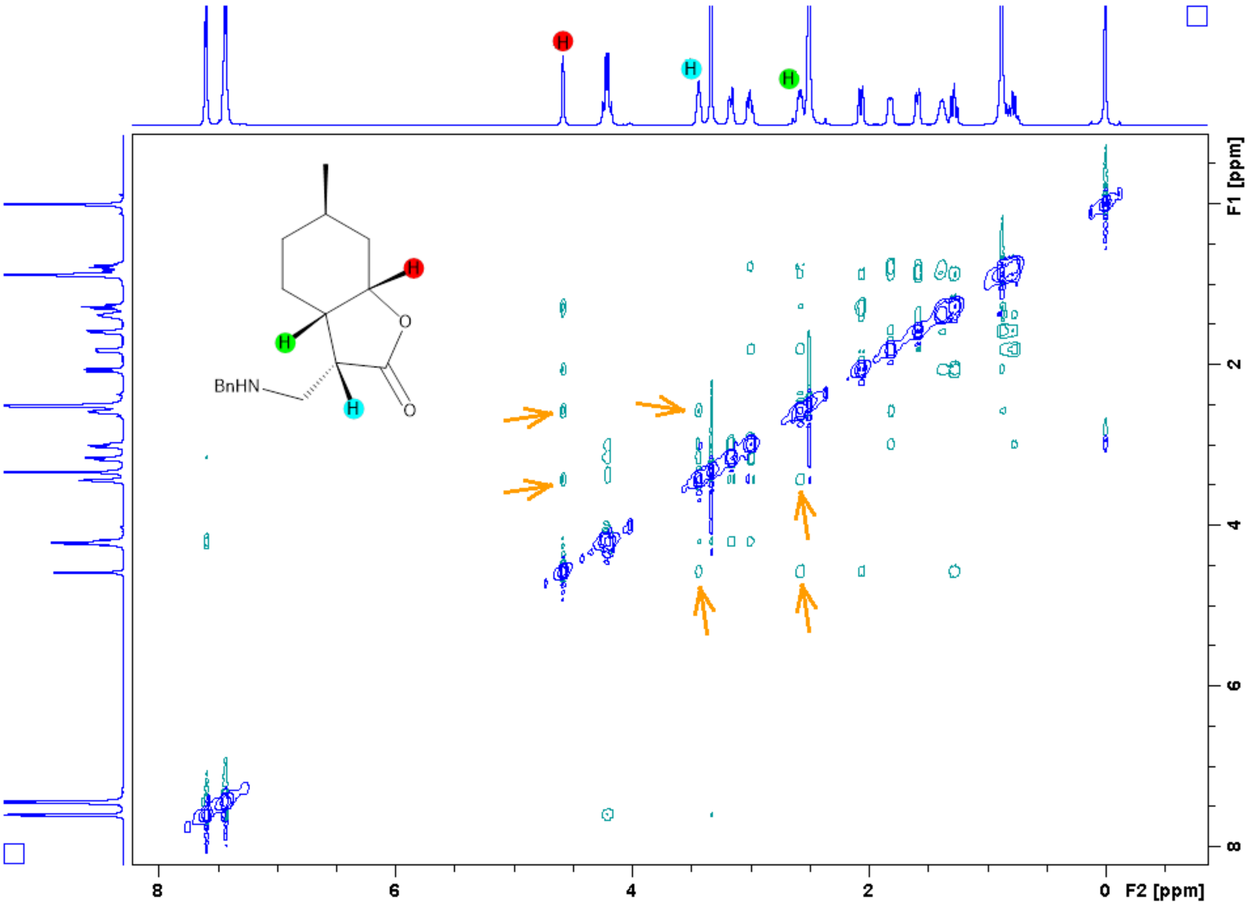

Figure S3. NOESY (500 MHz, DMSO-d_6) spectra of **23.**

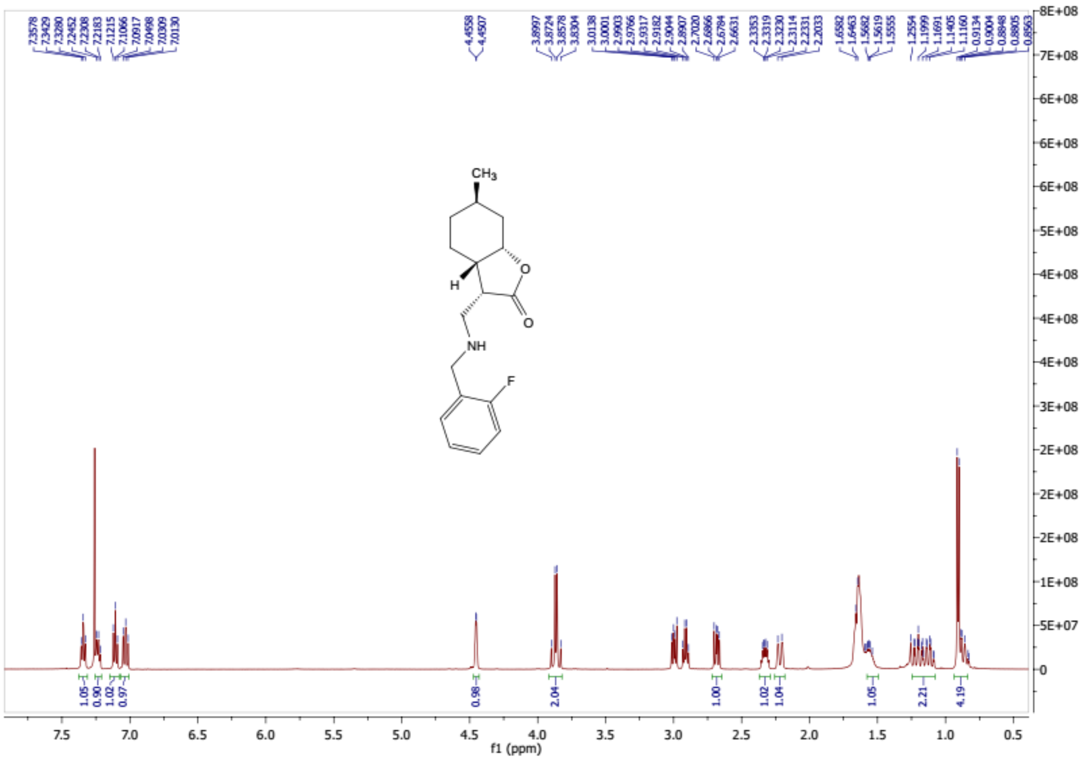

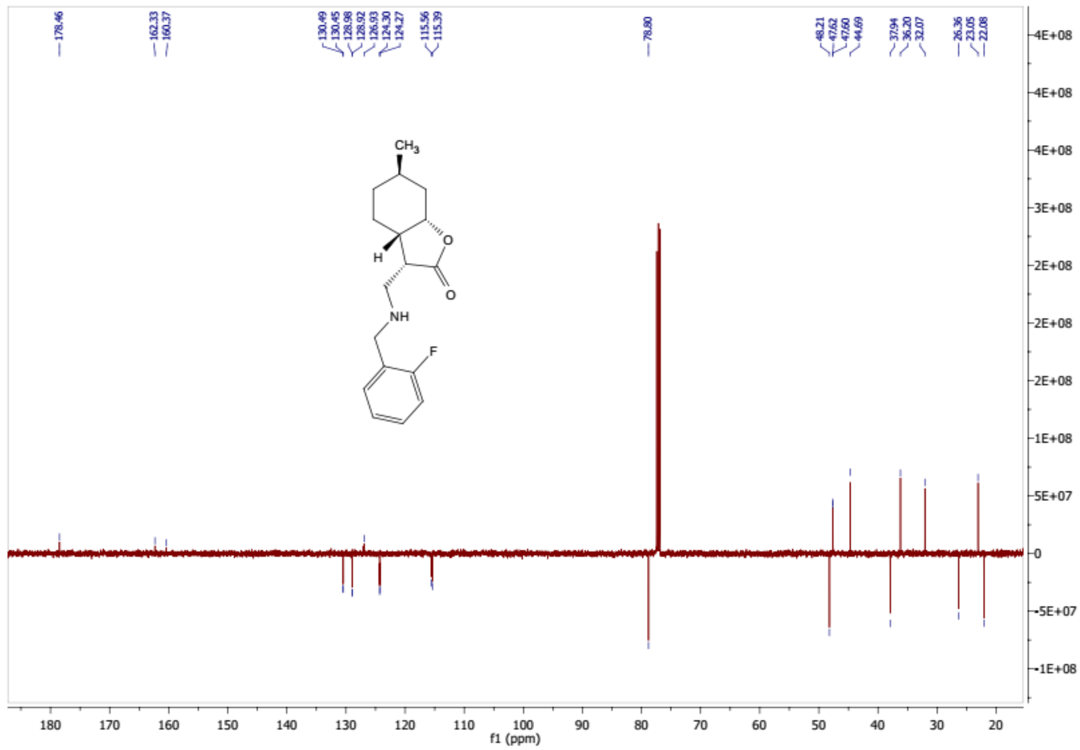

Figures S4-S5. ^{1}H (500 MHz, $CDCl_3$) and ^{13}C (125 MHz, $CDCl_3$) NMR spectra of **24.**

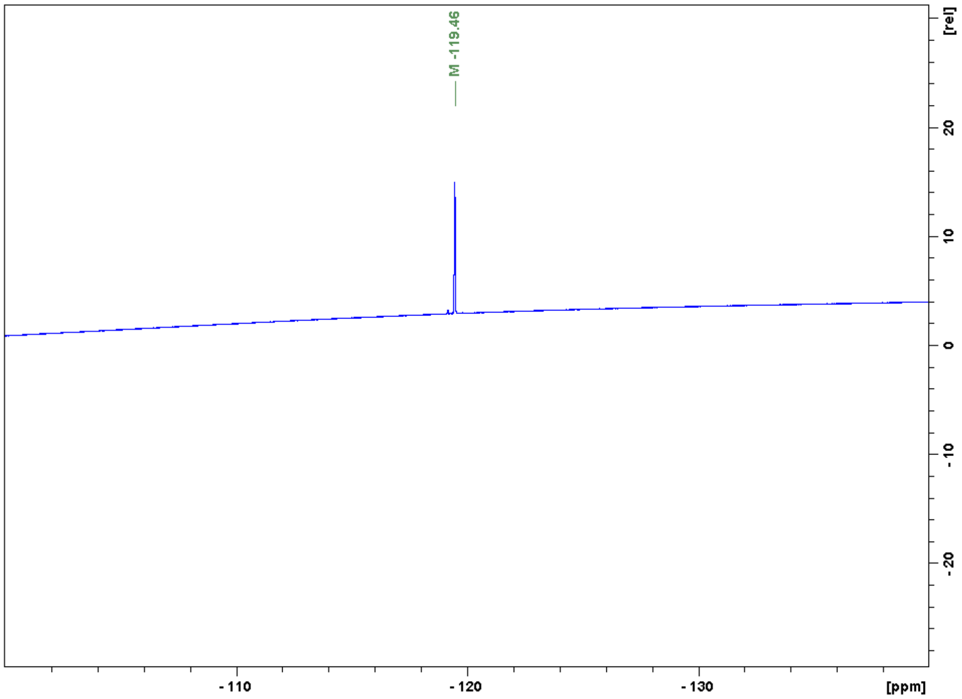

Figure S6. ^{19}F (471 MHz, $CDCl_3$) NMR spectra of **24.**

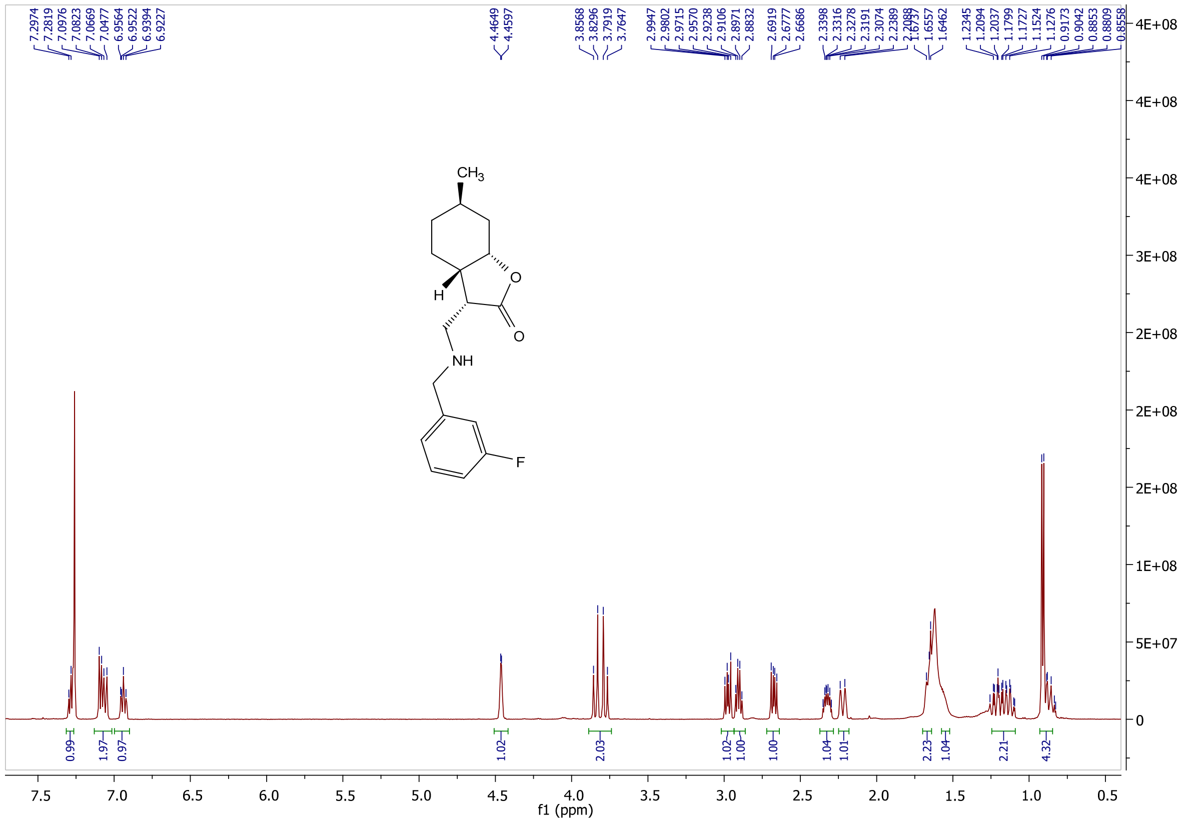

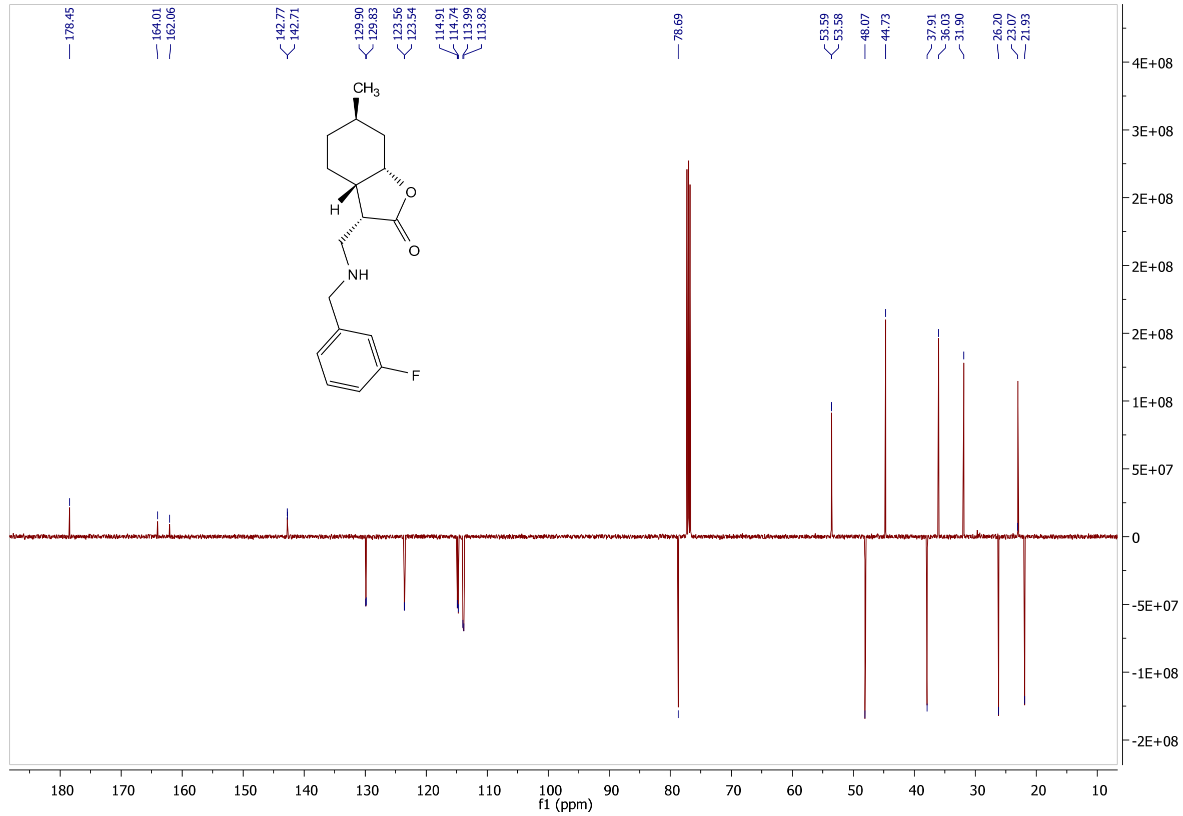

Figures S7-S8. ^{1}H (500 MHz, $CDCl_3$) and ^{13}C (125 MHz, $CDCl_3$) NMR spectra of **25.**

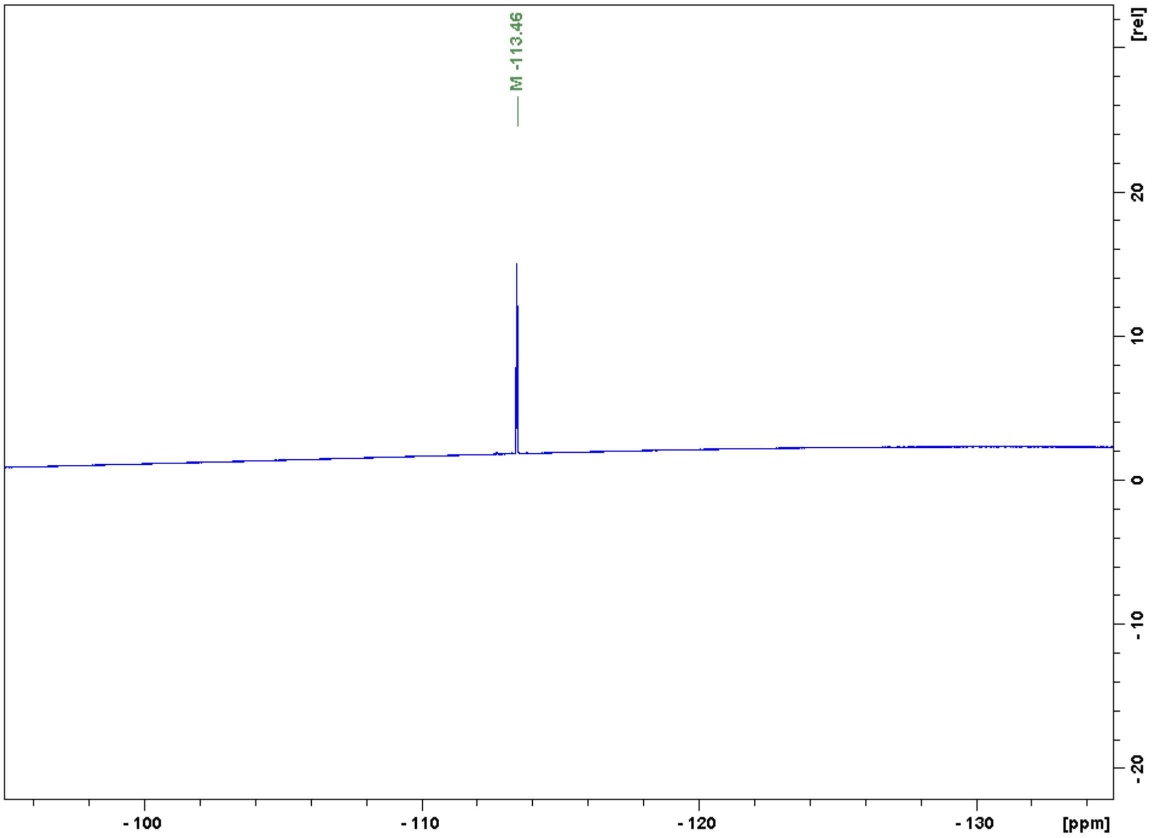

Figure S9. ^{19}F (471 MHz, $CDCl_3$) NMR spectra of **25.**

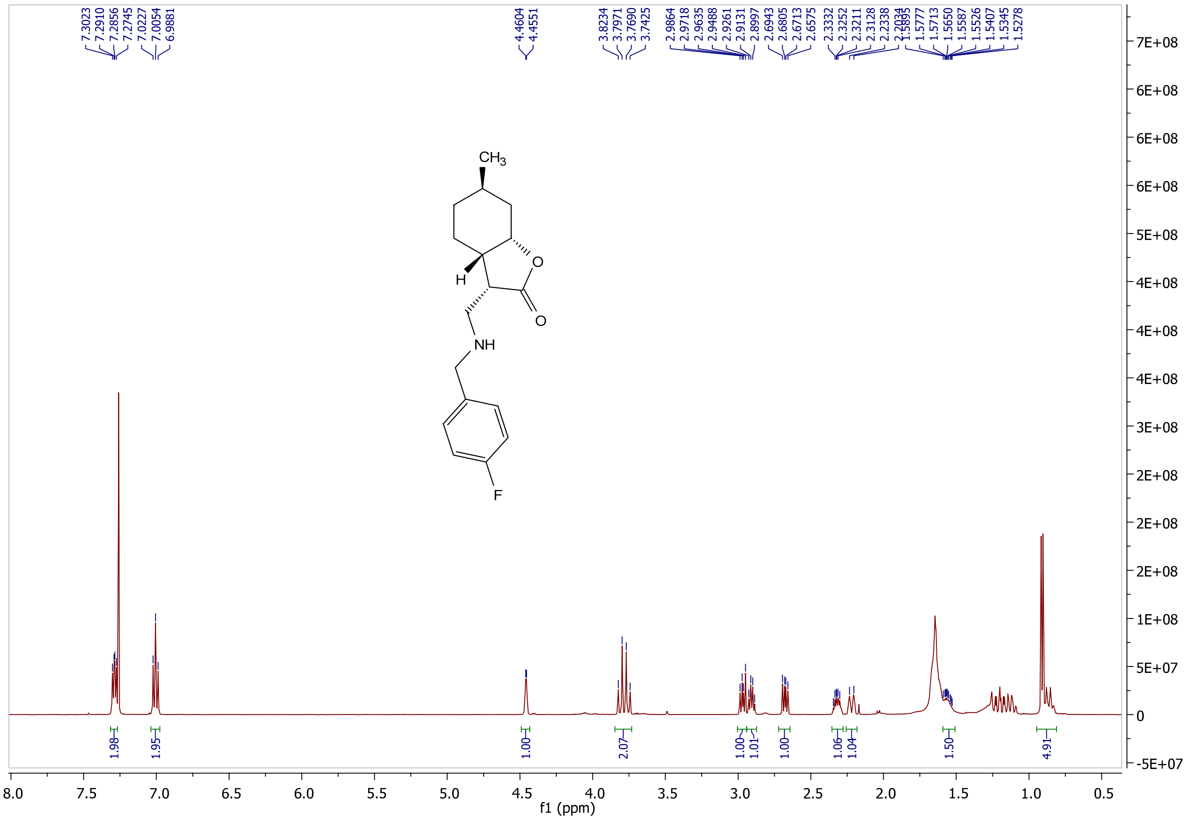

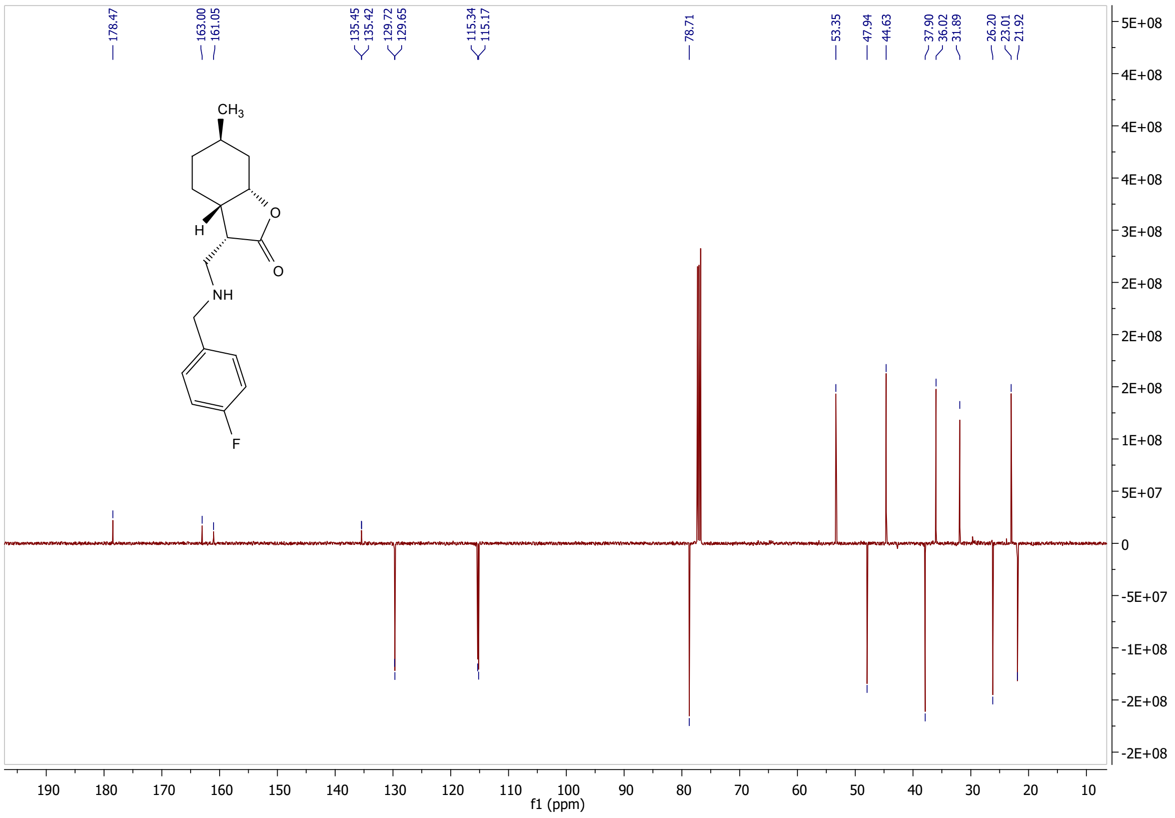

Figures S10-S11. ^{1}H (500 MHz, $CDCl_3$) and ^{13}C (125 MHz, $CDCl_3$) NMR spectra of **26.**

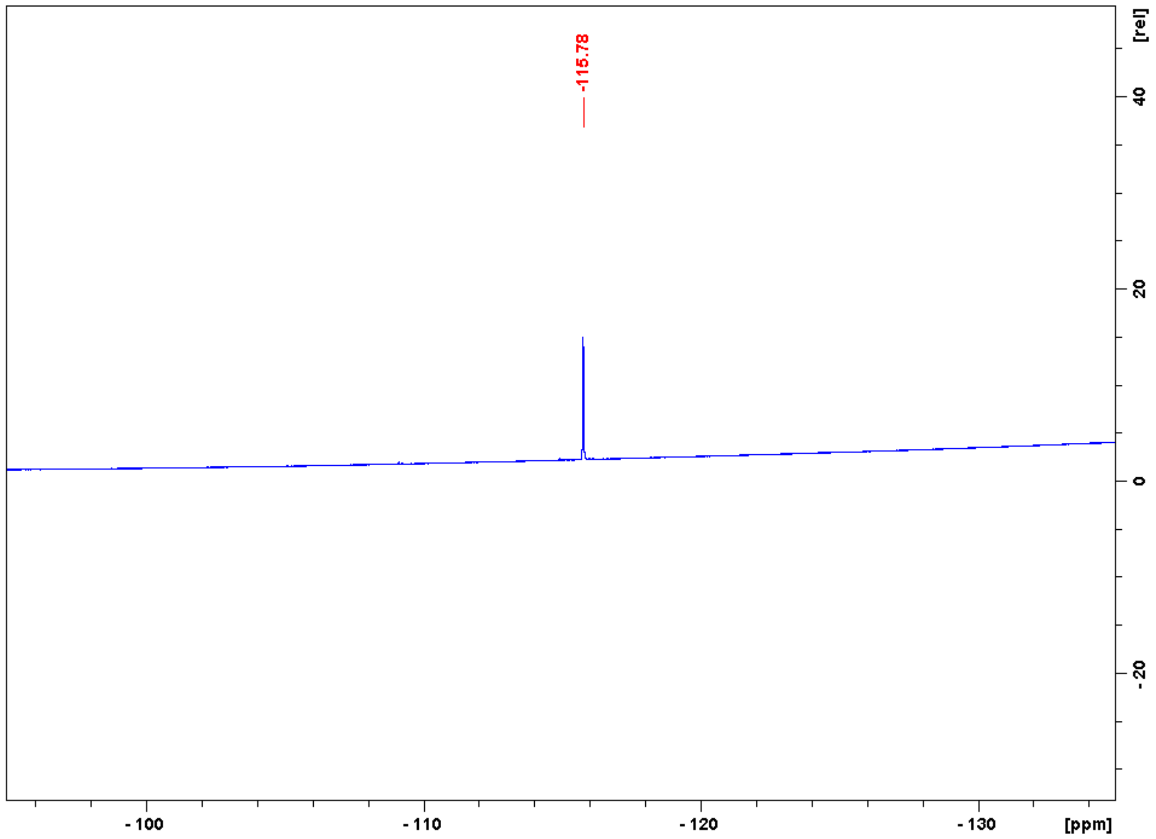

Figure S12. ^{19}F (471 MHz, $CDCl_3$) NMR spectra of **26.**

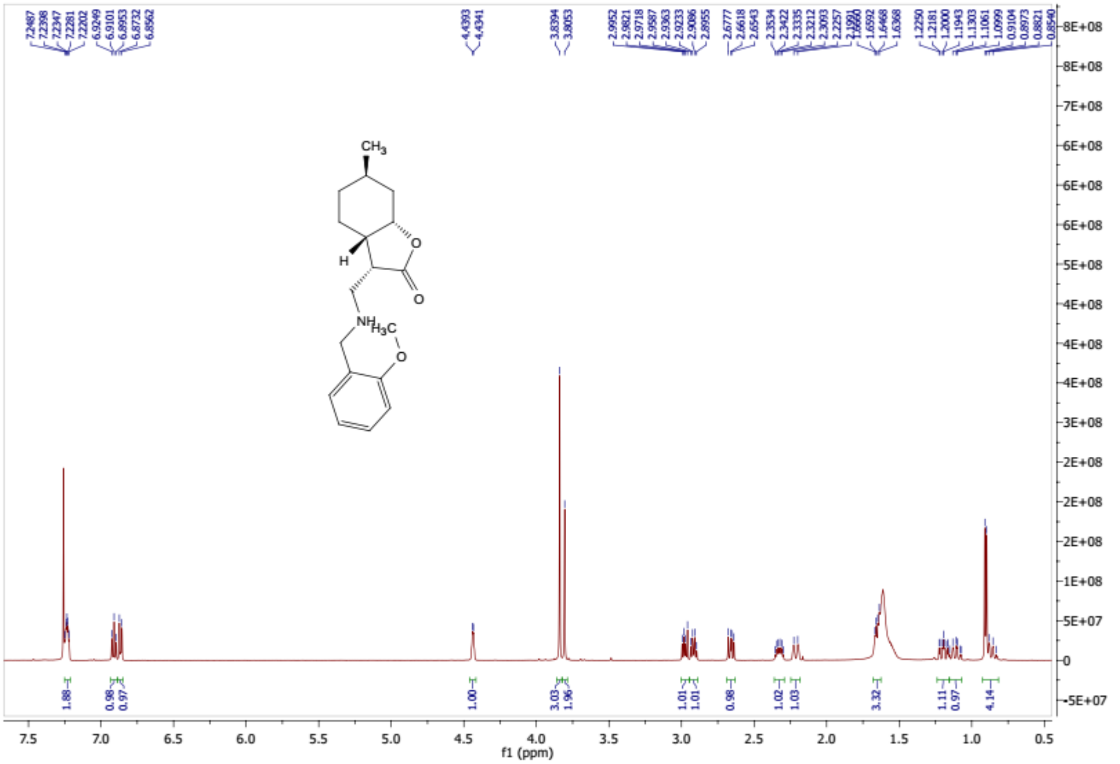

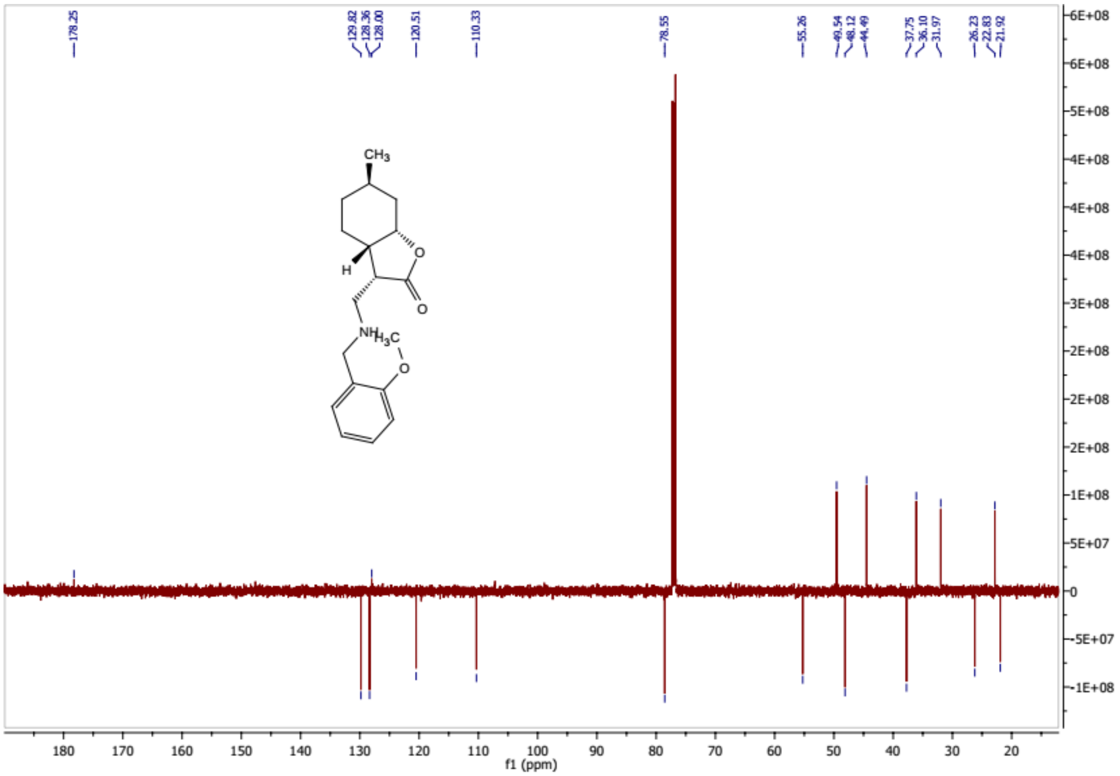

Figures S13-S14. ^{1}H (500 MHz, $CDCl_3$) and ^{13}C (125 MHz, $CDCl_3$) NMR spectra of **27.**

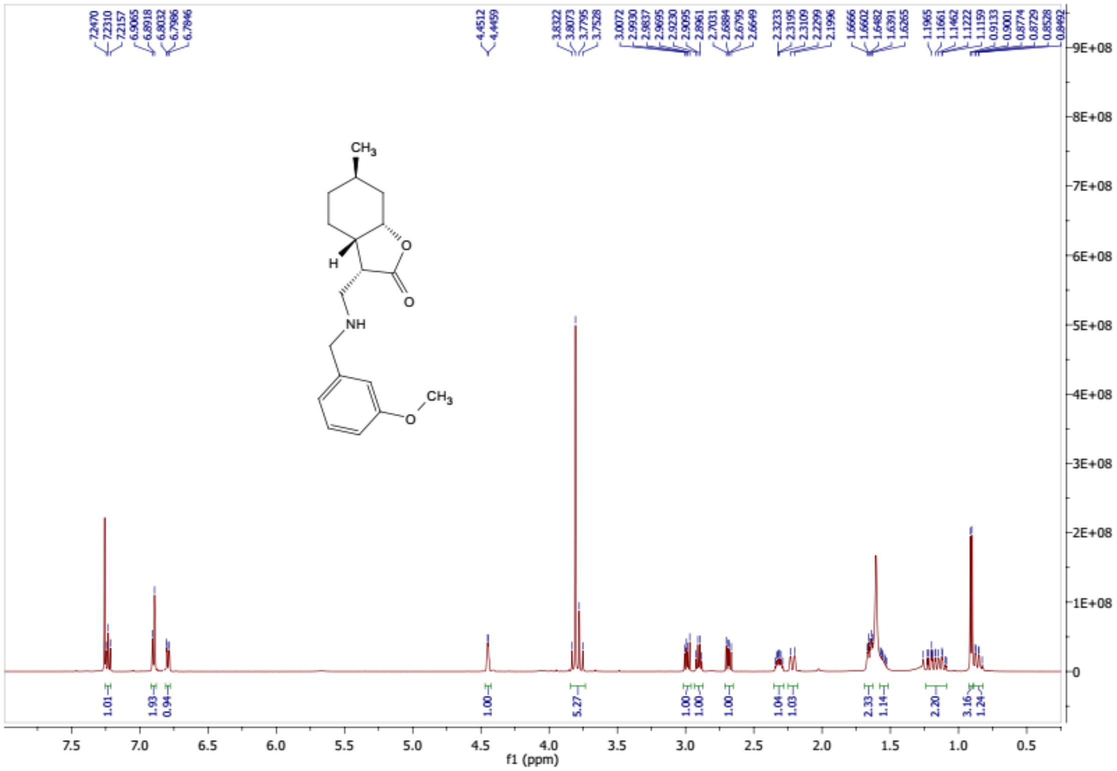

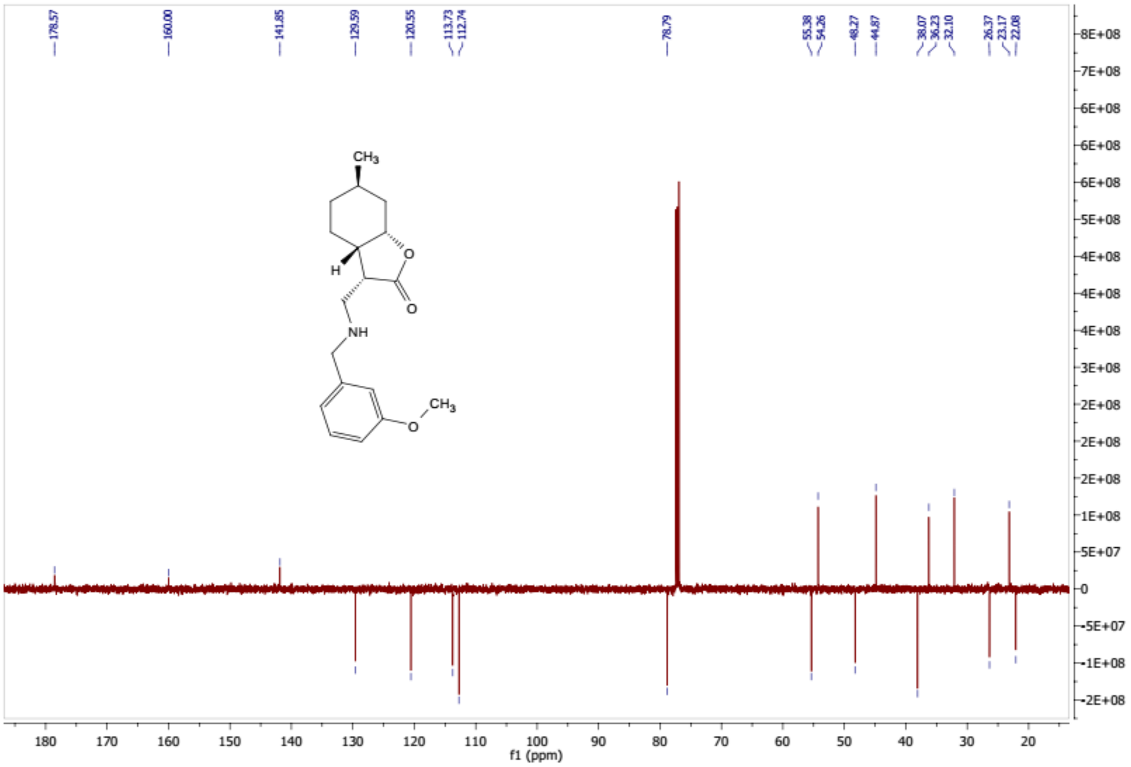

Figures S15-S16. ^{1}H (500 MHz, $CDCl_3$) and ^{13}C (125 MHz, $CDCl_3$) NMR spectra of **28.**

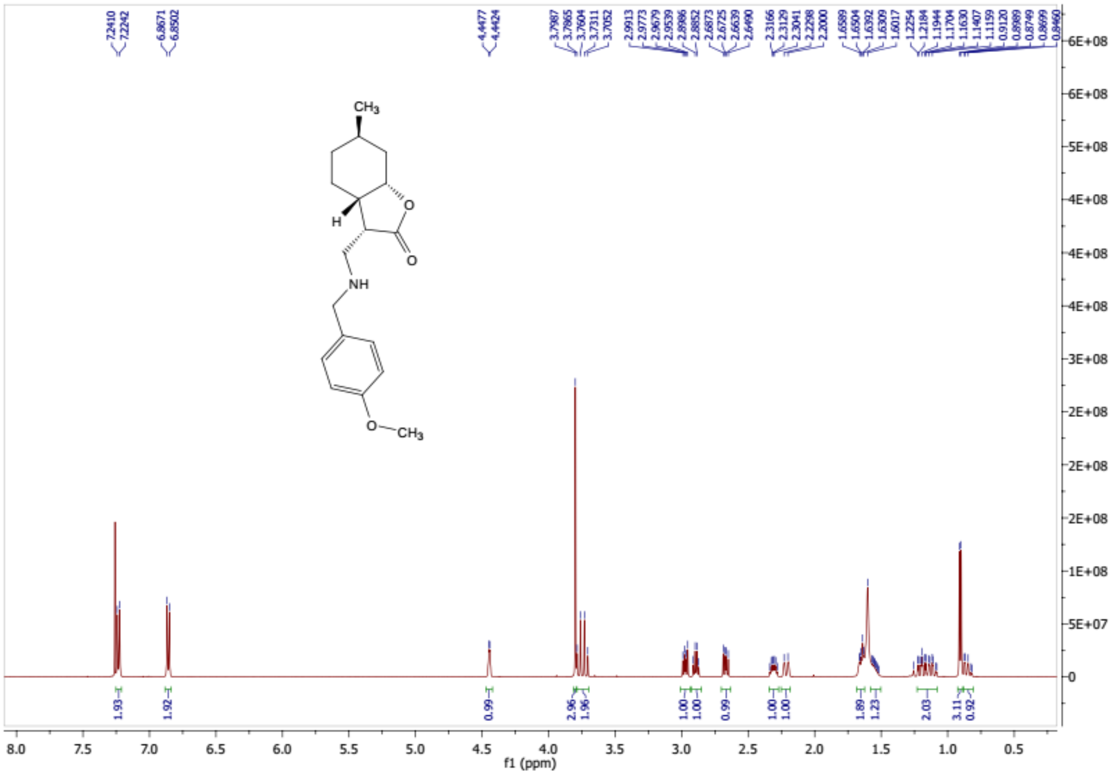

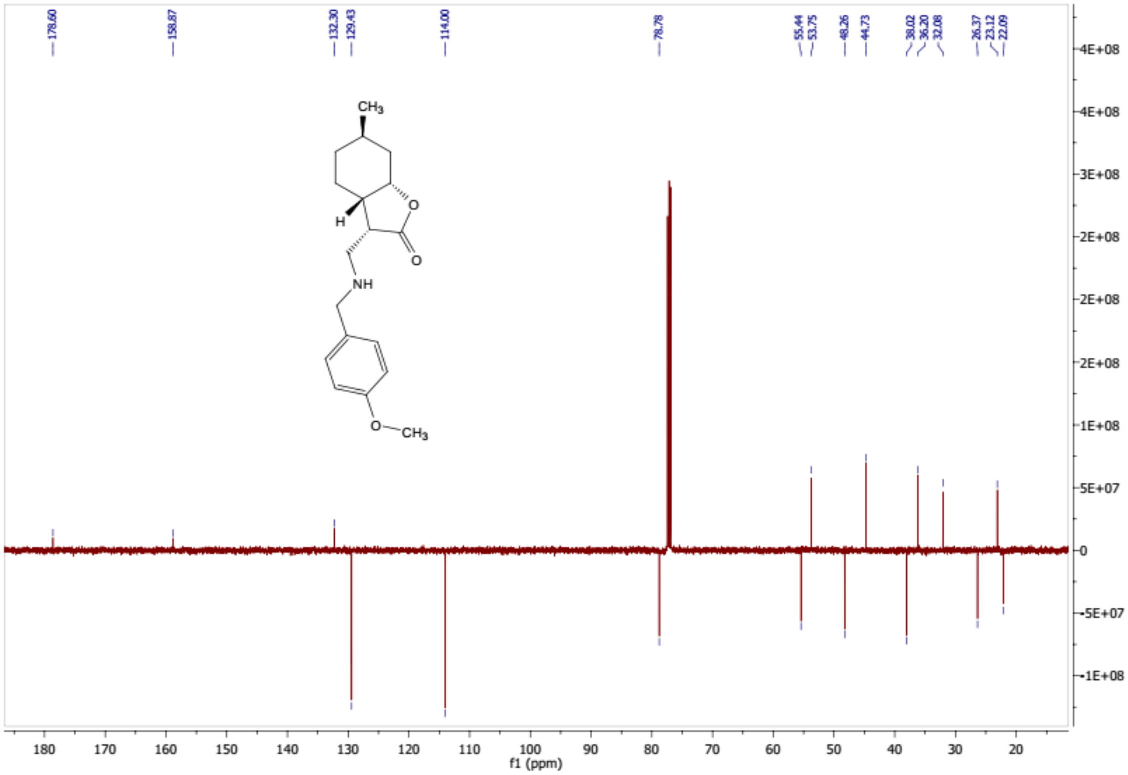

Figures S17-S18. ^{1}H (500 MHz, $CDCl_3$) and ^{13}C (125 MHz, $CDCl_3$) NMR spectra of **29.**

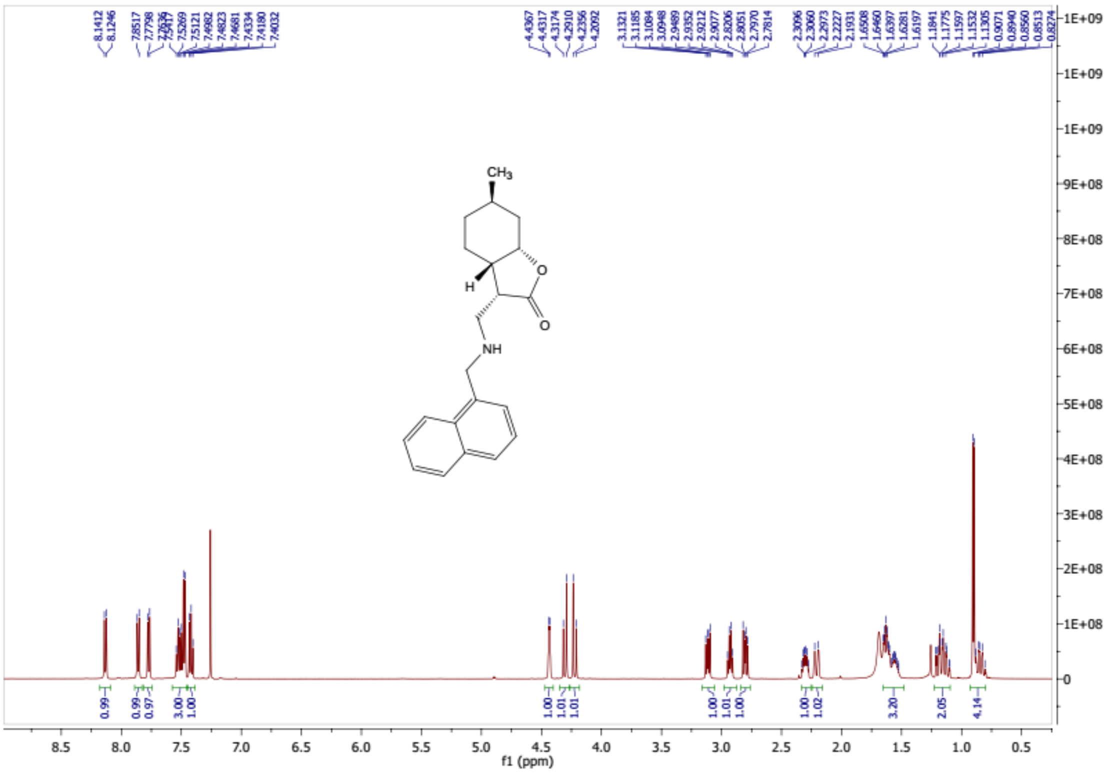

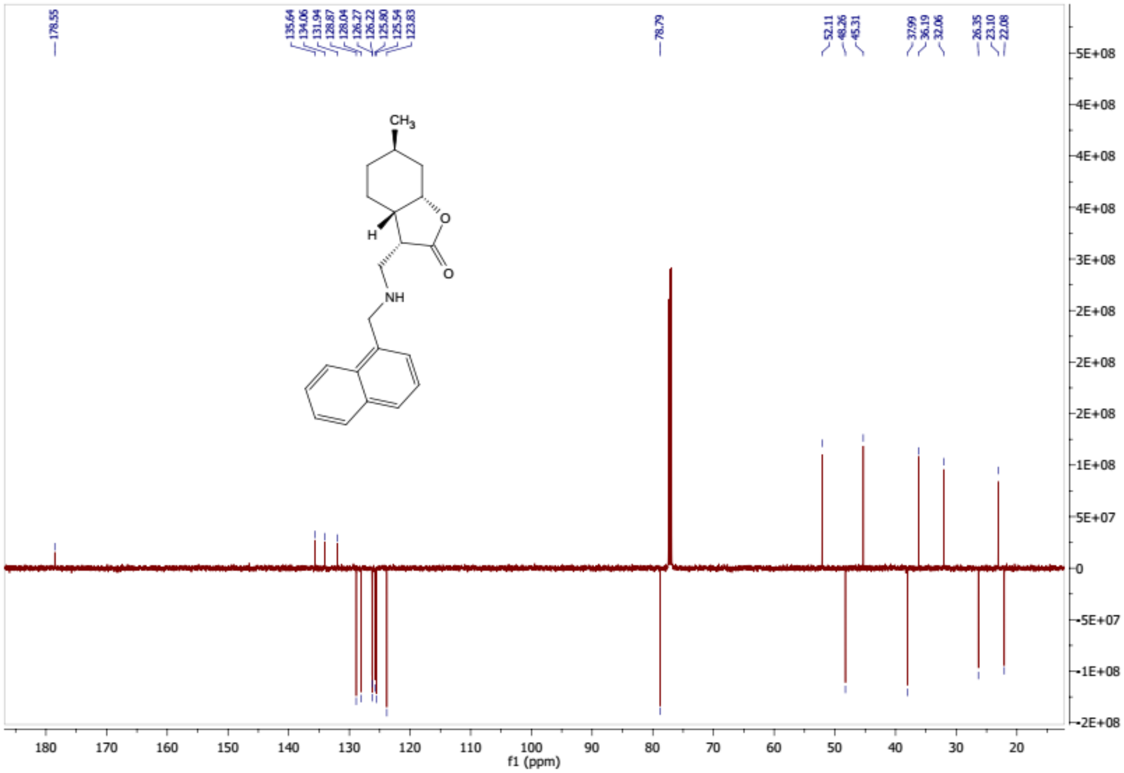

Figures S19-S20. ^{1}H (500 MHz, $CDCl_3$) and ^{13}C (125 MHz, $CDCl_3$) NMR spectra of **30.**

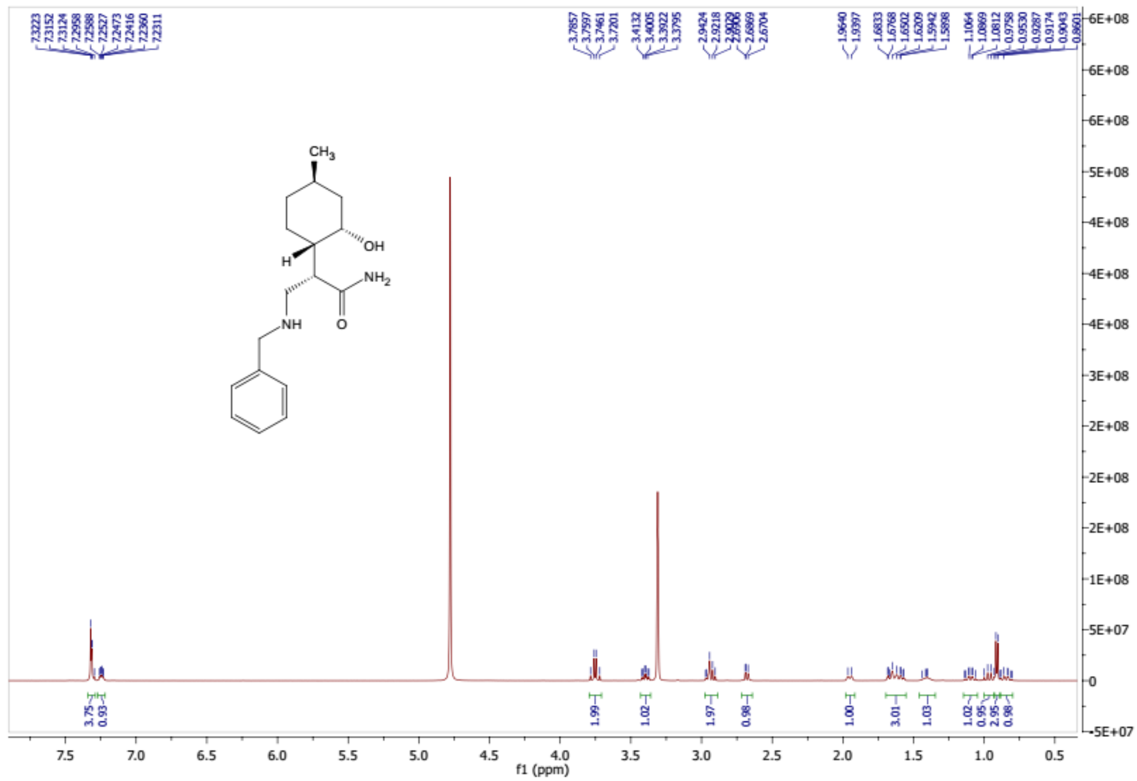

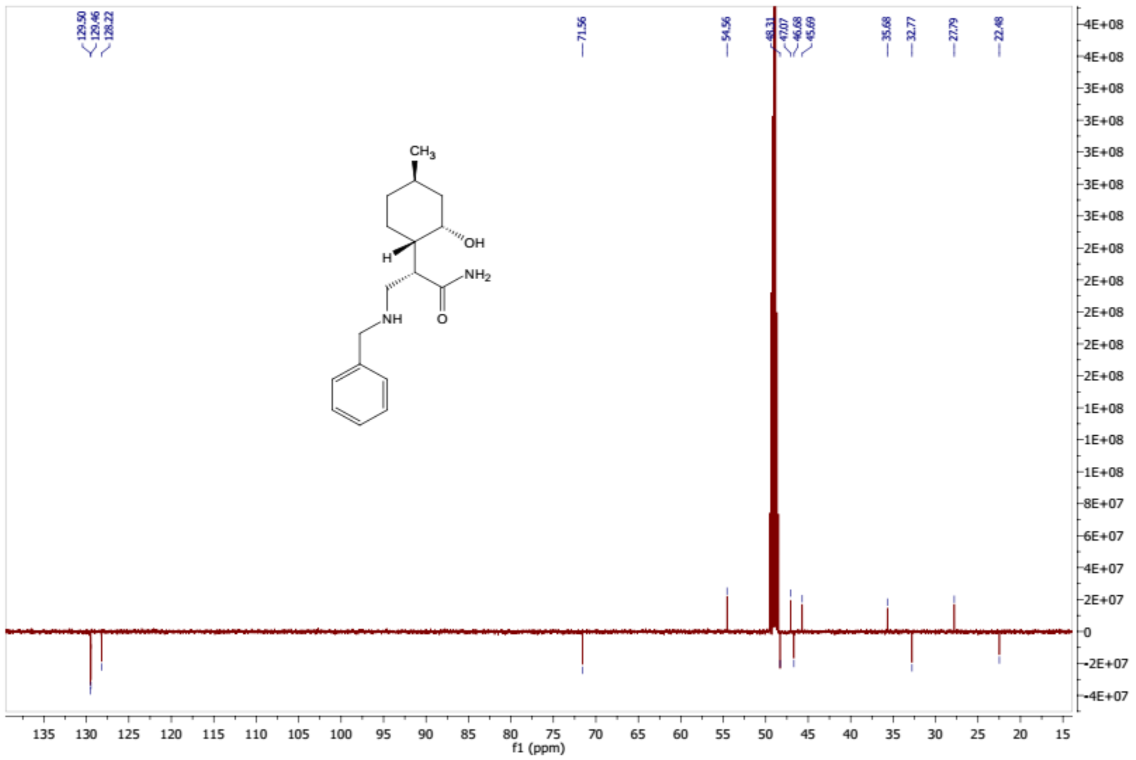

Figures S21-S22. ^{1}H (500 MHz, CD_3OD) and ^{13}C (125 MHz, CD_3OD) NMR spectra of **31.**

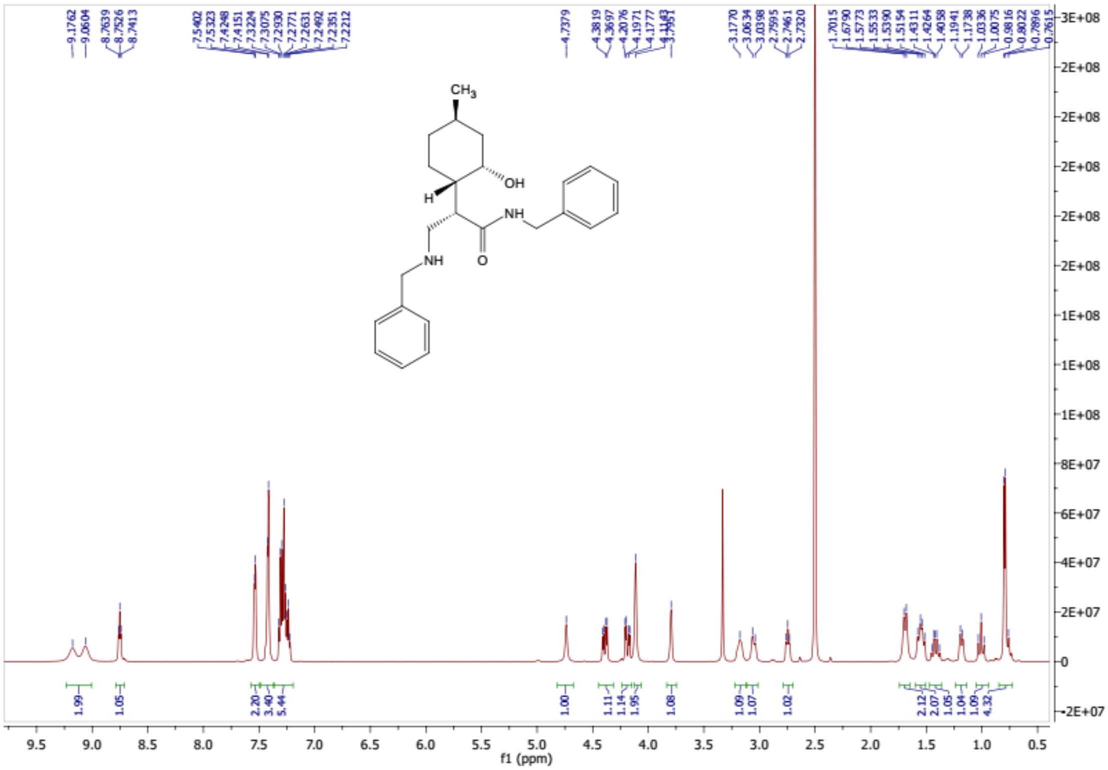

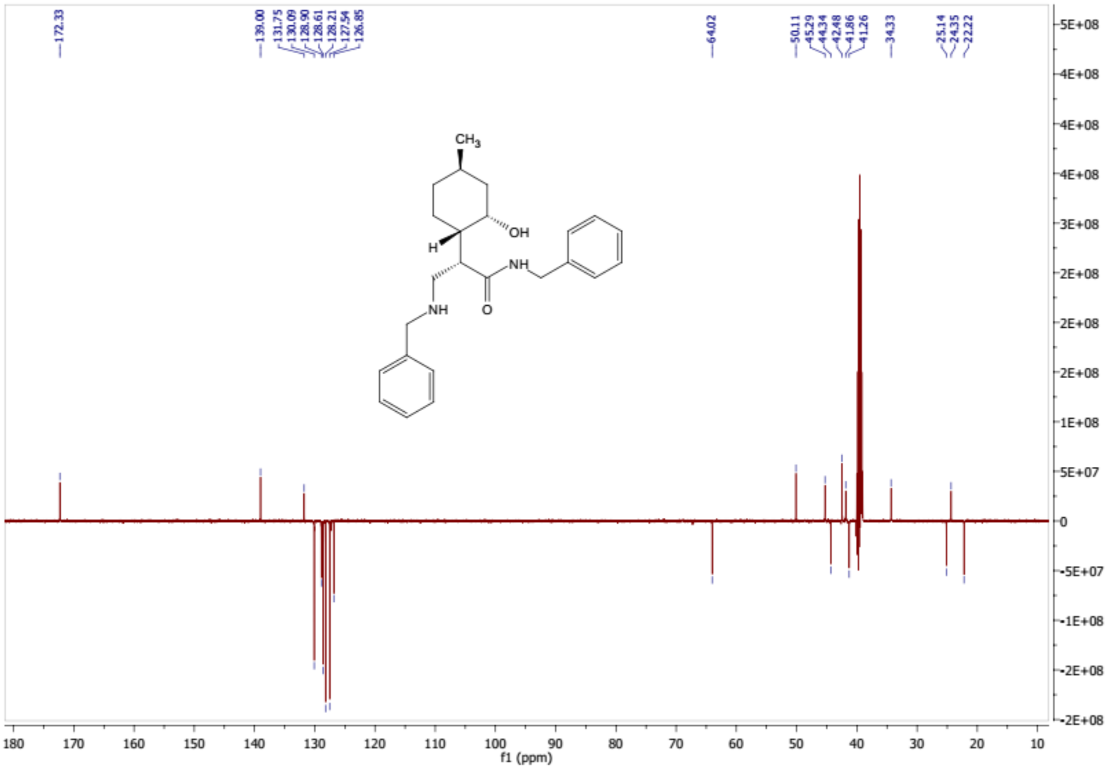

Figures S23-S24. ^{1}H (500 MHz, DMSO-d_6) and ^{13}C (125 MHz, DMSO-d_6) NMR spectra of **32.**

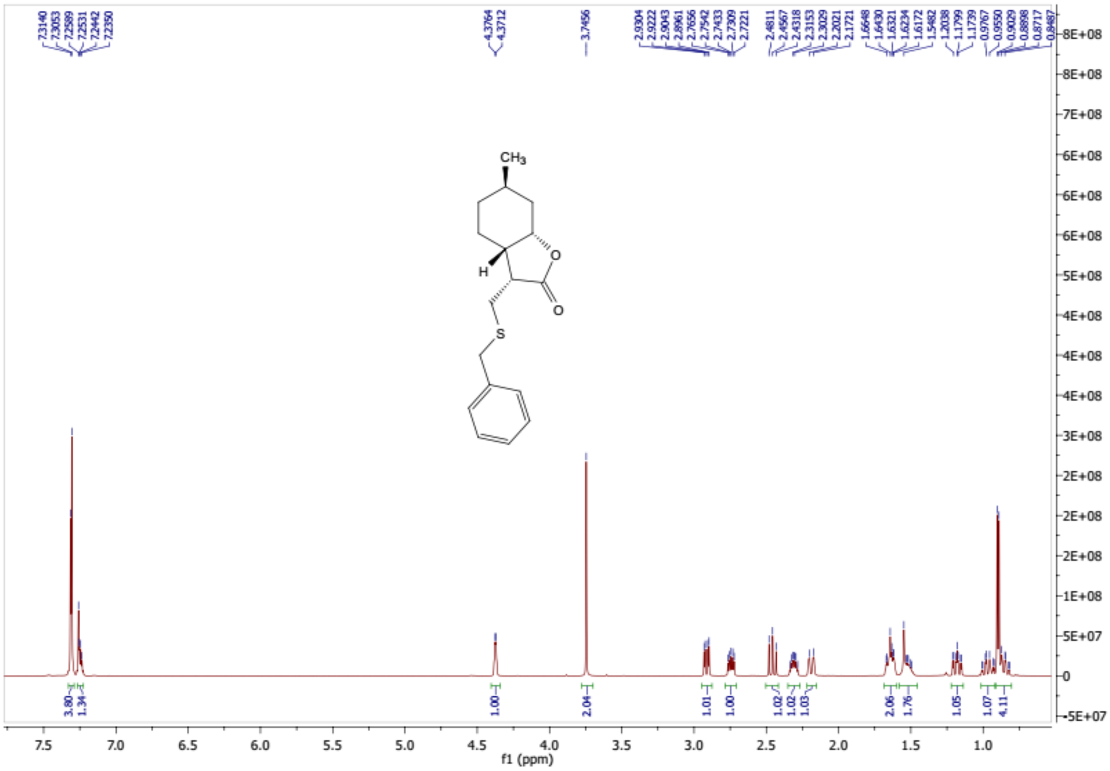

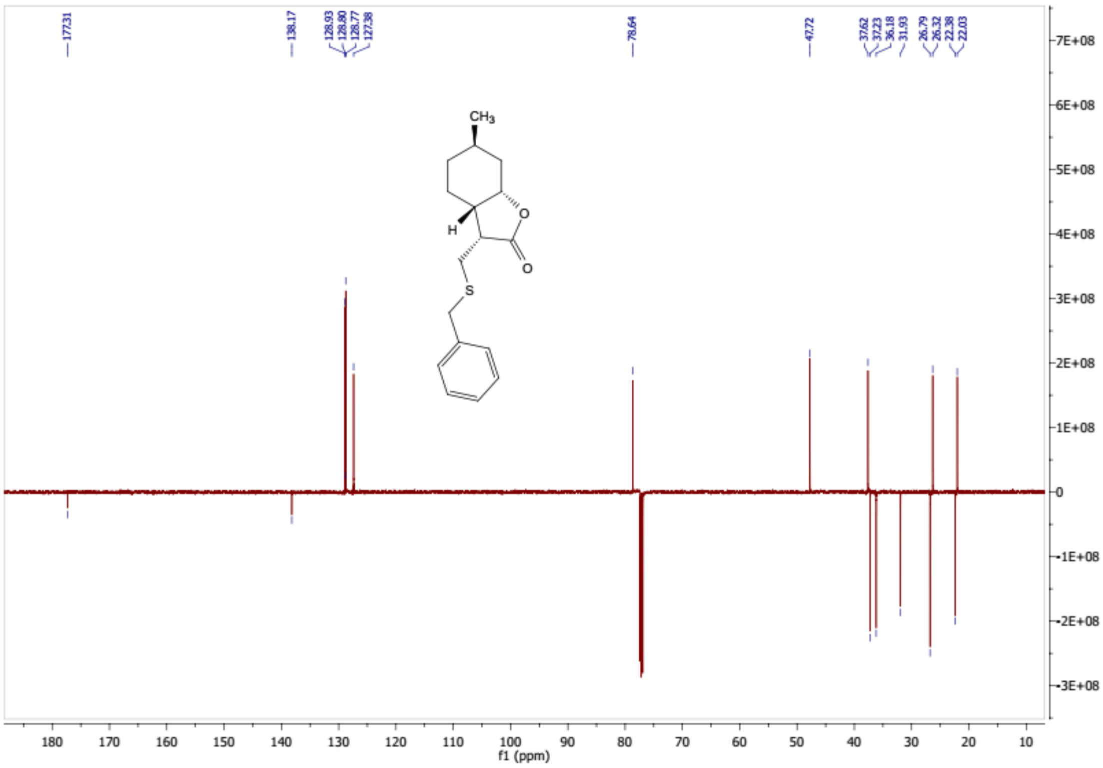

Figures S25-S26. ^{1}H (500 MHz, $CDCl_3$) and ^{13}C (125 MHz, $CDCl_3$) NMR spectra of **33.**

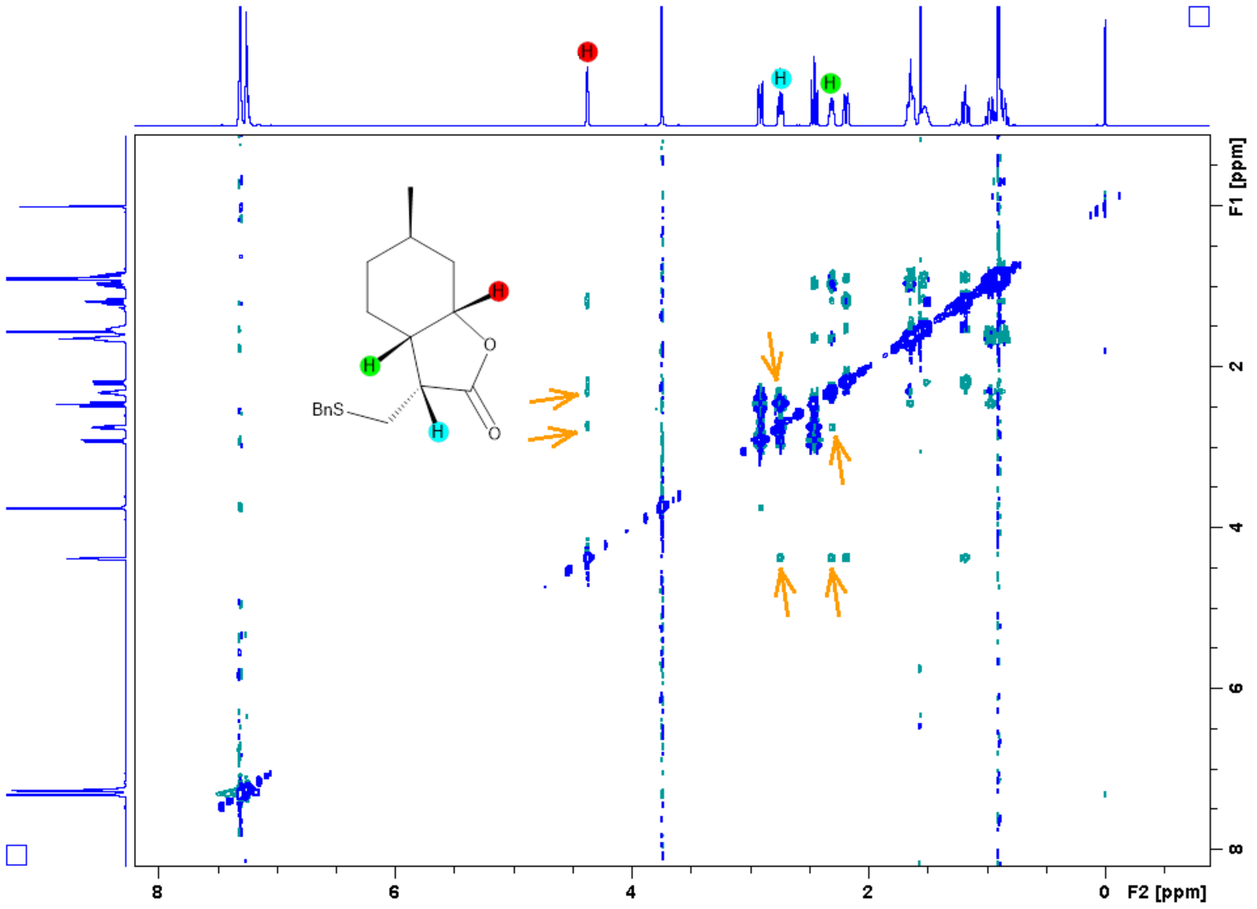

Figure S27. NOESY (500 MHz, $CDCl_3$) spectra of **33.**

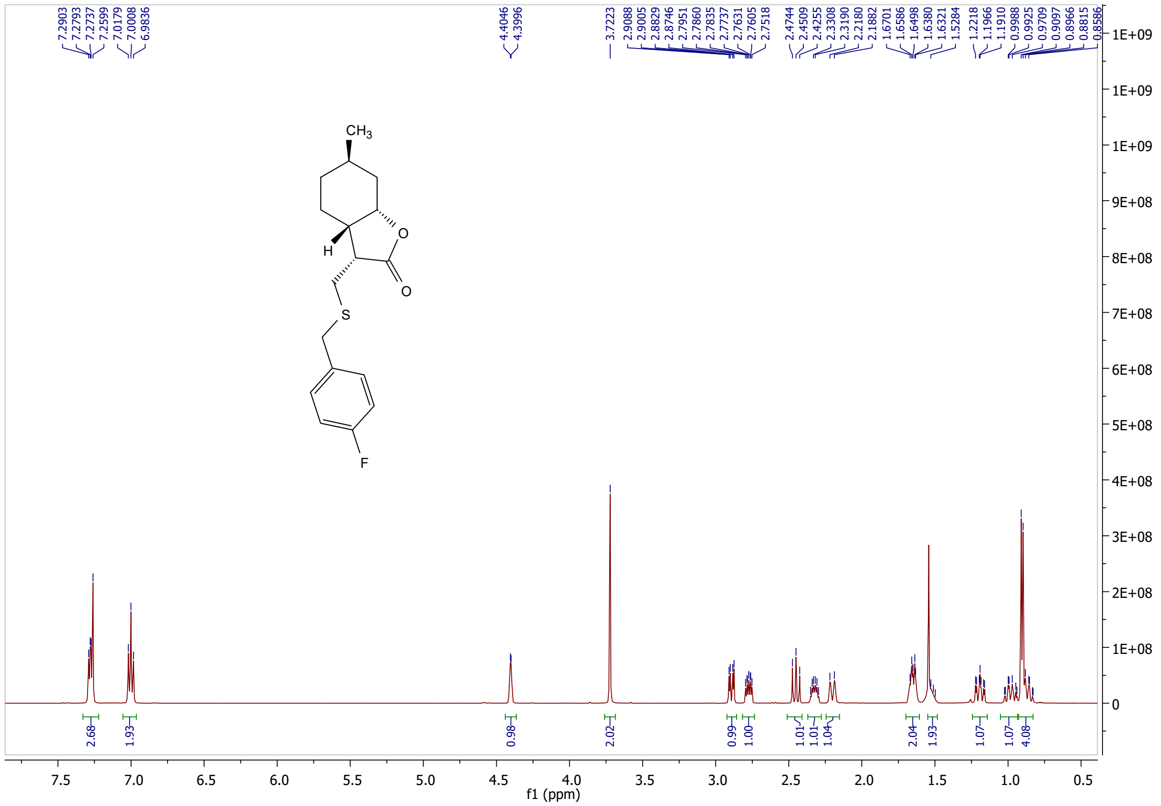

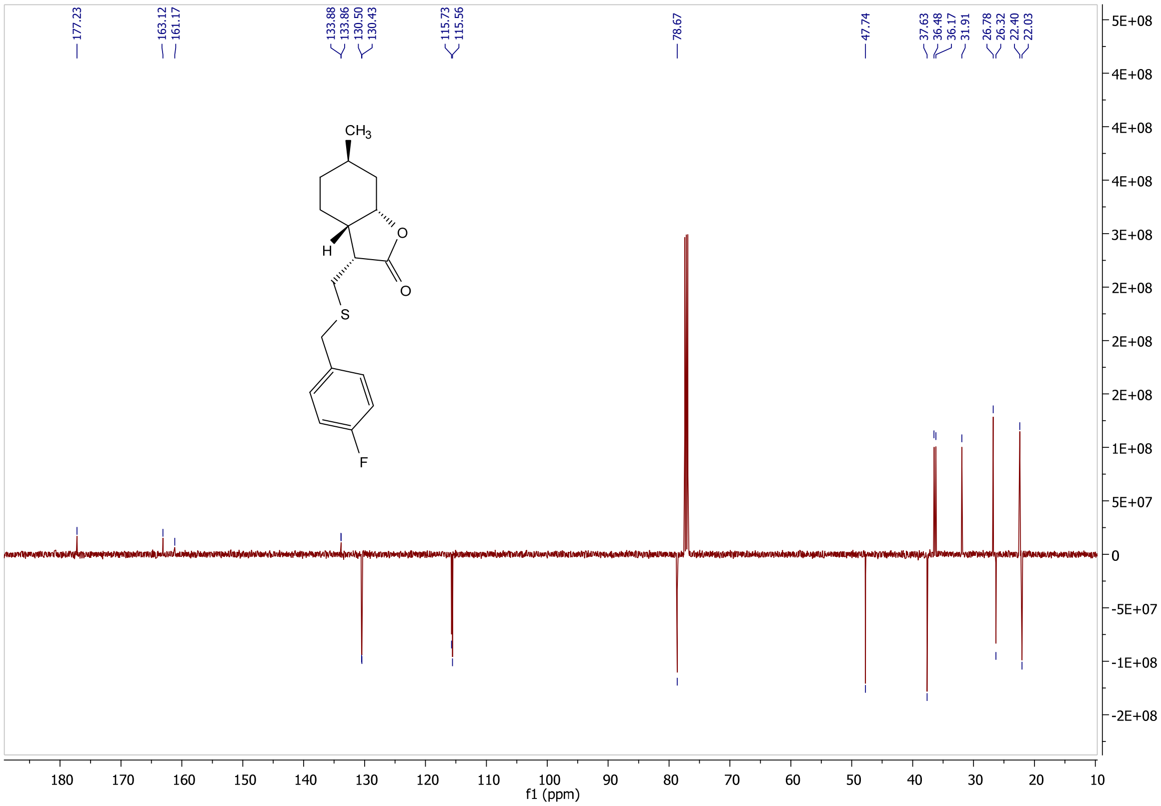

Figures S28-S29. ^{1}H (500 MHz, $CDCl_3$) and ^{13}C (125 MHz, $CDCl_3$) NMR spectra of **34.**

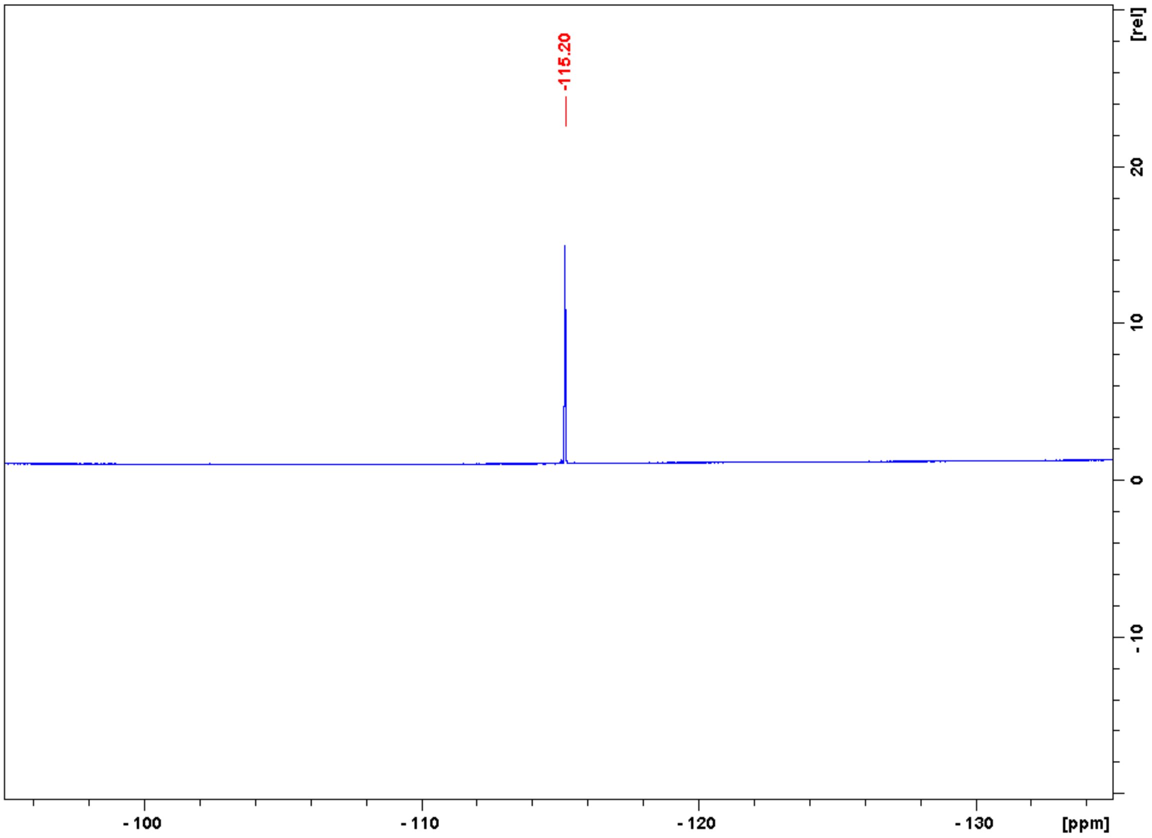

Figure S30. ^{19}F (471 MHz, $CDCl_3$) NMR spectra of **34.**

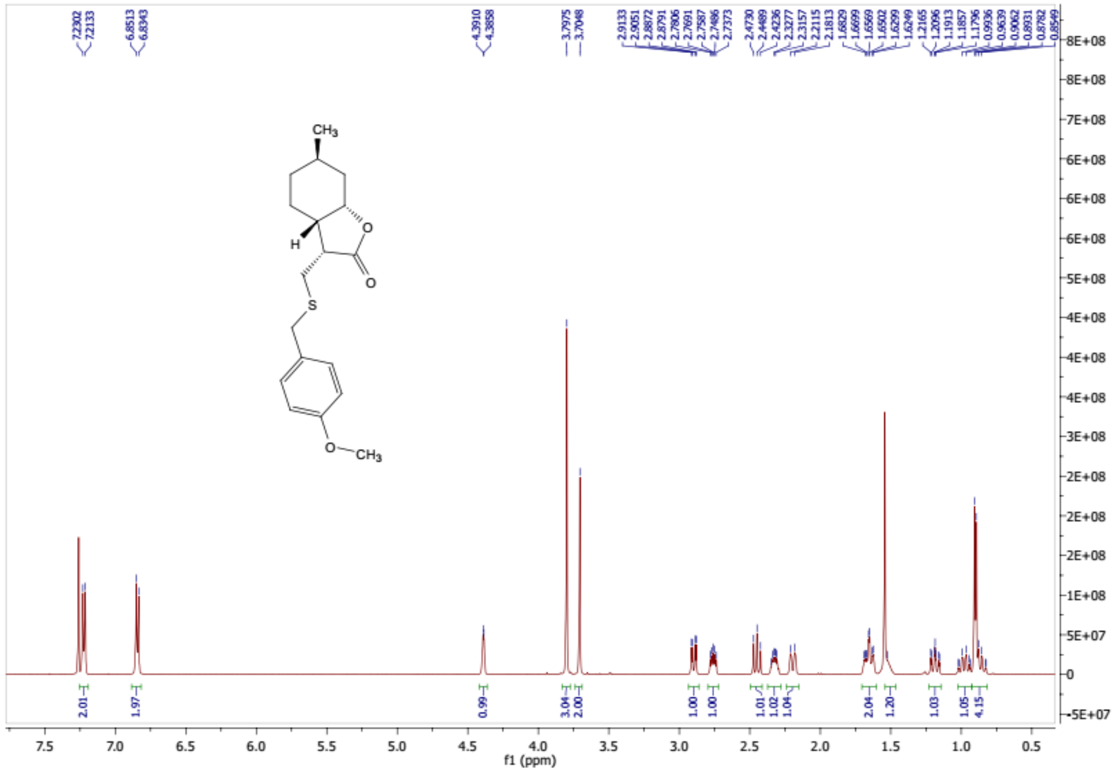

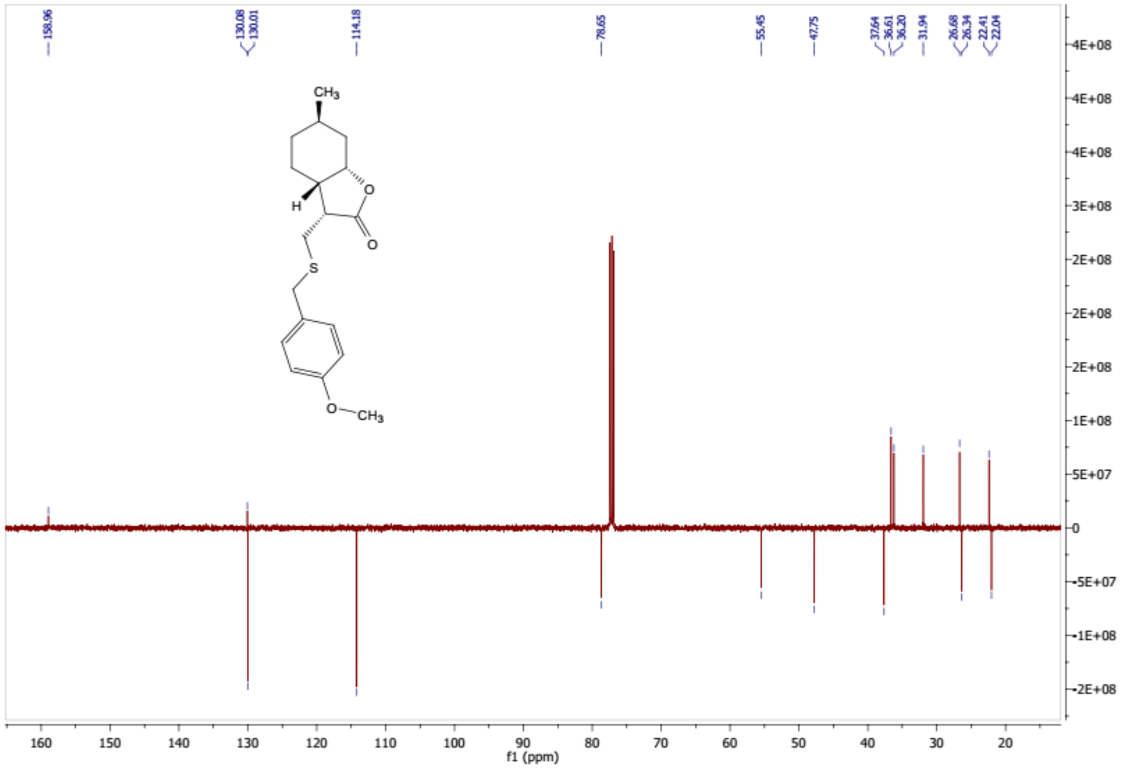

Figures S31-S32. ^{1}H (500 MHz, $CDCl_3$) and ^{13}C (125 MHz, $CDCl_3$) NMR spectra of **35.**

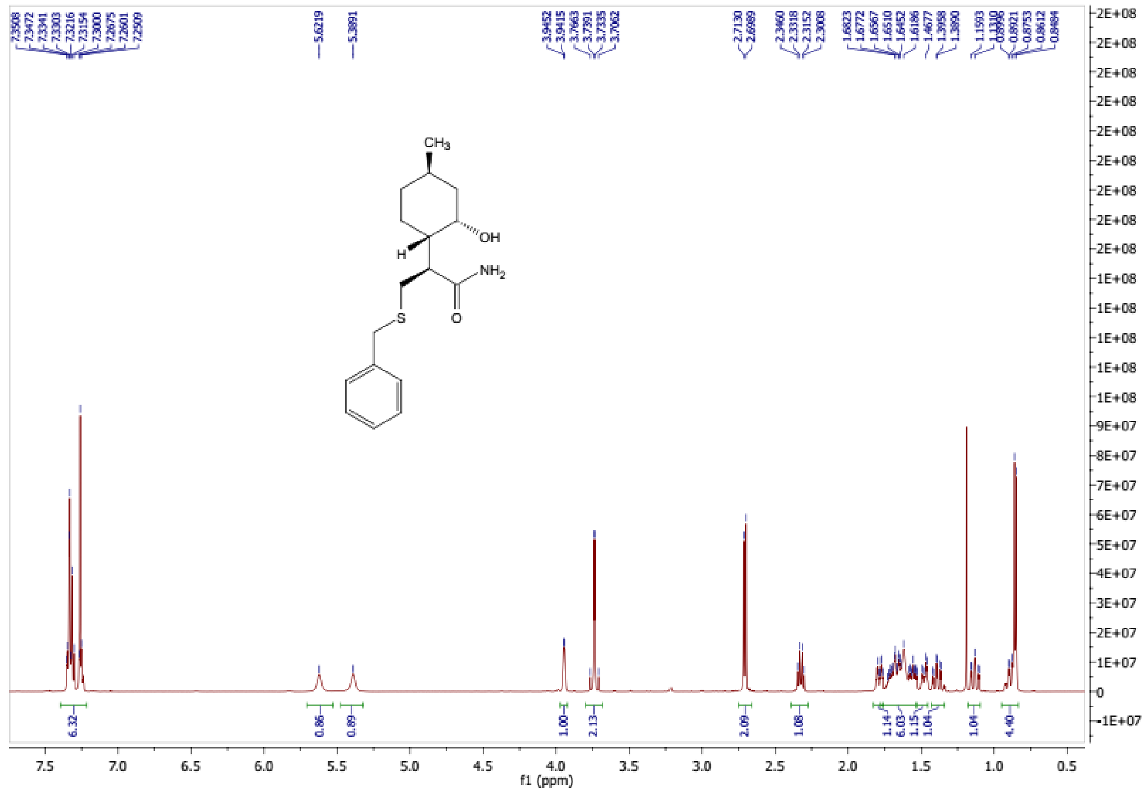

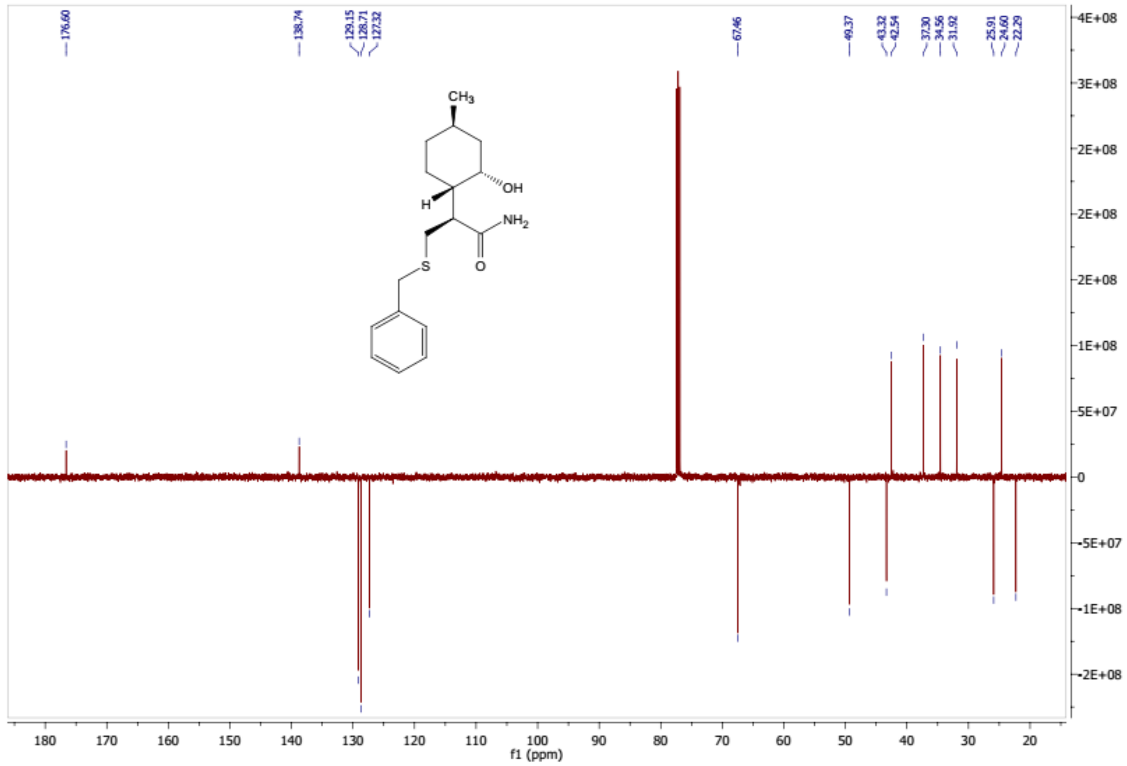

Figures S33-S34. ^{1}H (500 MHz, $CDCl_3$) and ^{13}C (125 MHz, $CDCl_3$) NMR spectra of **36.**

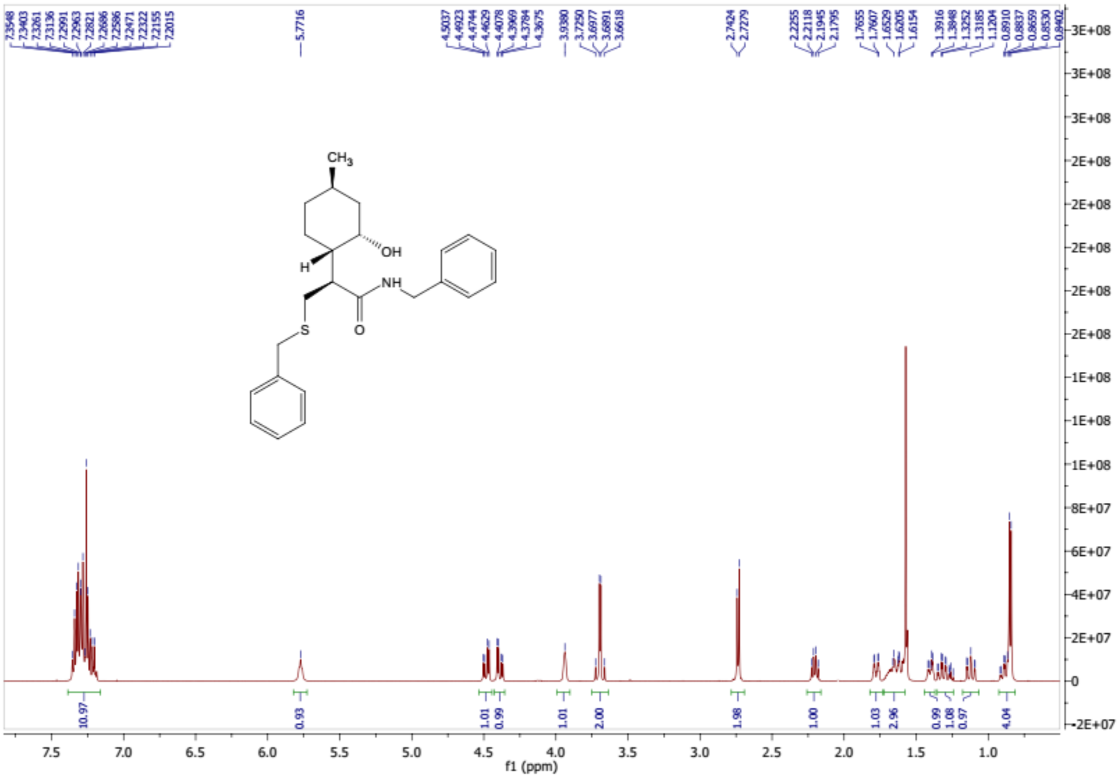

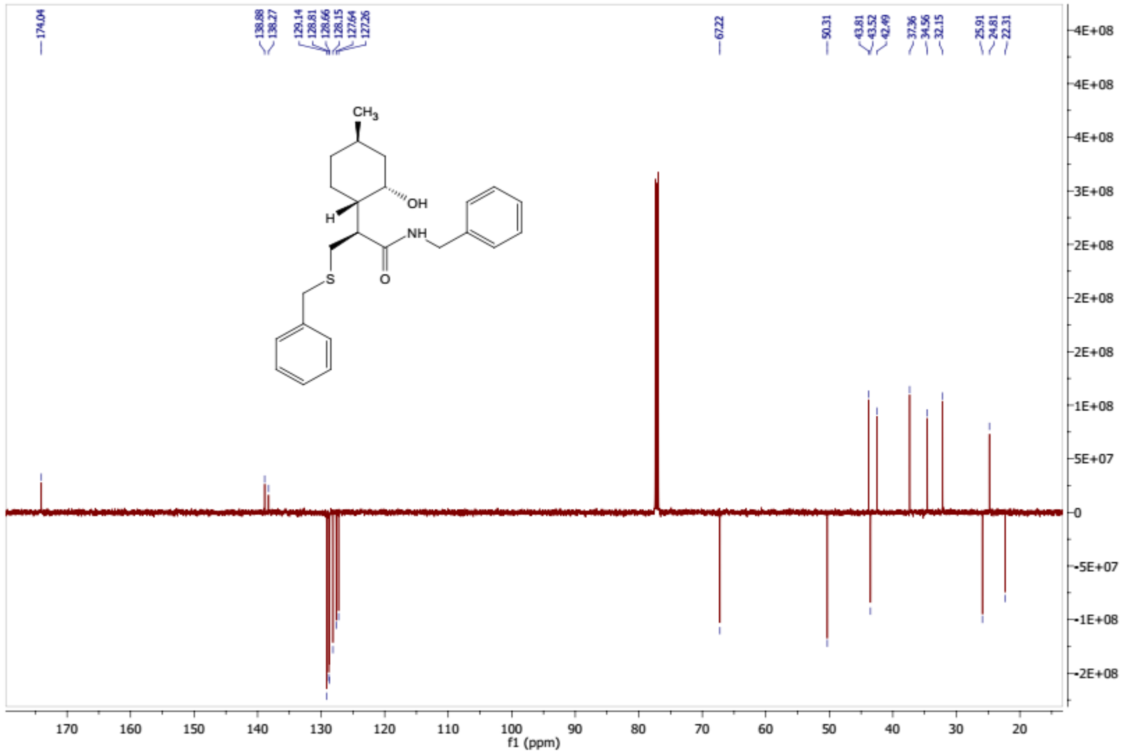

Figures S35-S36. ^{1}H (500 MHz, $CDCl_3$) and ^{13}C (125 MHz, $CDCl_3$) NMR spectra of **37.**

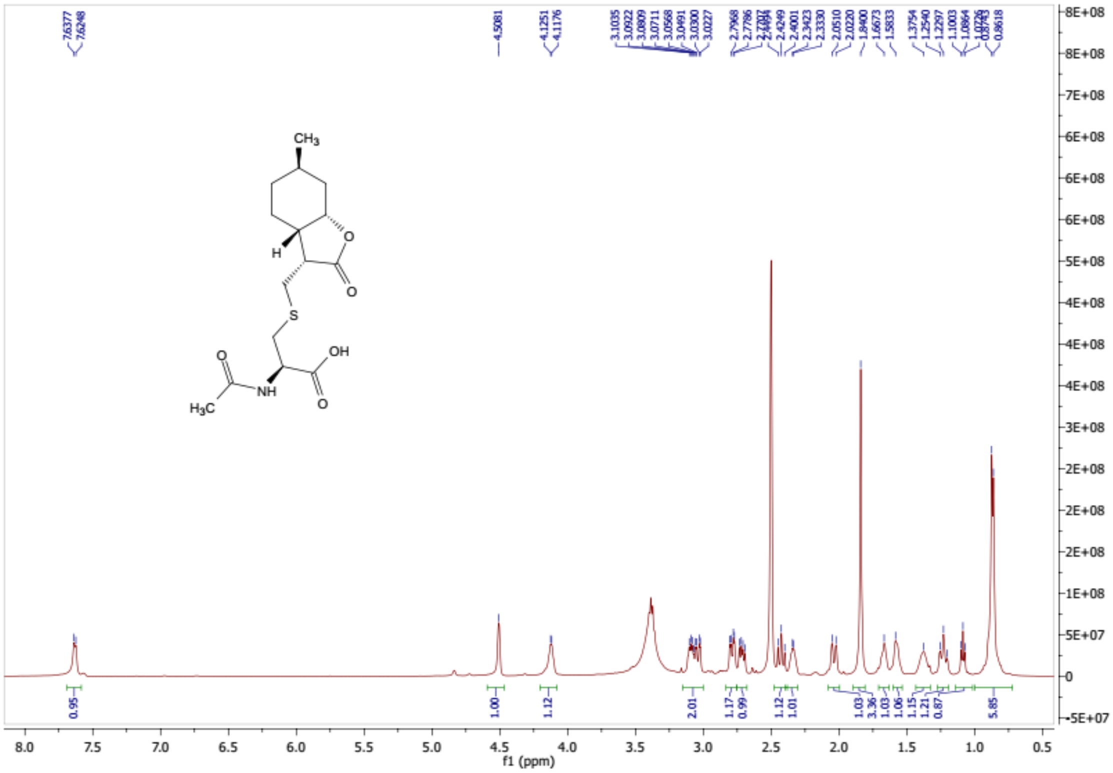

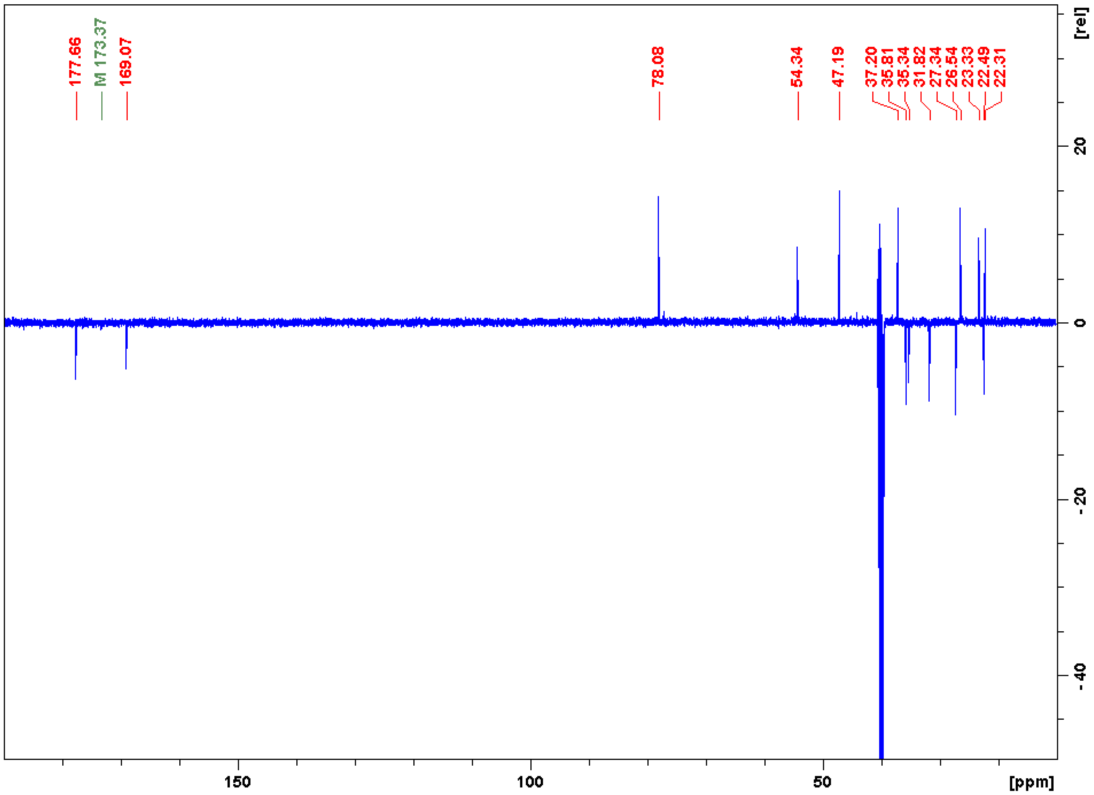

Figures S37-S38. ^{1}H (500 MHz, DMSO-*d*6) and ^{13}C (125 MHz, DMSO-*d*6) NMR spectra of **38.**

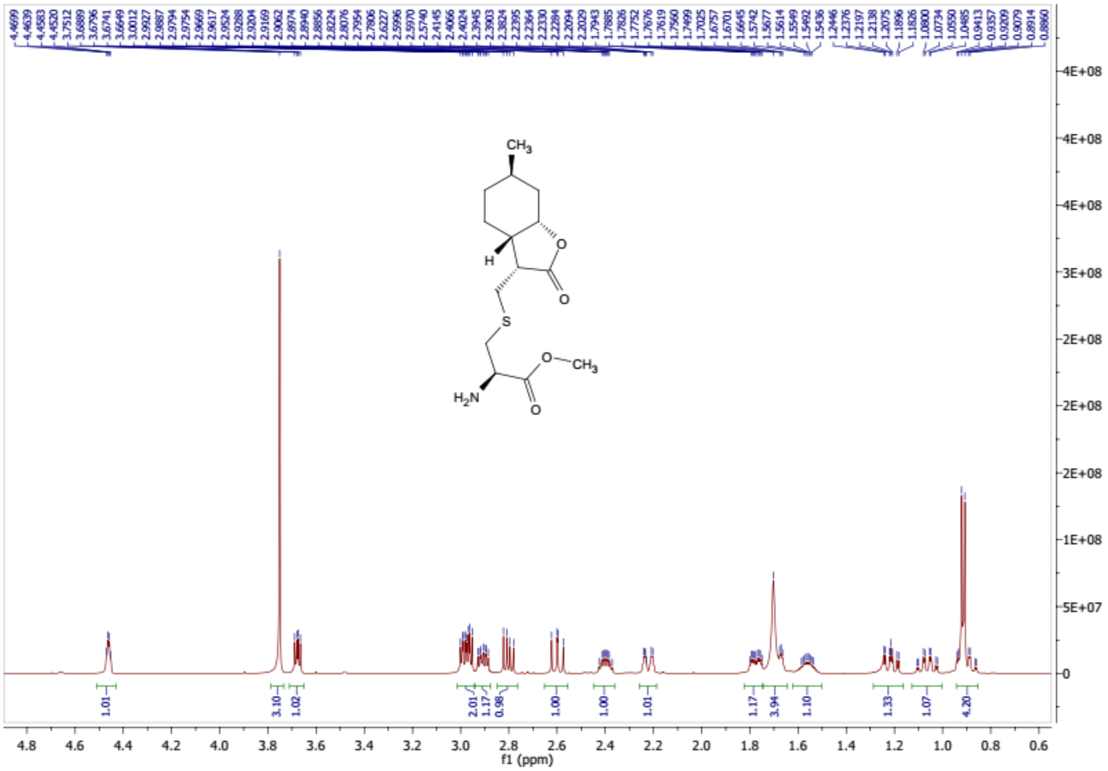

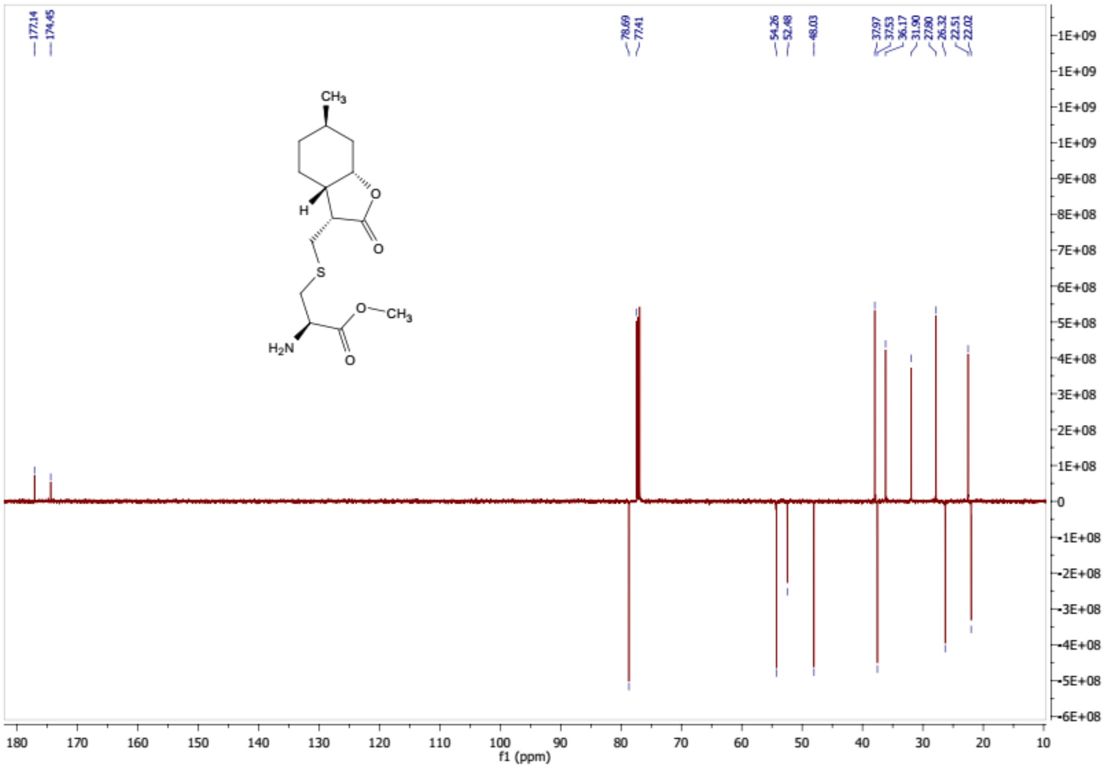

Figures S39-S40. ^{1}H (500 MHz, $CDCl_3$) and ^{13}C (125 MHz, $CDCl_3$) NMR spectra of **39.**

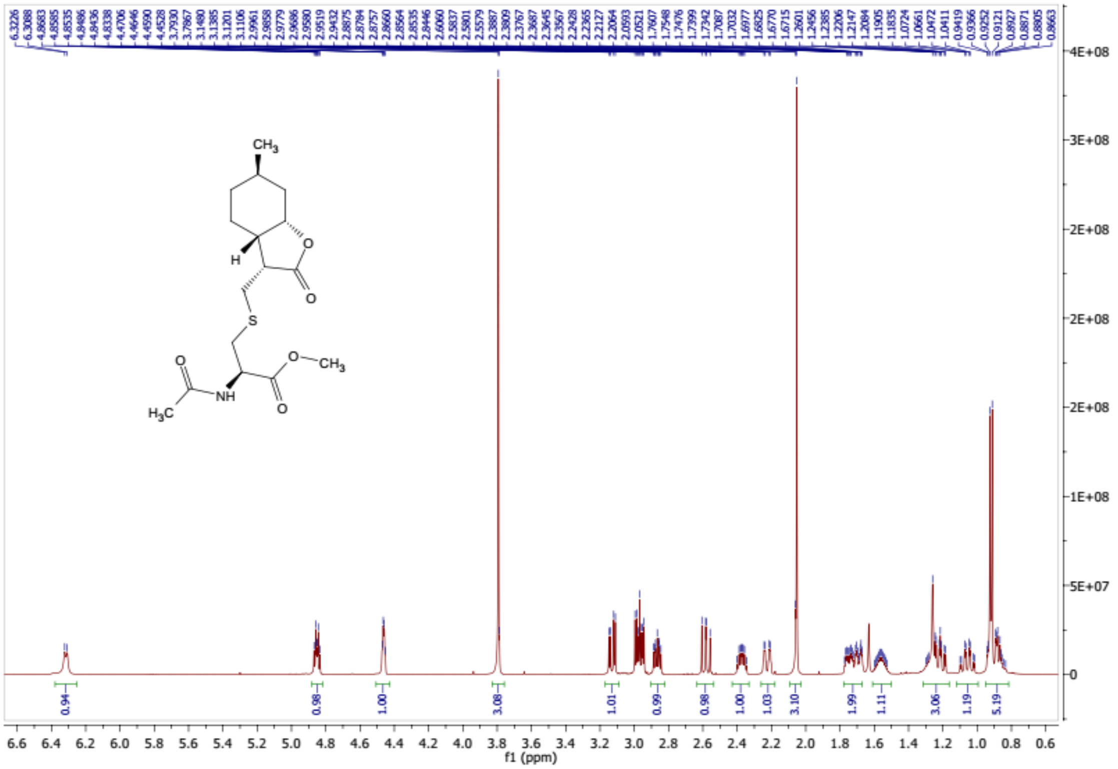

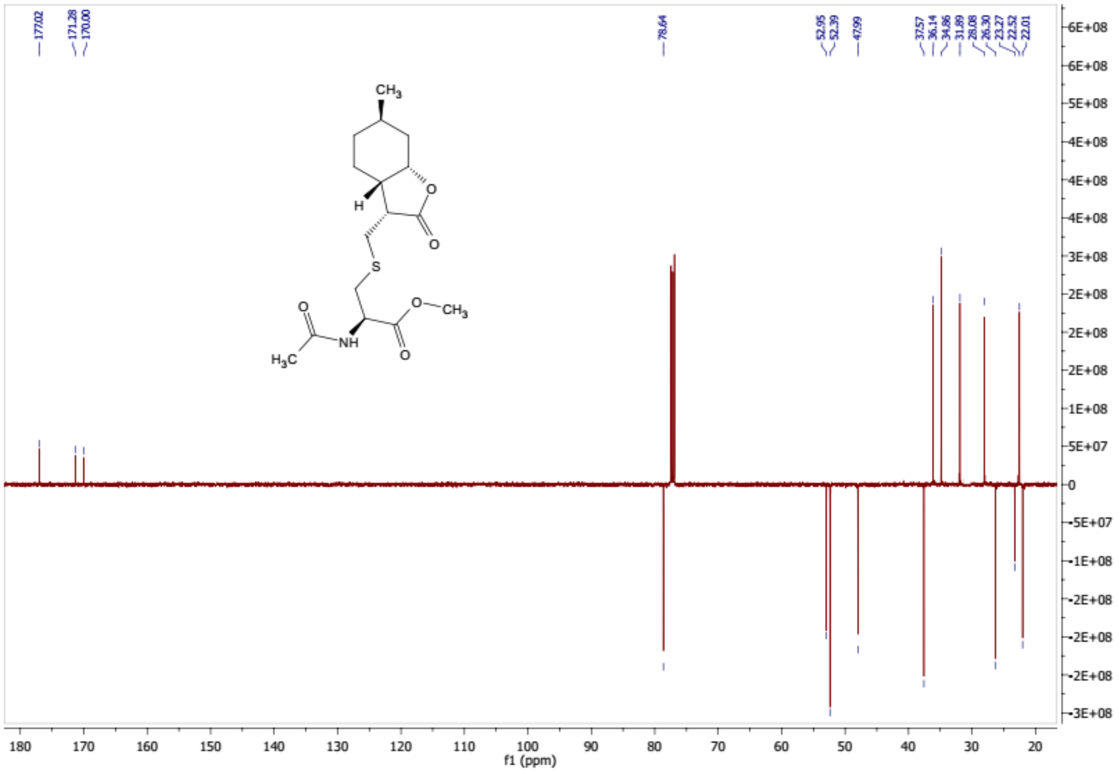

Figures S41-S42. ^{1}H (500 MHz, $CDCl_3$) and ^{13}C (125 MHz, $CDCl_3$) NMR spectra of **40.**

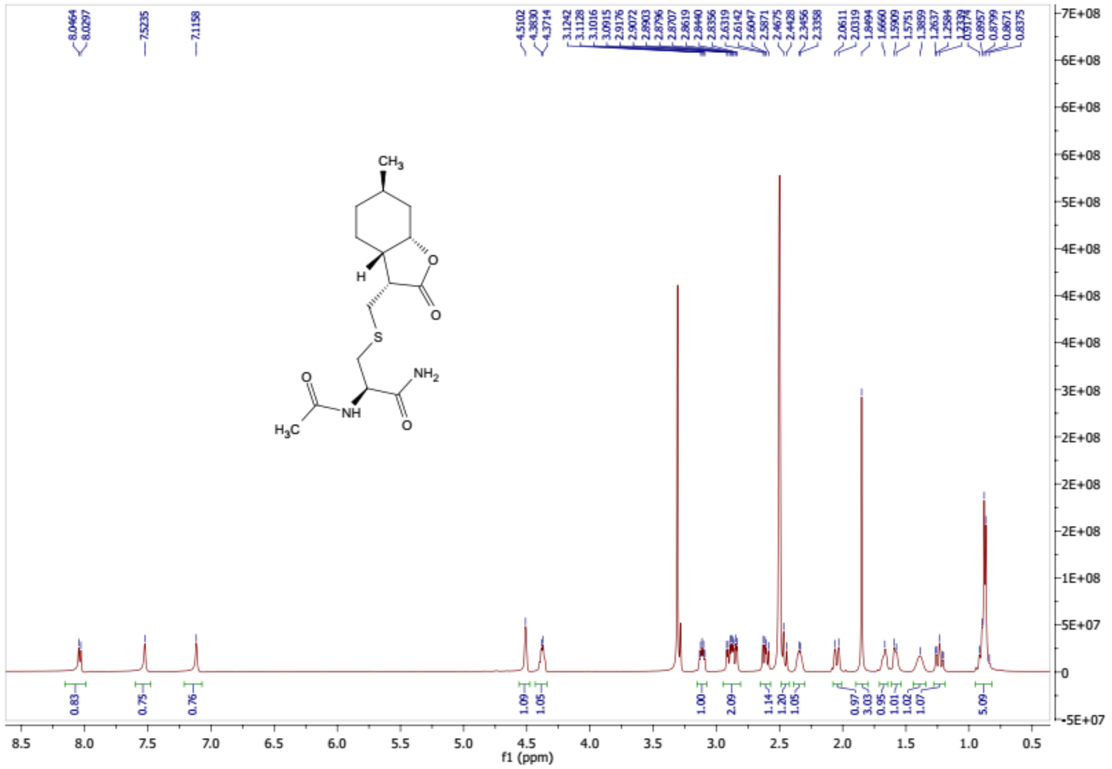

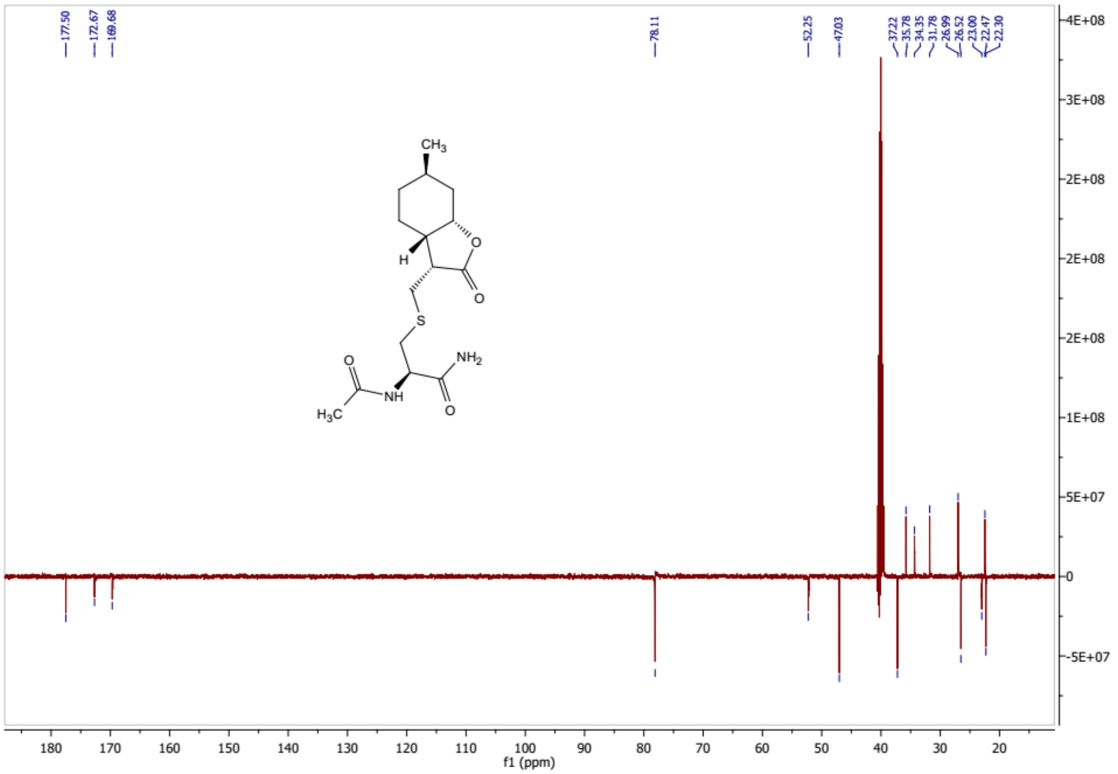

Figures S43-S44. ^{1}H (500 MHz, DMSO-d_6) and ^{13}C (125 MHz, DMSO-d_6) NMR spectra of **41.**

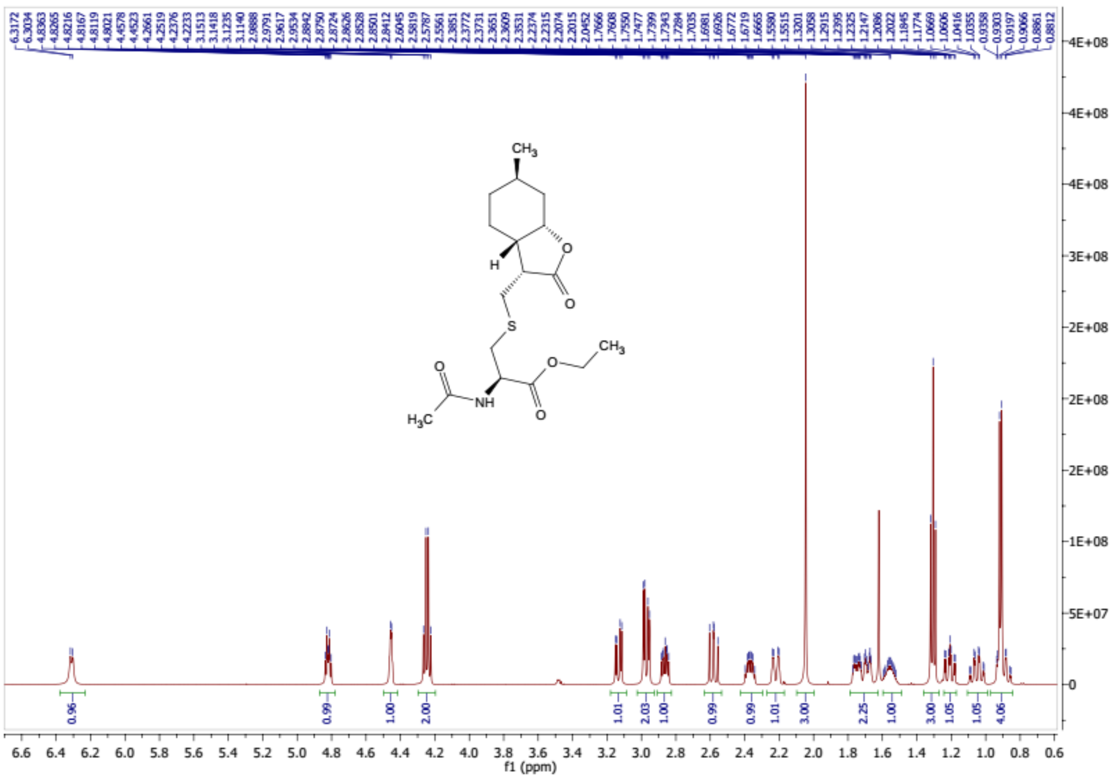

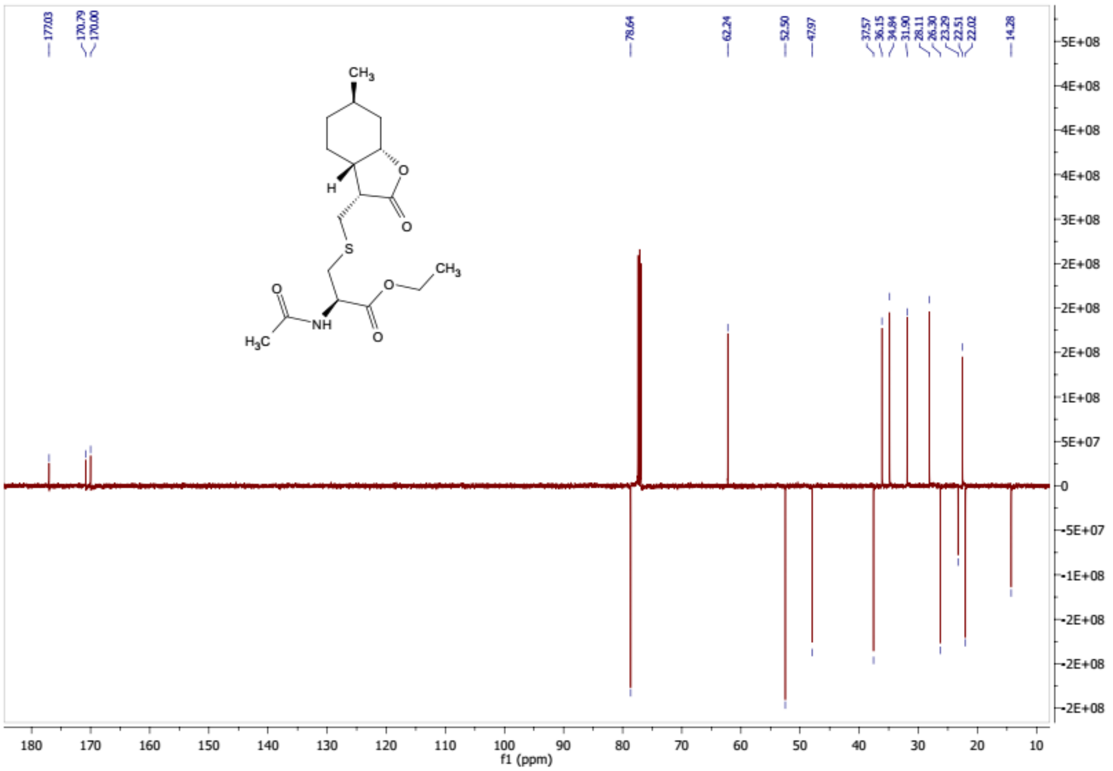

Figures S45-S46. ^{1}H (500 MHz, $CDCl_3$) and ^{13}C (125 MHz, $CDCl_3$) NMR spectra of **42.**

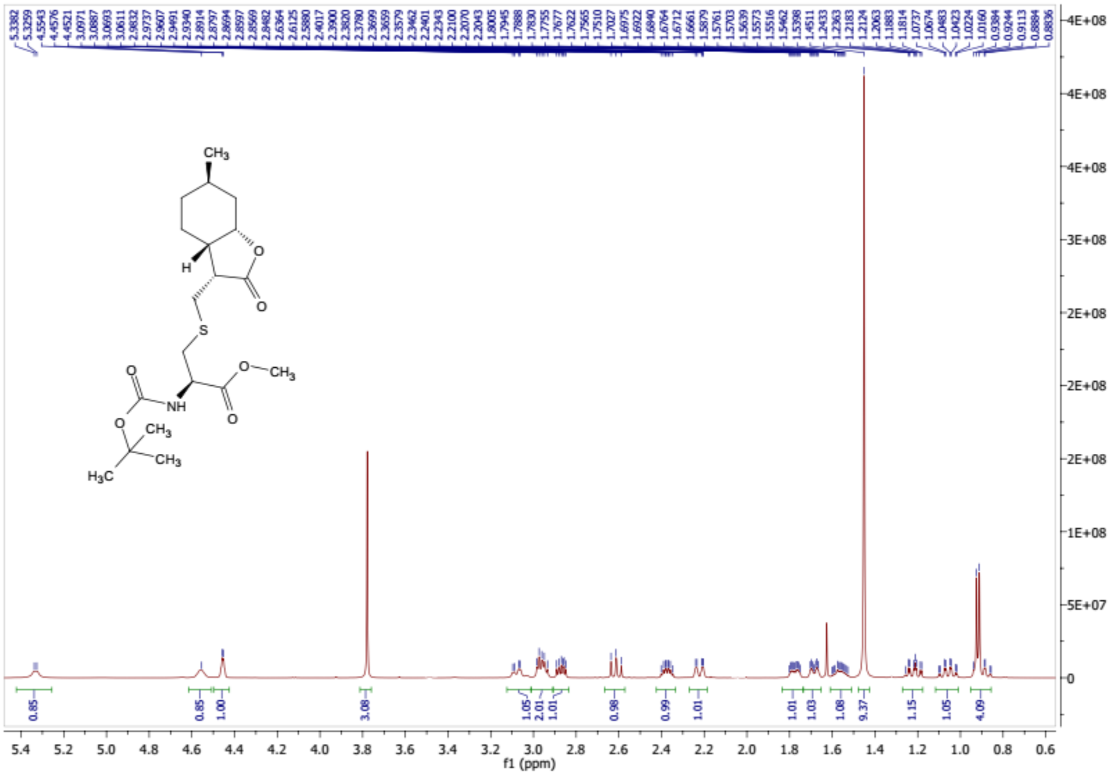

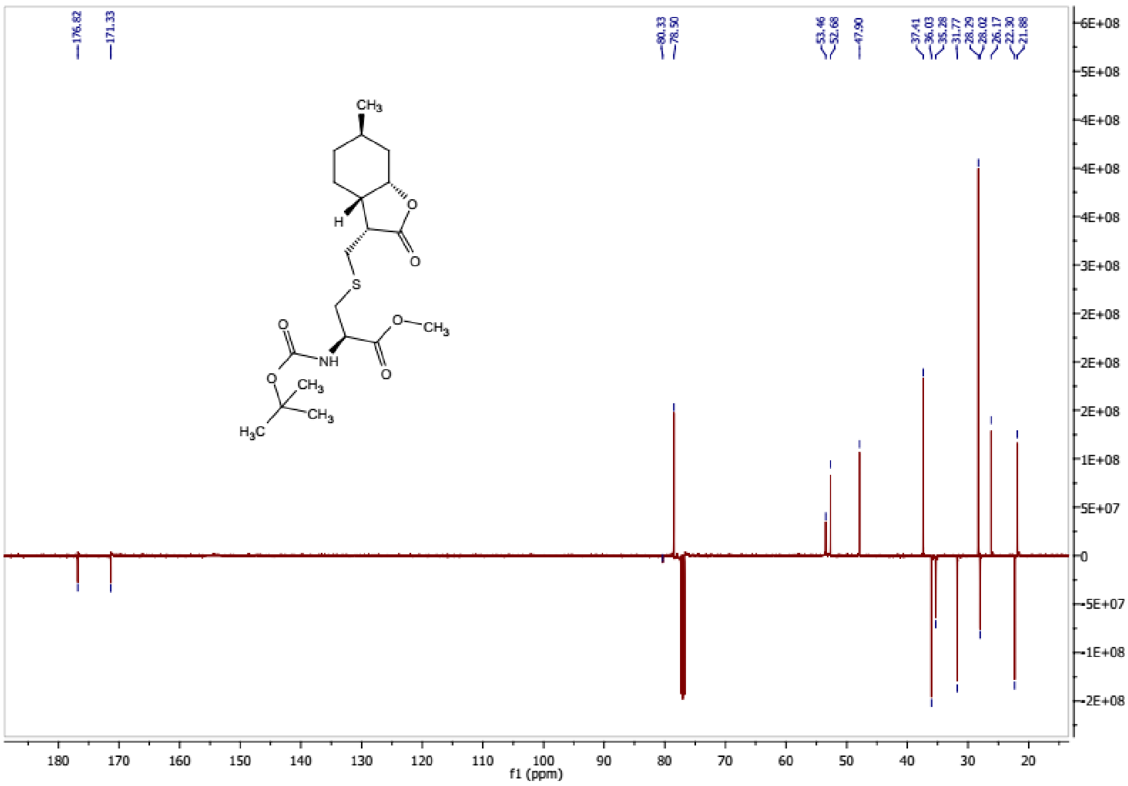

Figures S47-S48. ^{1}H (500 MHz, $CDCl_3$) and ^{13}C (125 MHz, $CDCl_3$) NMR spectra of **43.**

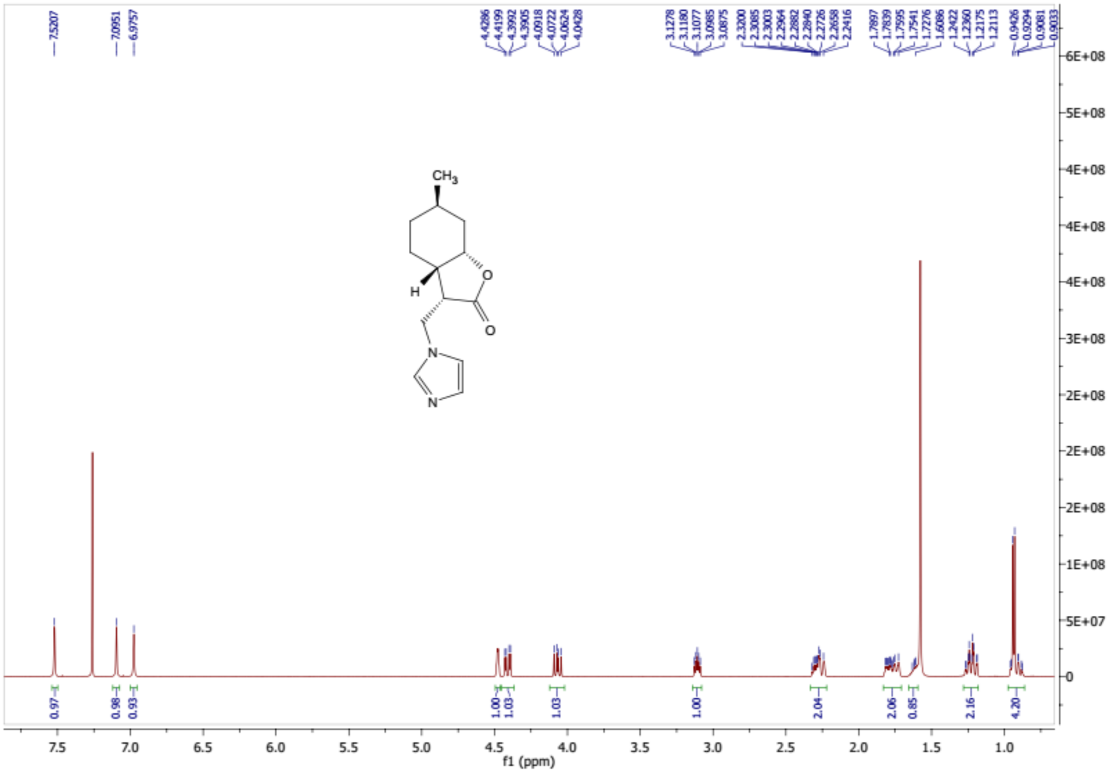

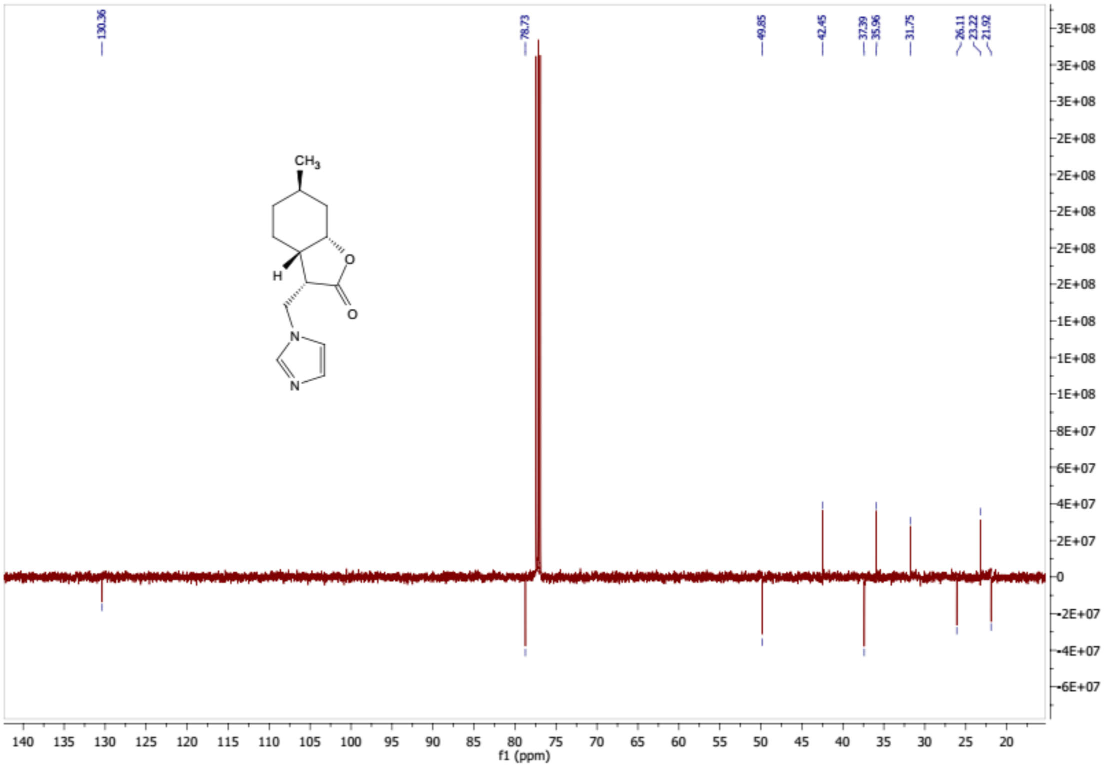

Figures S49-S50. ^{1}H (500 MHz, $CDCl_3$) and ^{13}C (125 MHz, $CDCl_3$) NMR spectra of **44a.**

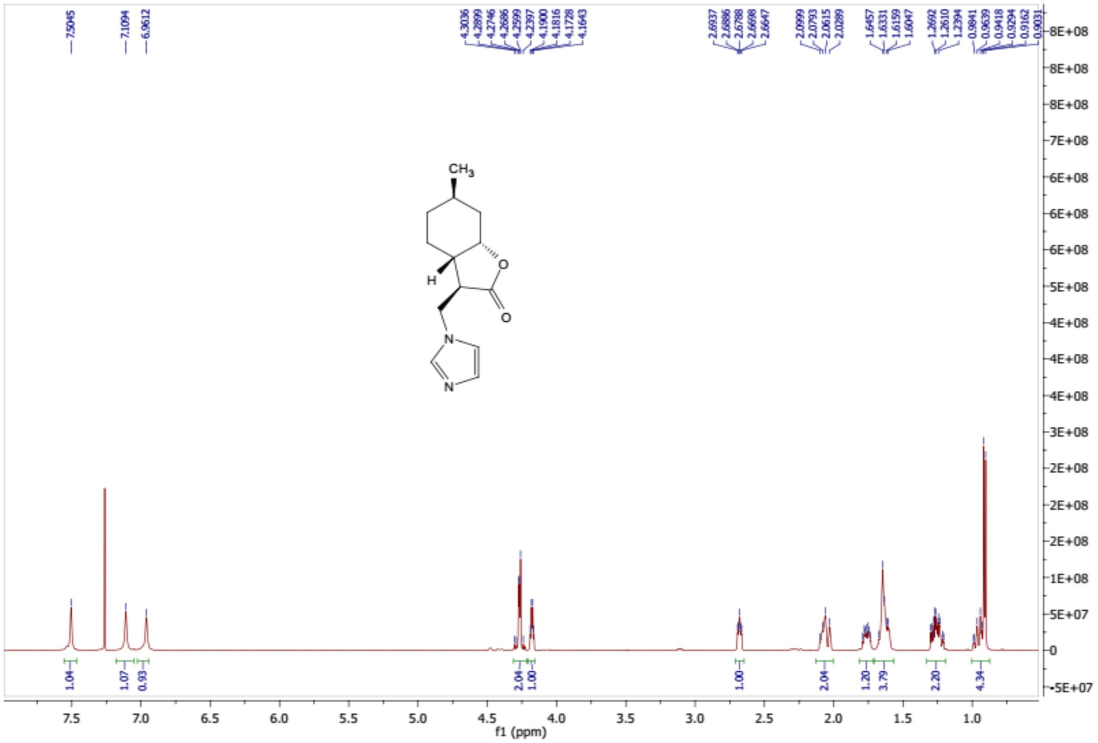

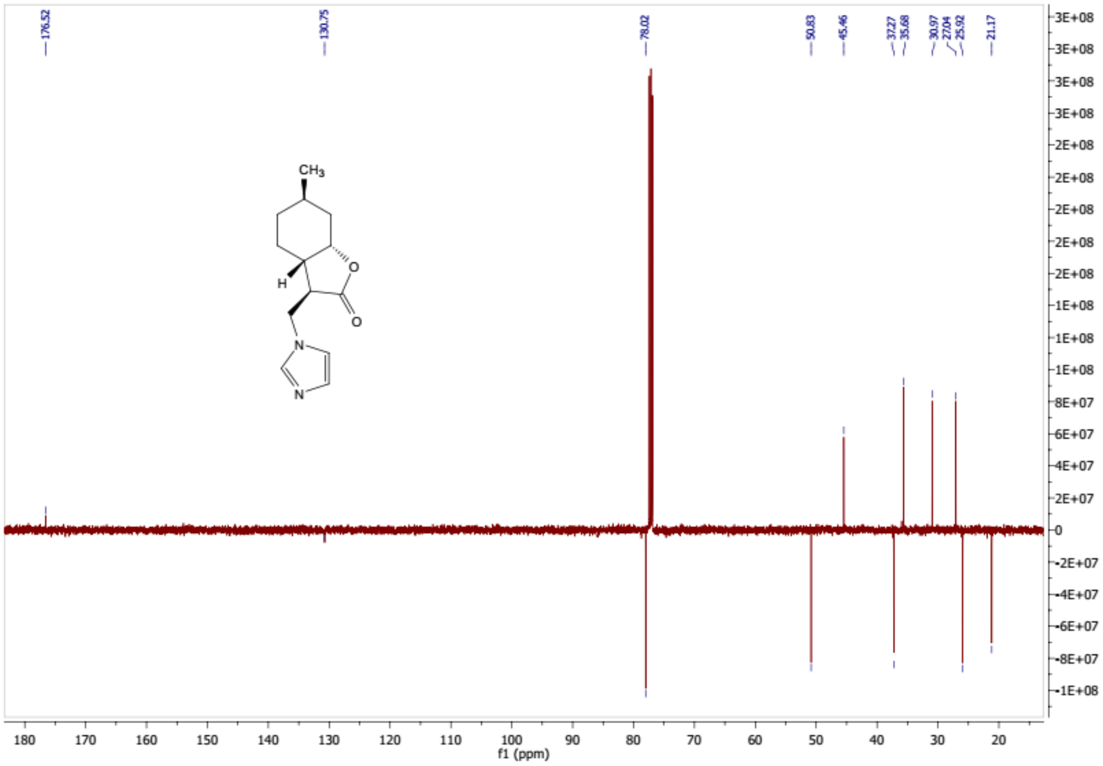

Figures S51-S52. ^{1}H (500 MHz, $CDCl_3$) and ^{13}C (125 MHz, $CDCl_3$) NMR spectra of **44b.**

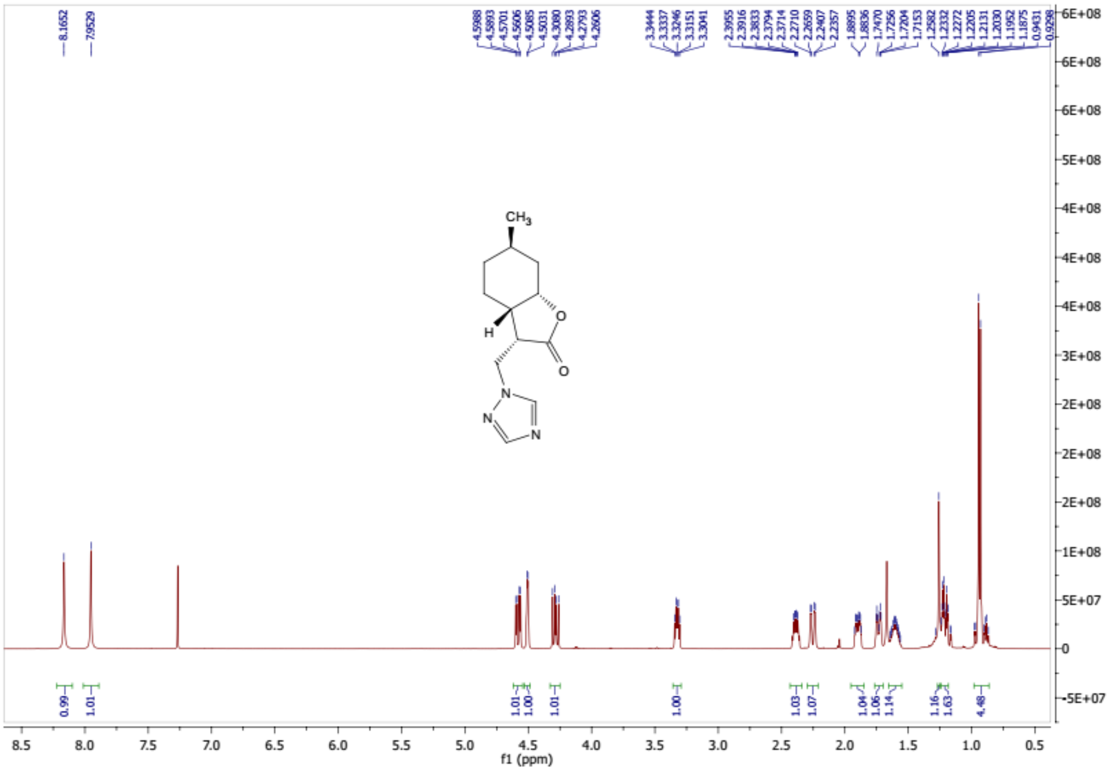

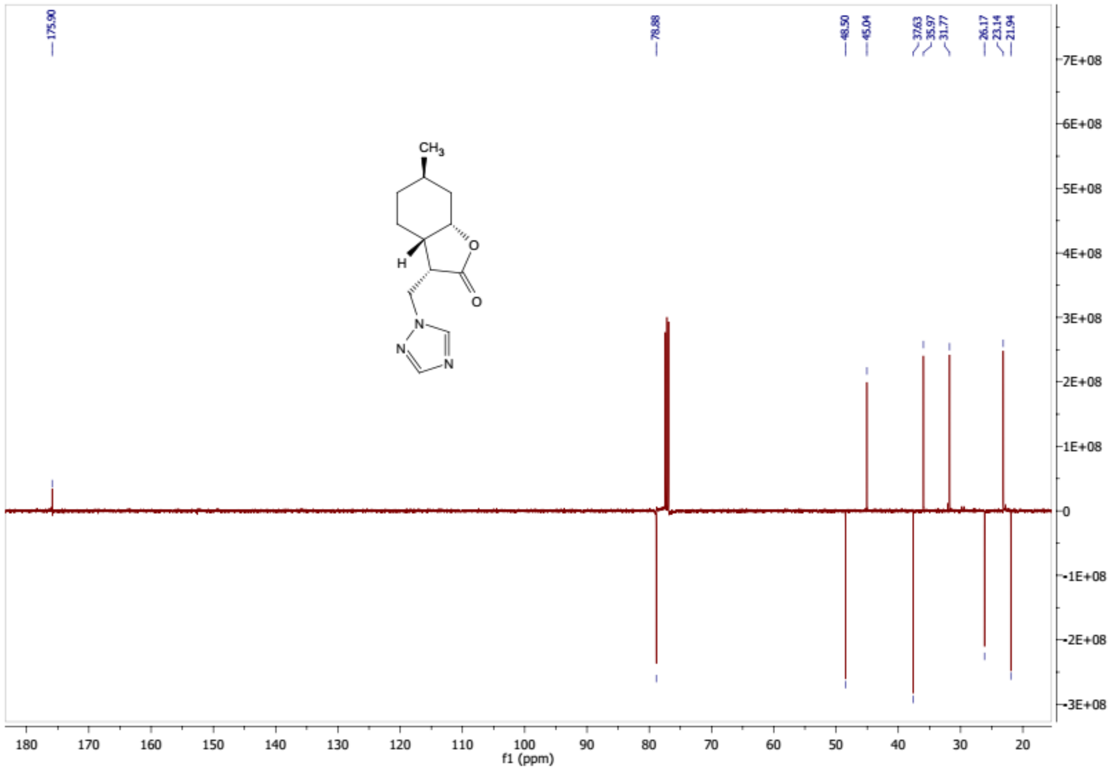

Figures S53-S54. ^{1}H (500 MHz, $CDCl_3$) and ^{13}C (125 MHz, $CDCl_3$) NMR spectra of **45a.**

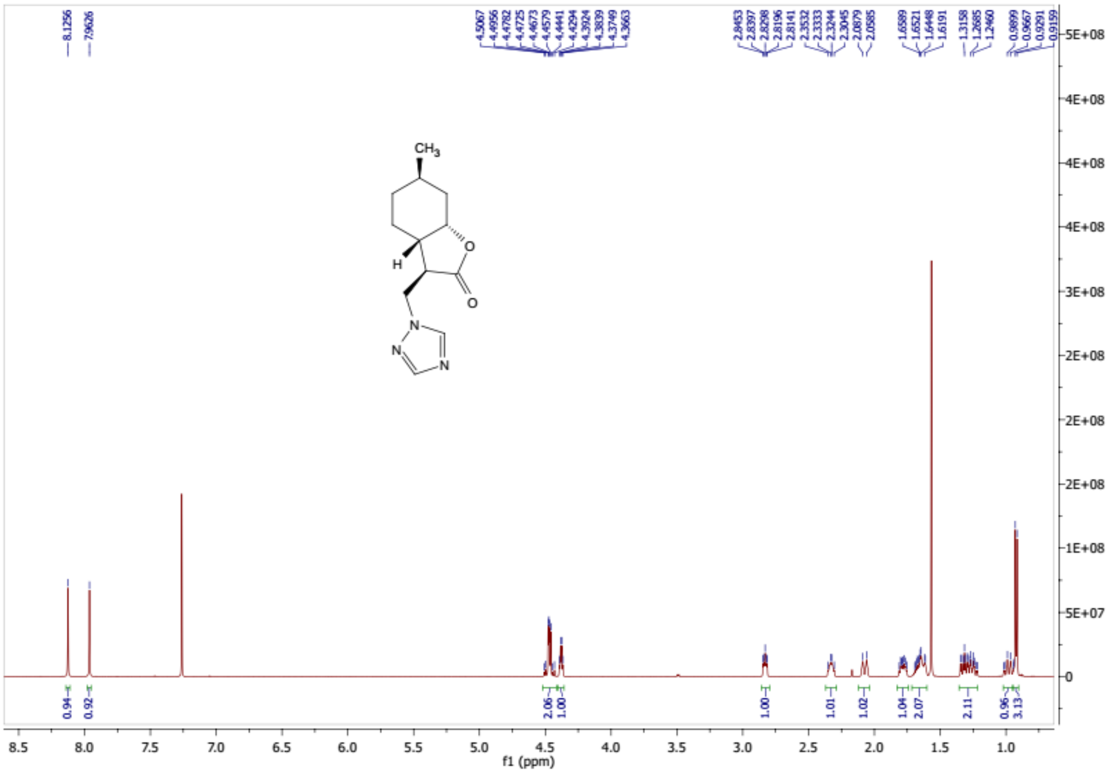

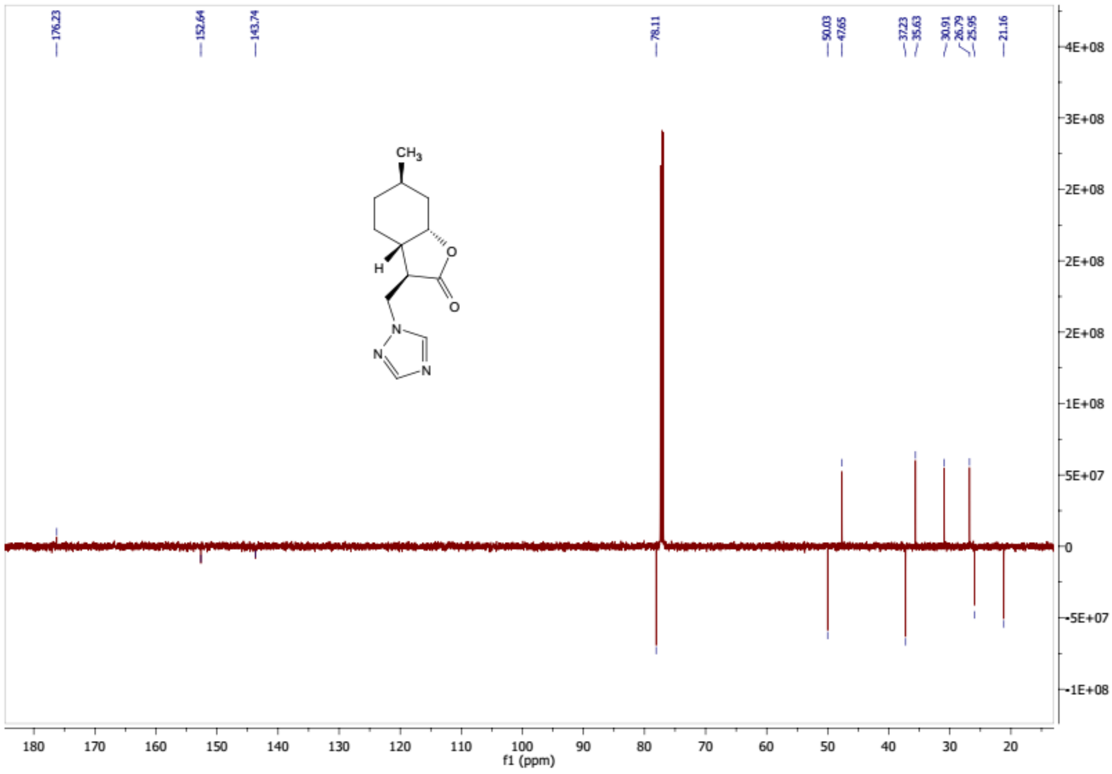

Figures S55-S56. ^{1}H (500 MHz, $CDCl_3$) and ^{13}C (125 MHz, $CDCl_3$) NMR spectra of **45b.**

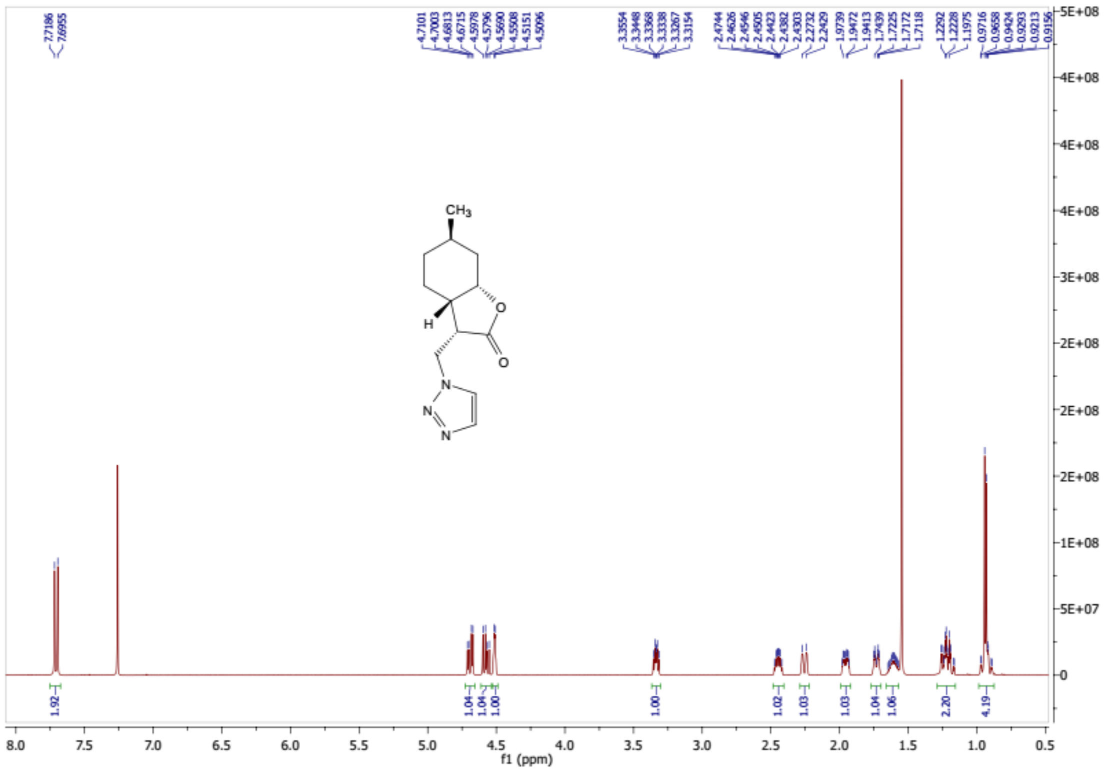

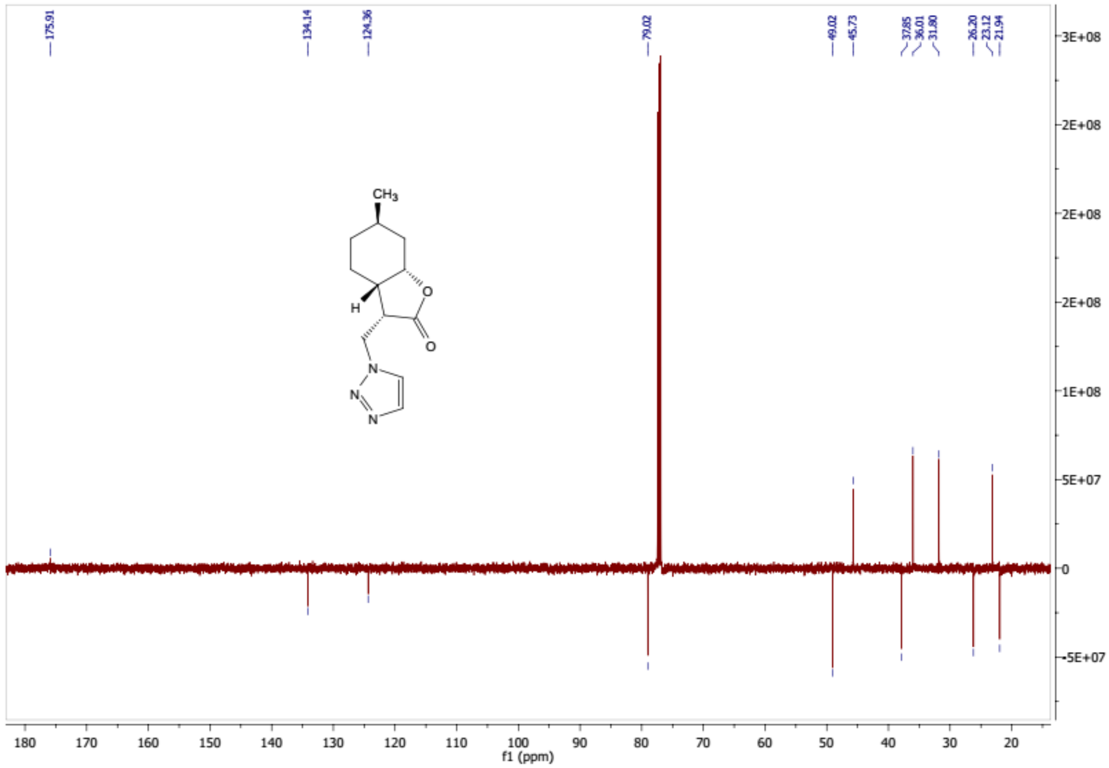

Figures S57-S58. ^{1}H (500 MHz, $CDCl_3$) and ^{13}C (125 MHz, $CDCl_3$) NMR spectra of **46a.**

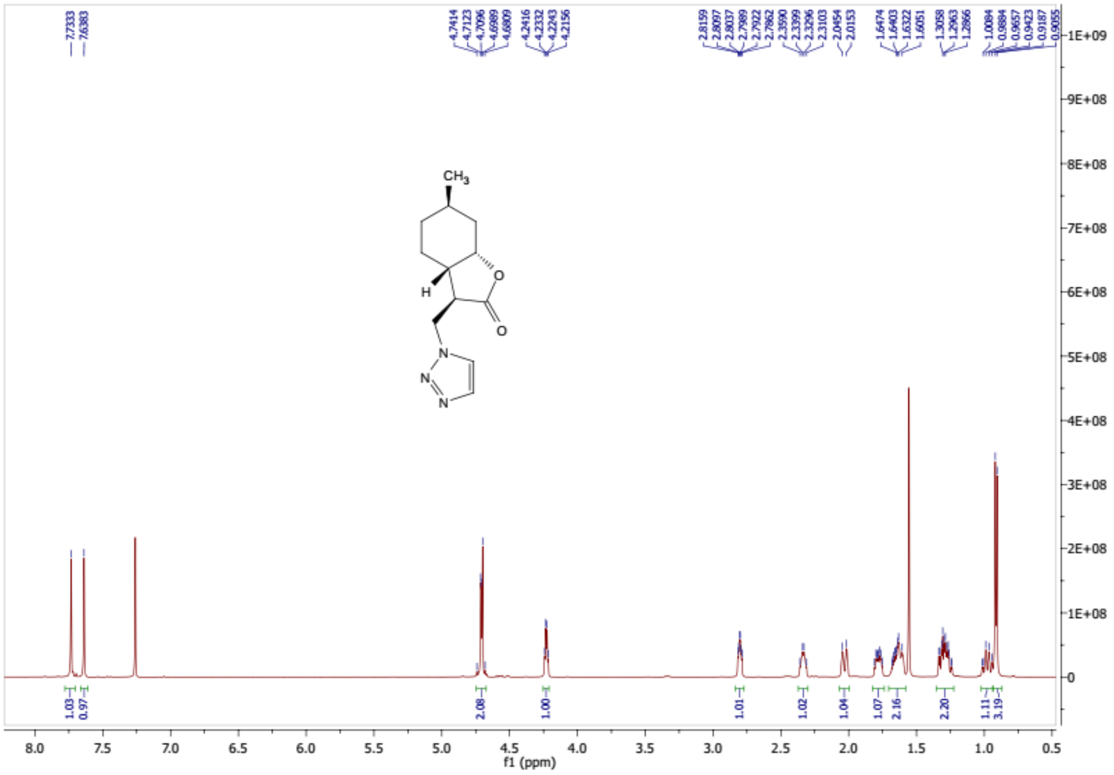

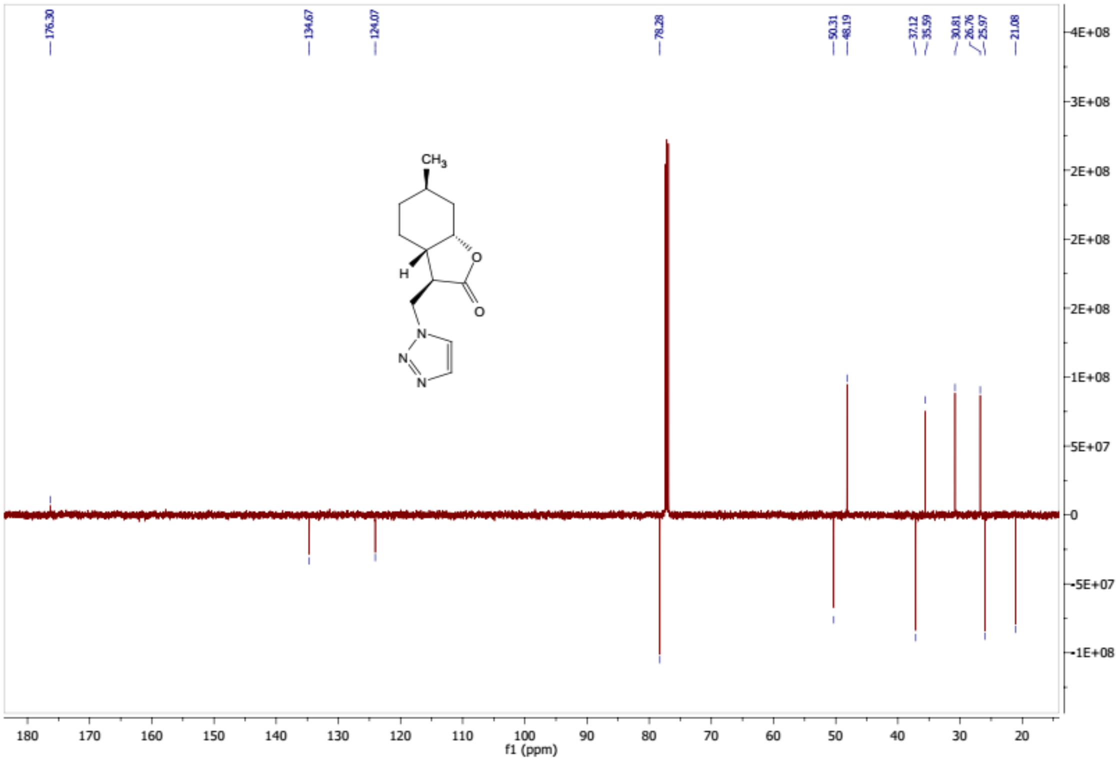

Figures S59-S60. ^{1}H (500 MHz, $CDCl_3$) and ^{13}C (125 MHz, $CDCl_3$) NMR spectra of **46b.**

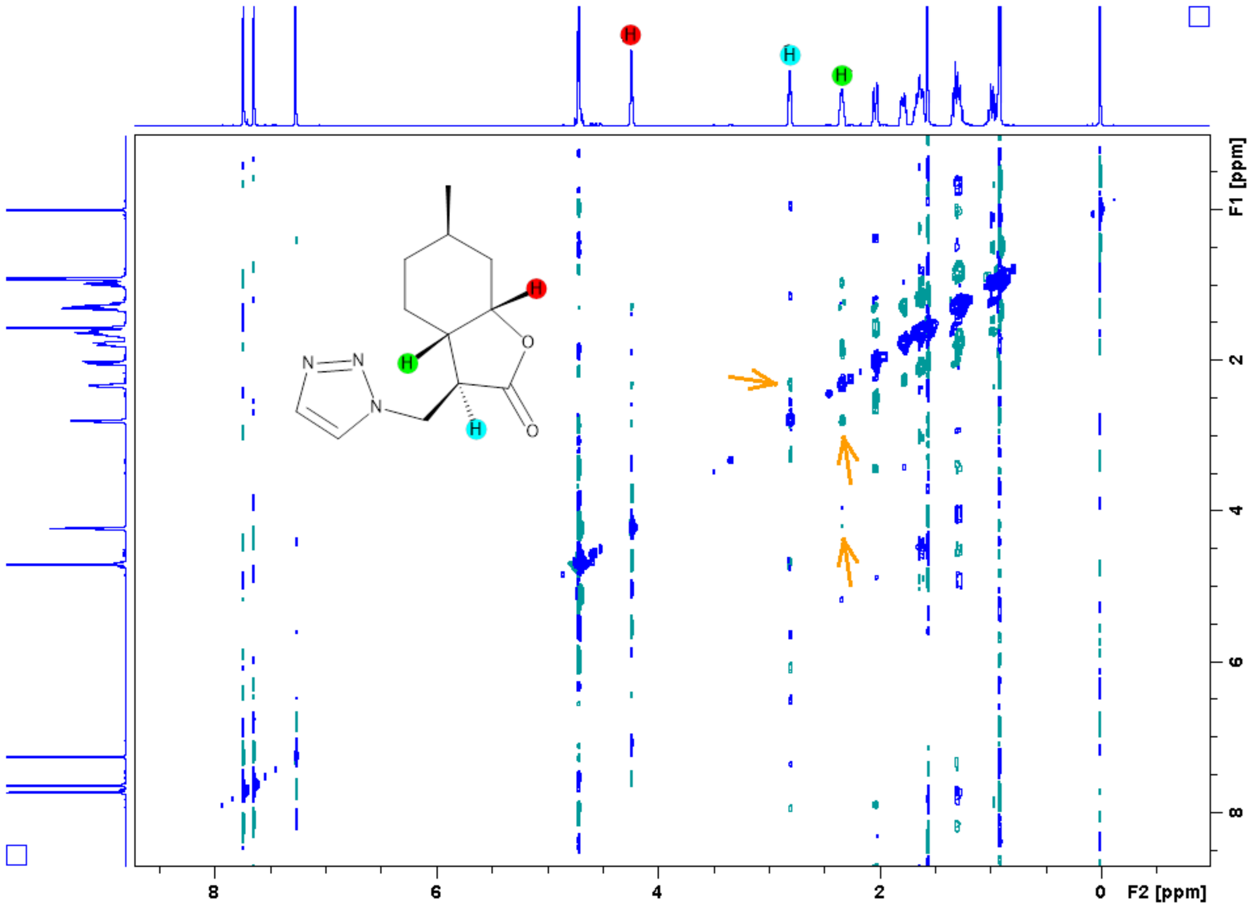

Figure S61. NOESY (500 MHz, $CDCl_3$) spectra of **46b.**

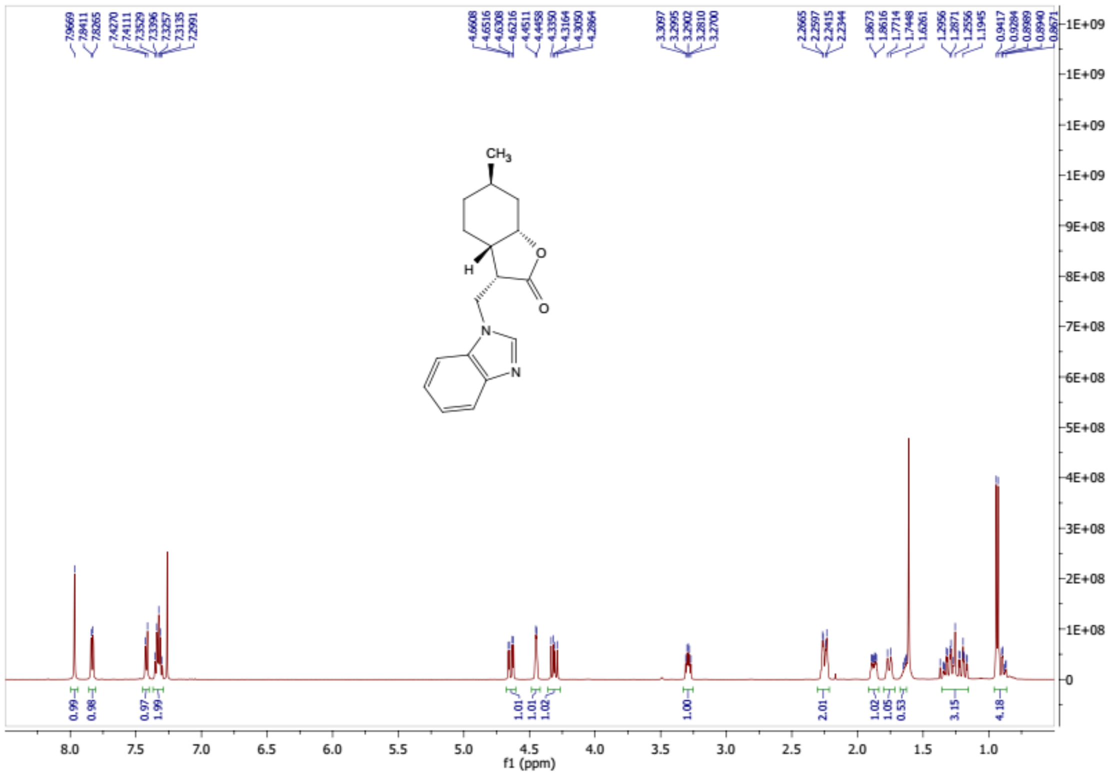

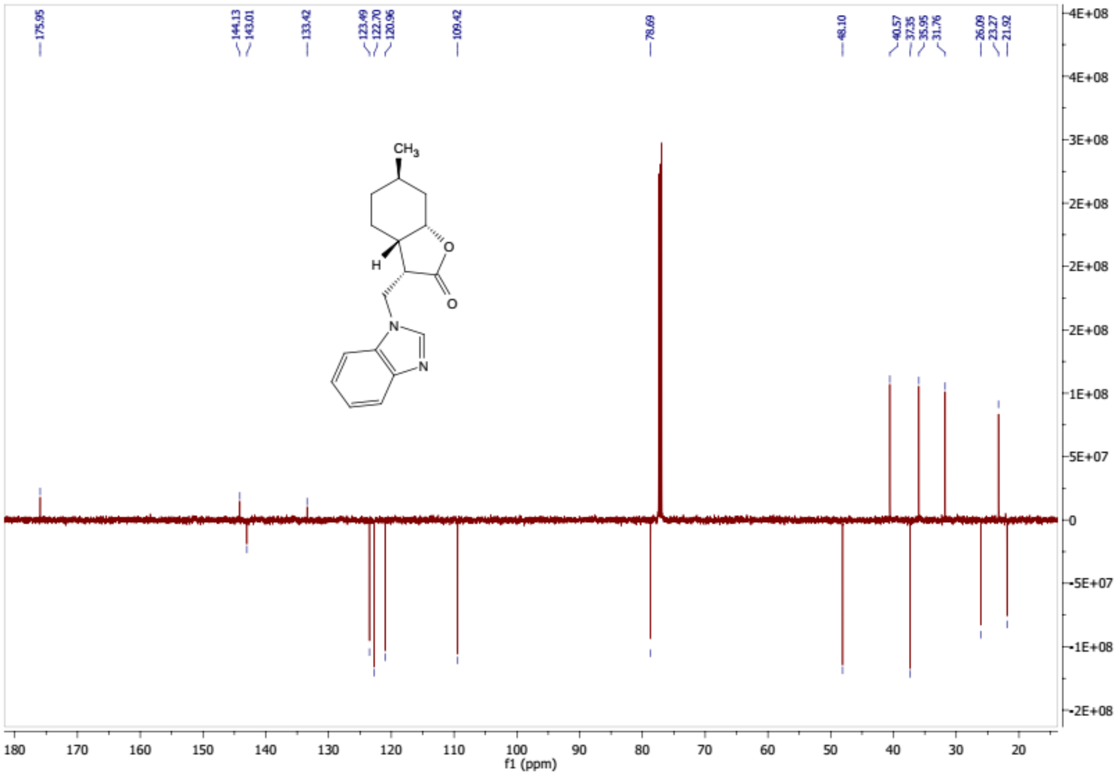

Figures S62-S63. ^{1}H (500 MHz, $CDCl_3$) and ^{13}C (125 MHz, $CDCl_3$) NMR spectra of **47a.**

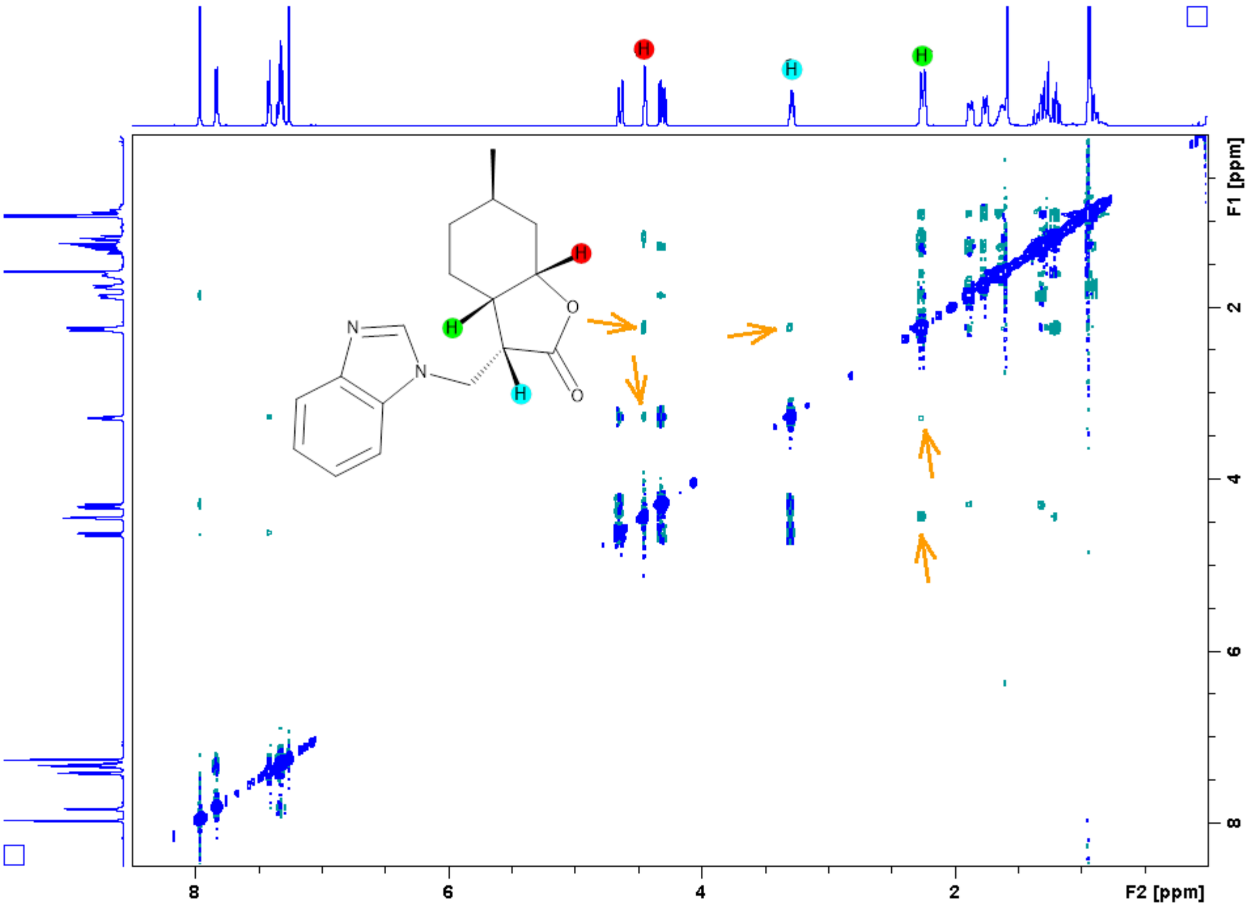

Figure S64. NOESY (500 MHz, $CDCl_3$) spectra of **47a.**

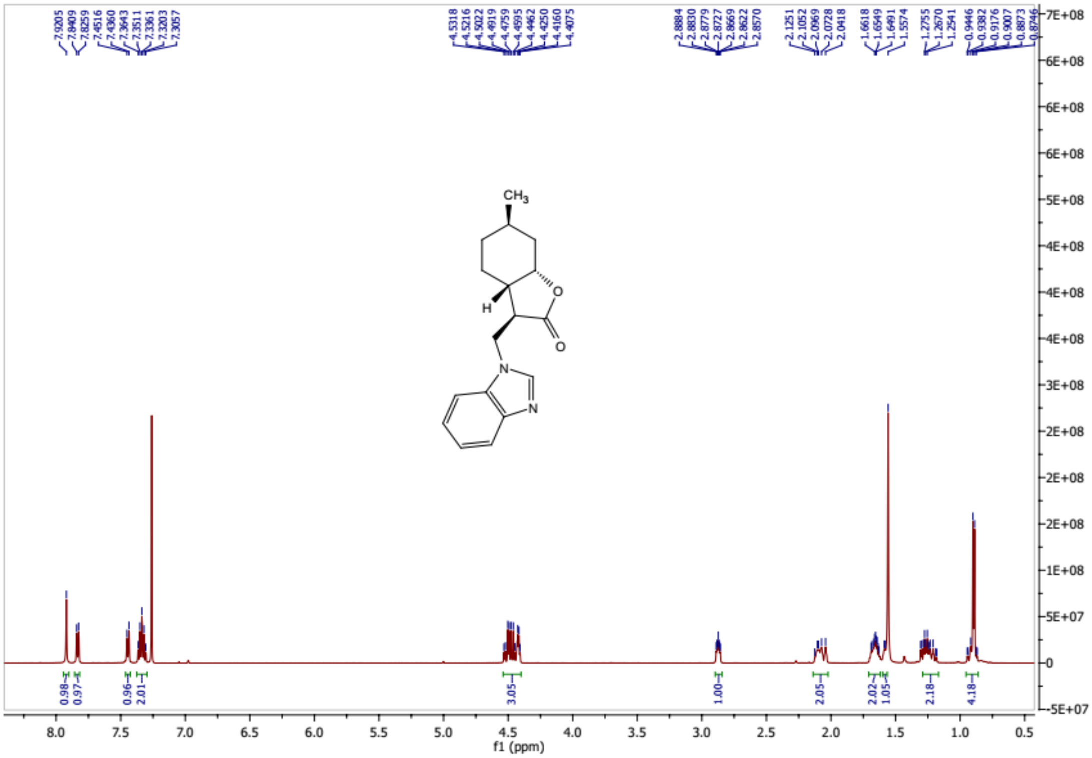

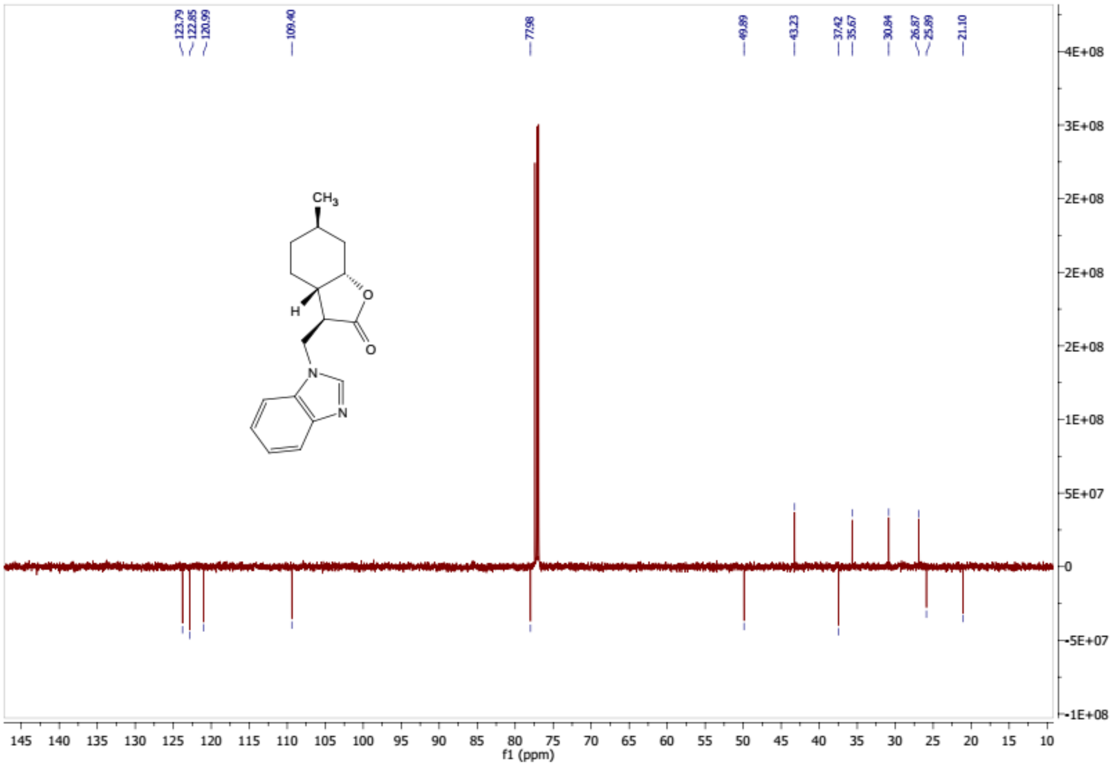

Figures S65-S66. ^{1}H (500 MHz, $CDCl_3$) and ^{13}C (125 MHz, $CDCl_3$) NMR spectra of **47b.**

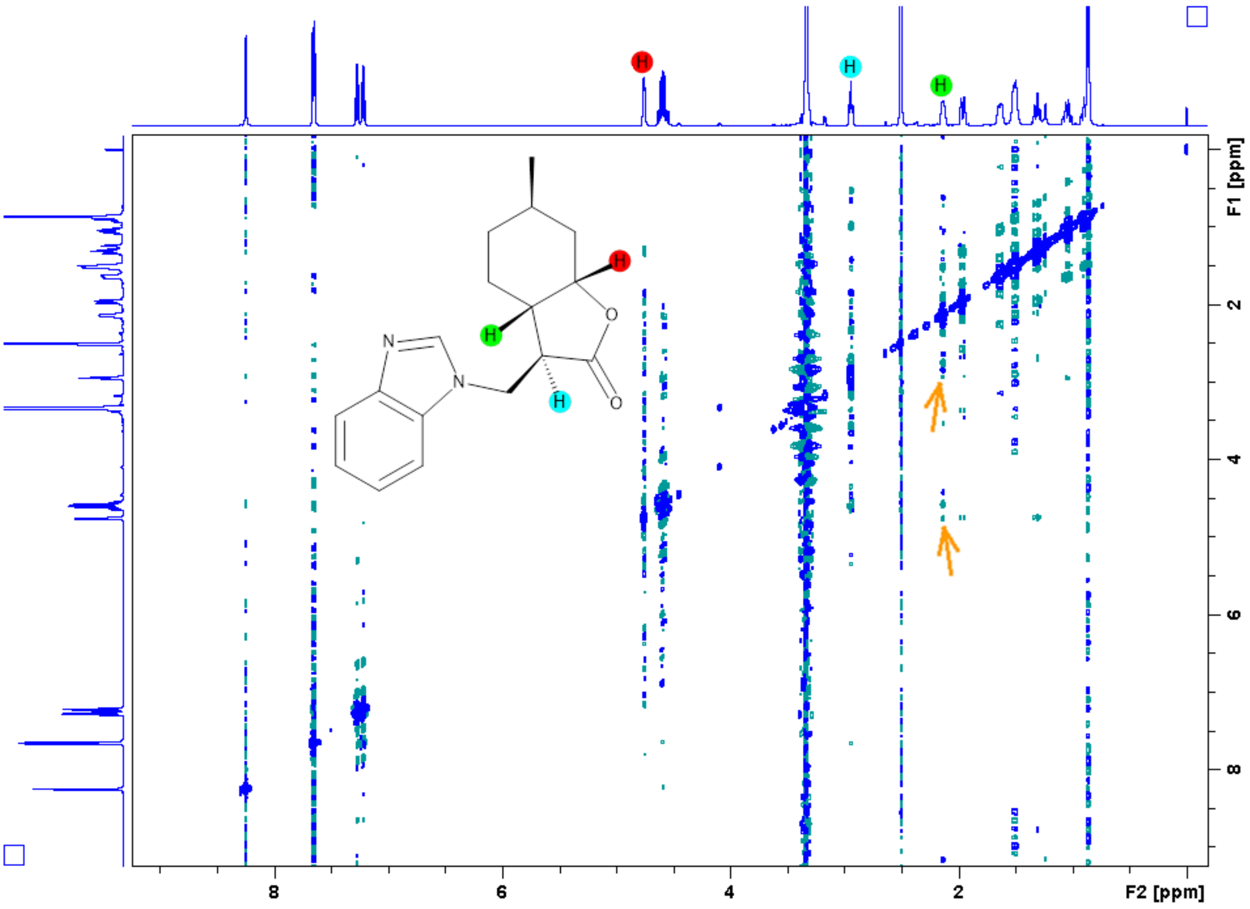

Figure S67. NOESY (500 MHz, DMSO-d_6) spectra of **47b.**

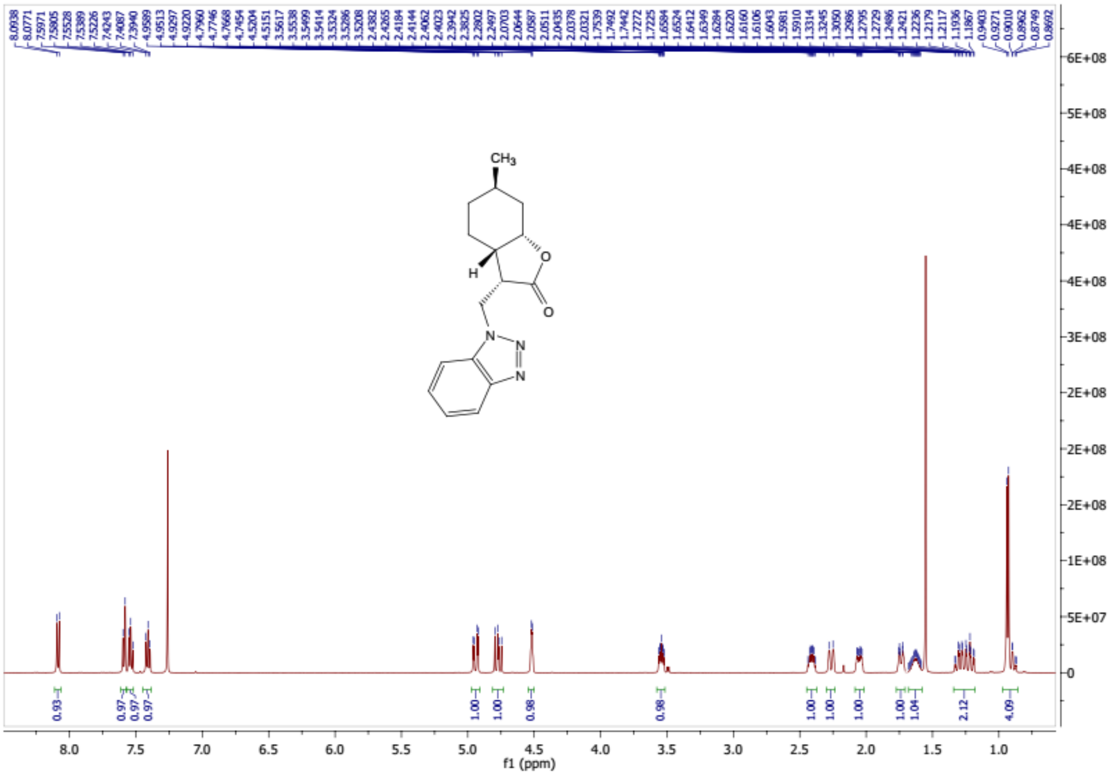

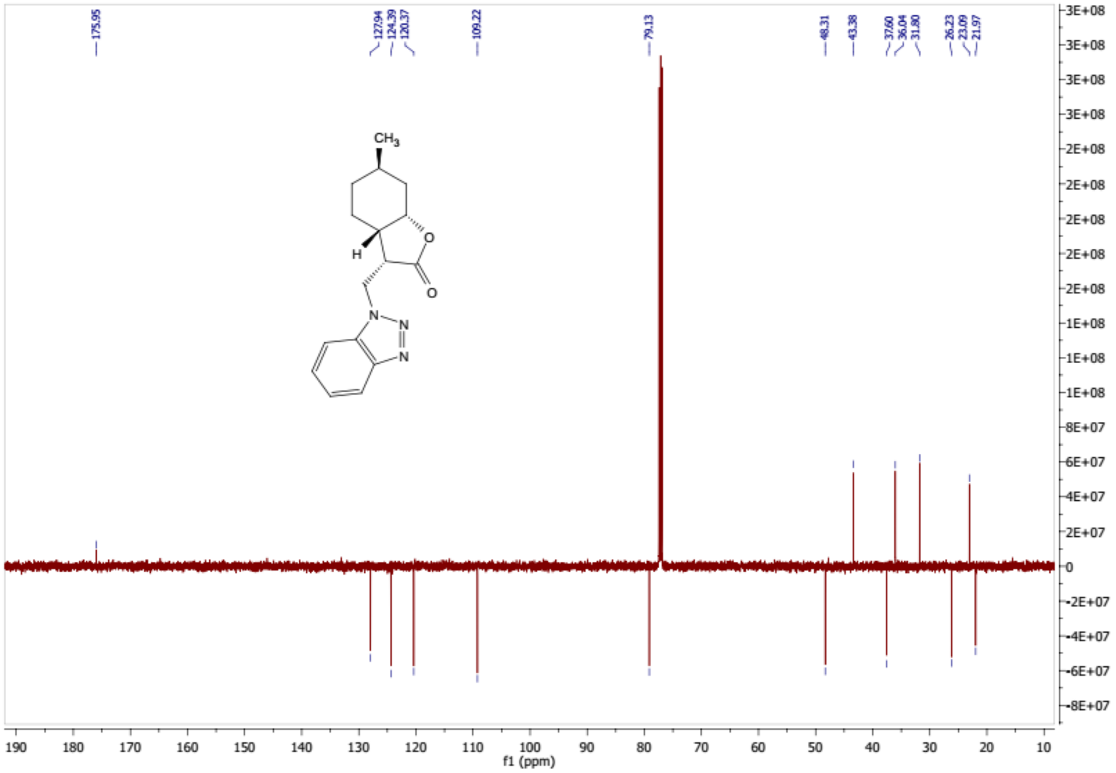

Figures S68-S69. ^{1}H (500 MHz, $CDCl_3$) and ^{13}C (125 MHz, $CDCl_3$) NMR spectra of **48a.**

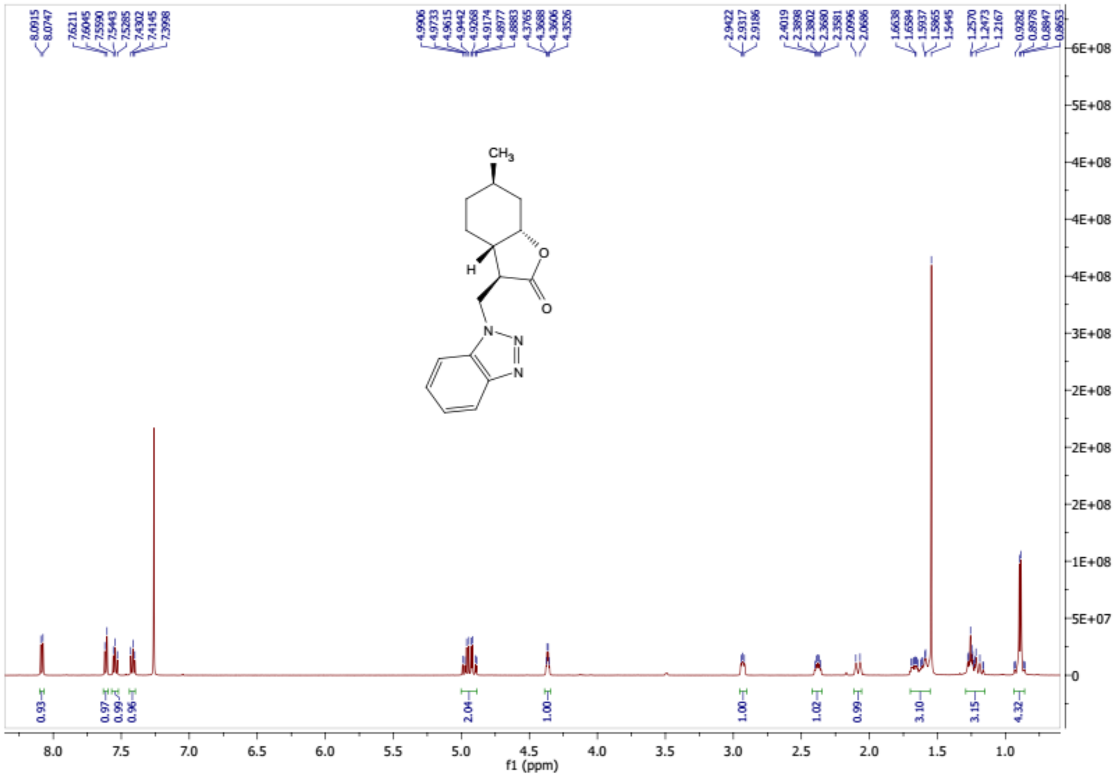

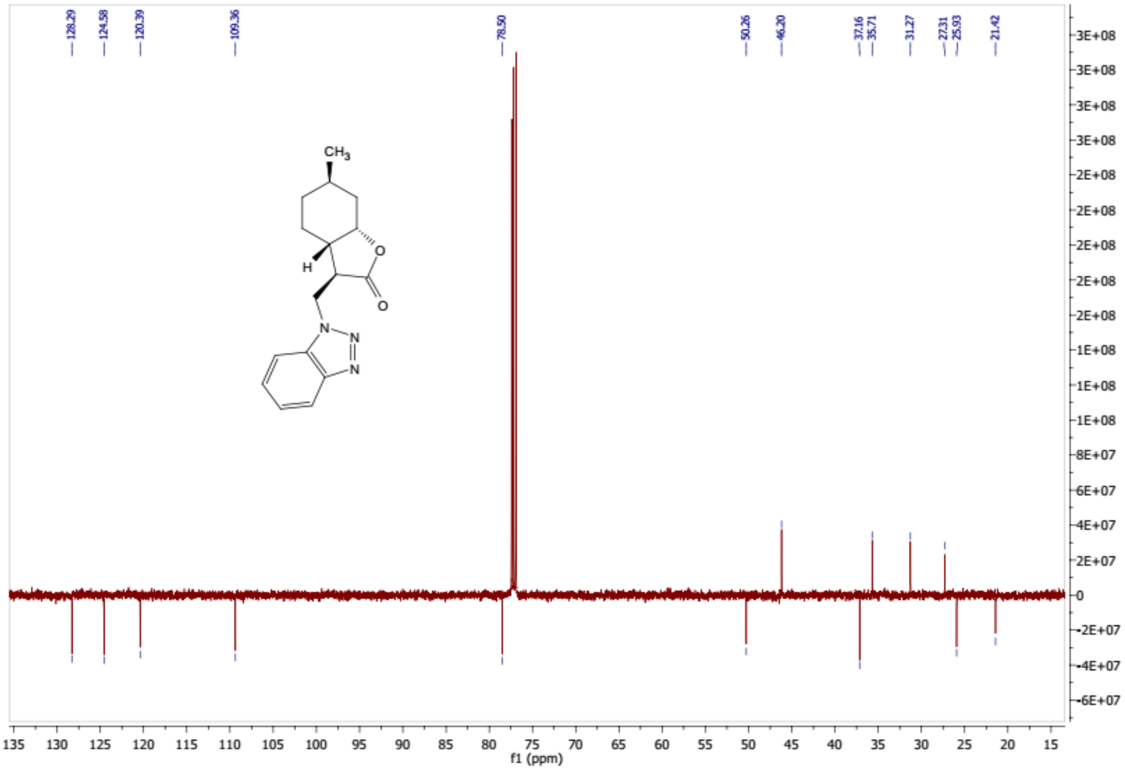

Figures S70-S71. ^{1}H (500 MHz, $CDCl_3$) and ^{13}C (125 MHz, $CDCl_3$) NMR spectra of **48b.**

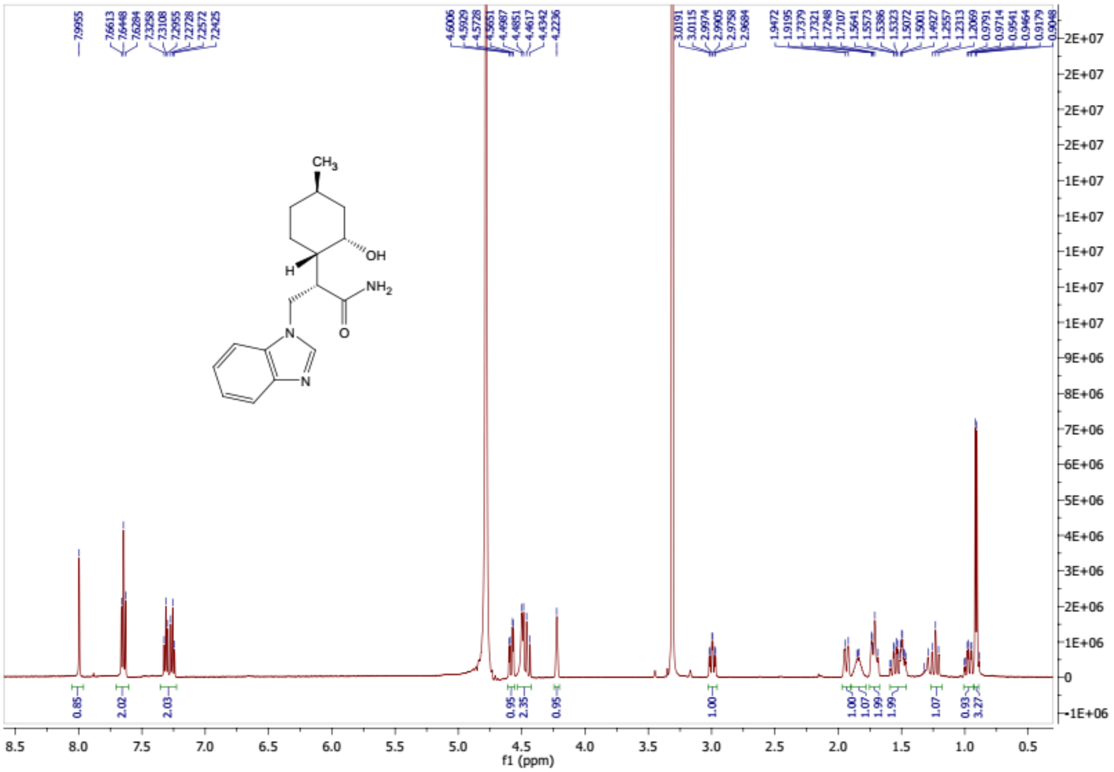

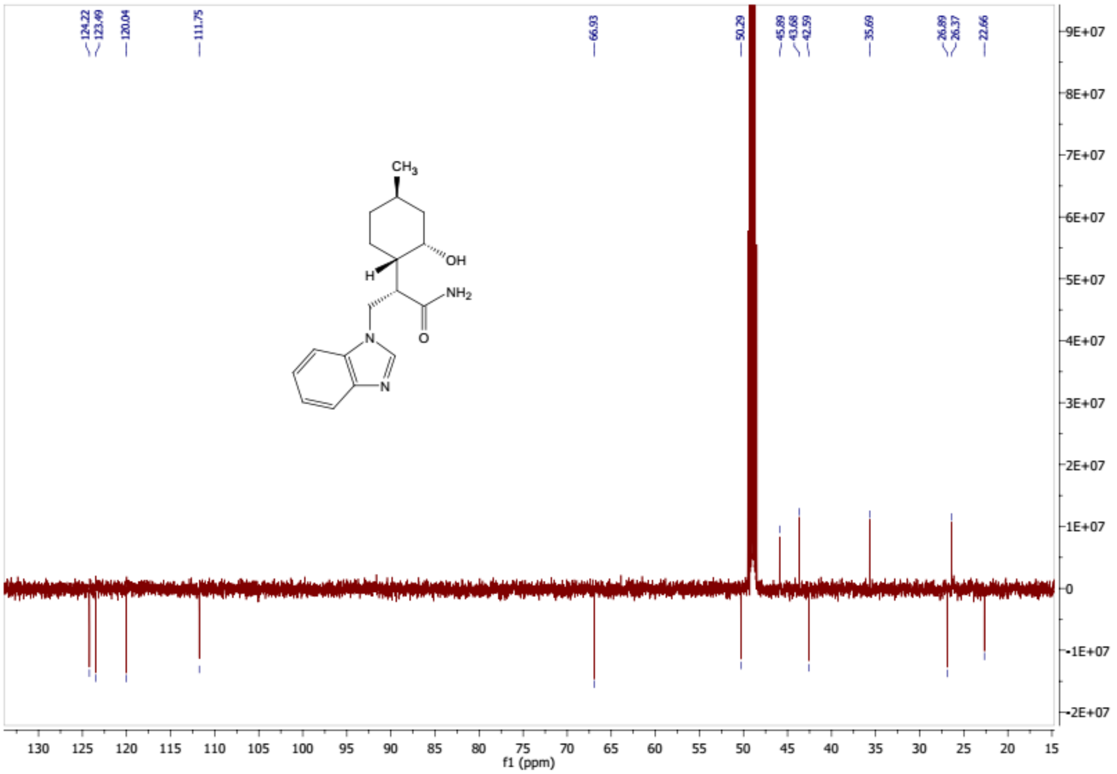

Figures S72-S73. ^{1}H (500 MHz, CD_3OD) and ^{13}C (125 MHz, CD_3OD) NMR spectra of **49a.**

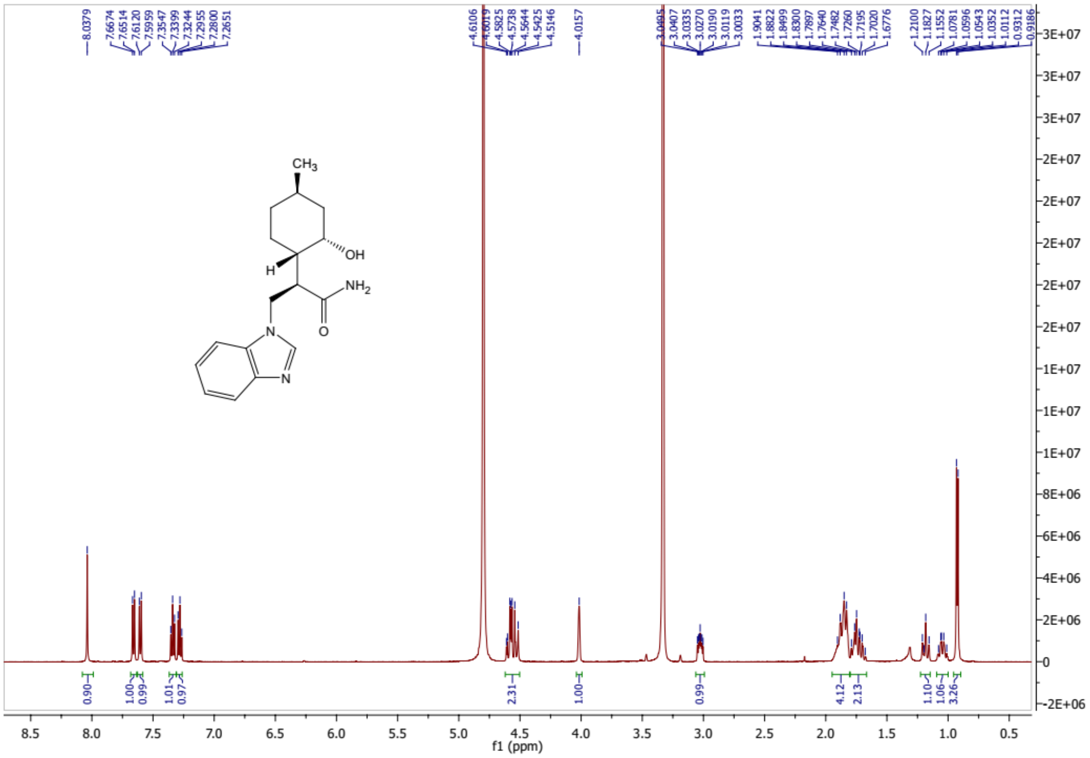

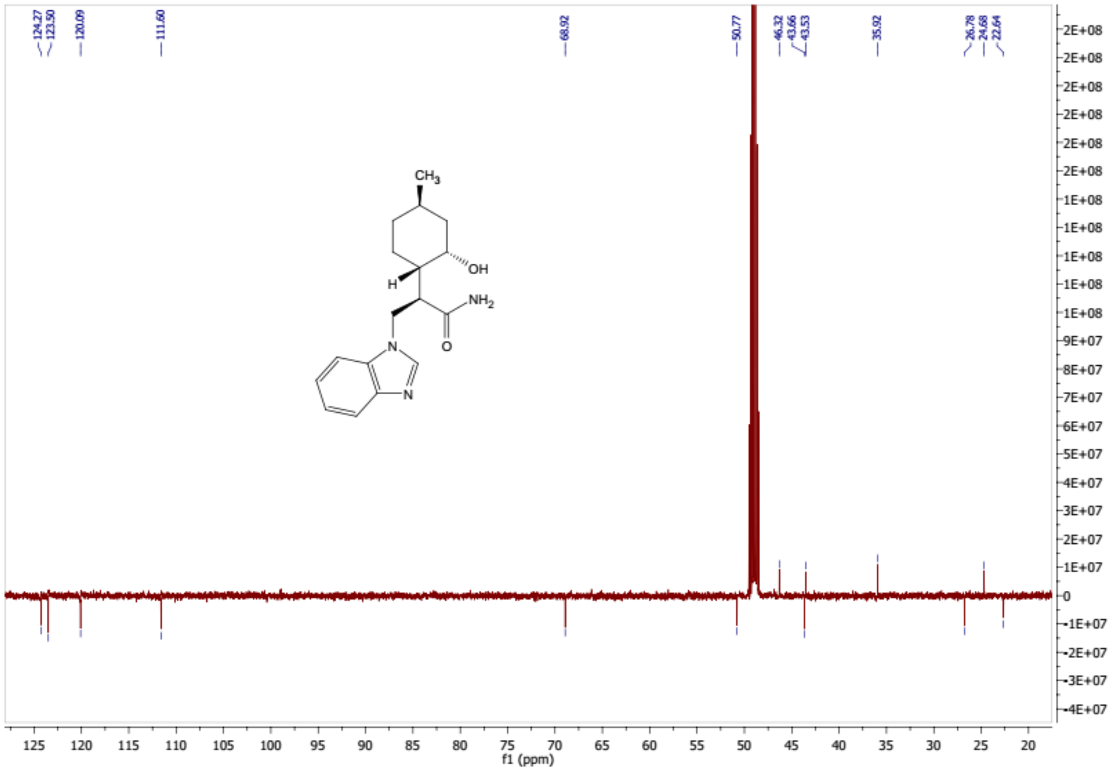

Figures S74-S75. ^{1}H (500 MHz, CD_3OD) and ^{13}C (125 MHz, CD_3OD) NMR spectra of **49b.**

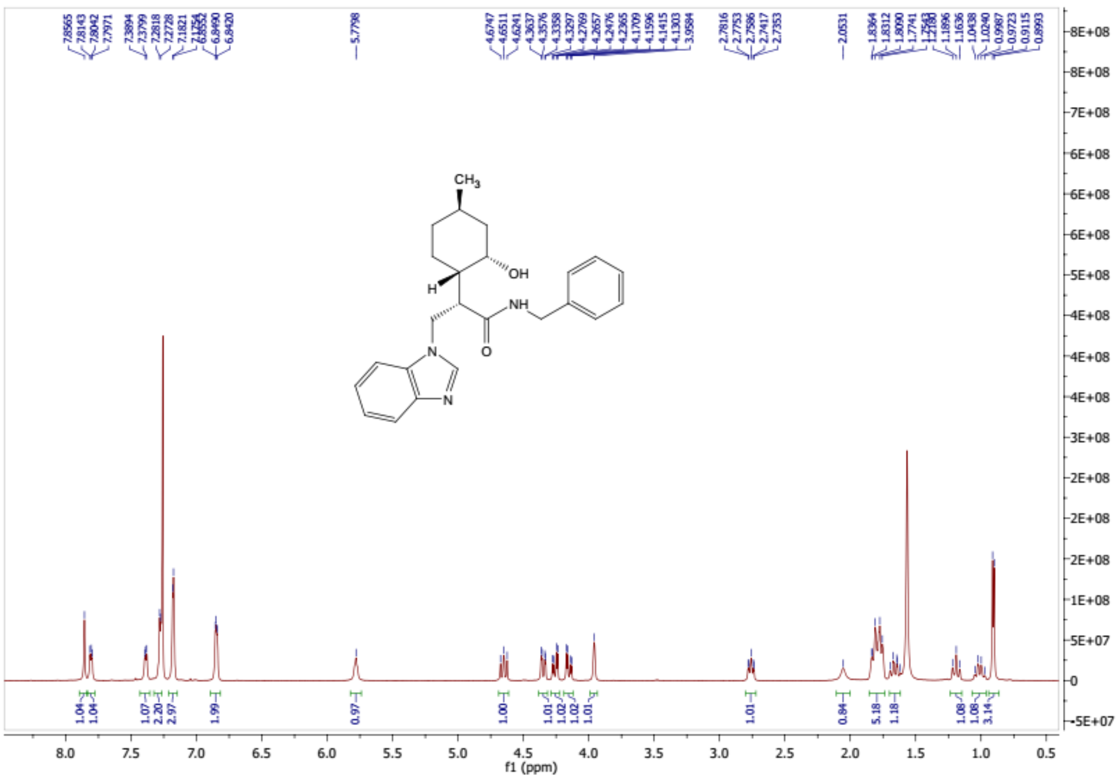

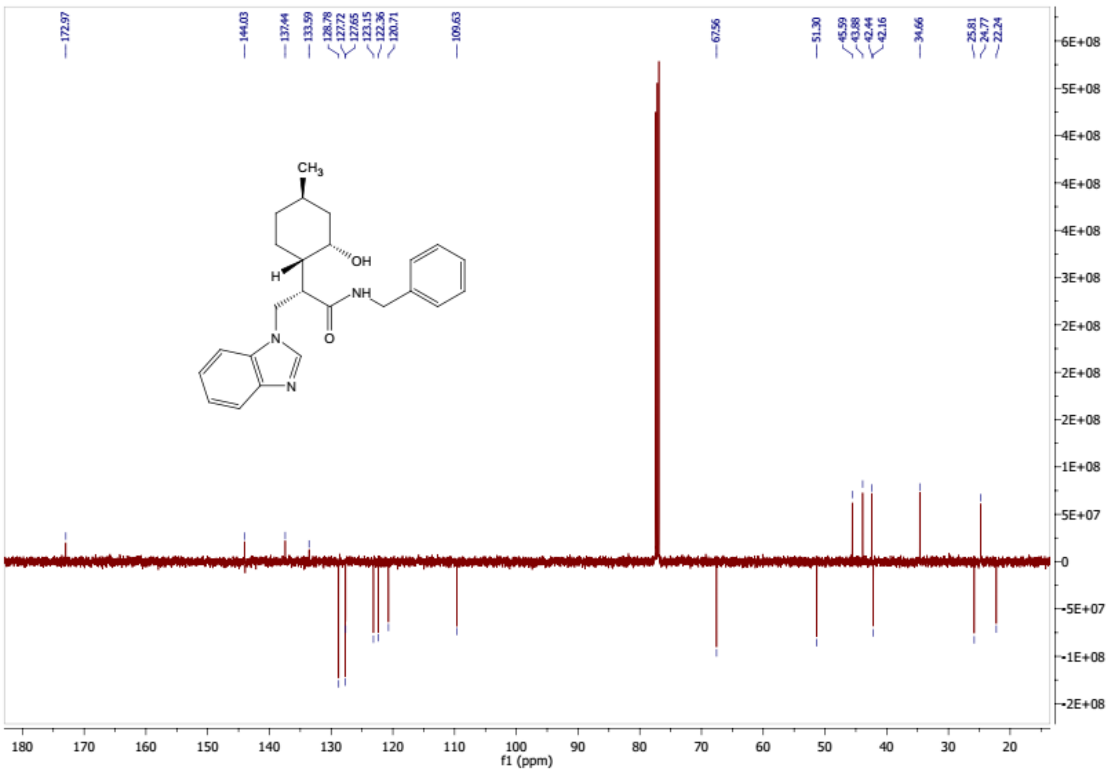

Figures S76-S77. ^{1}H (500 MHz, $CDCl_3$) and ^{13}C (125 MHz, $CDCl_3$) NMR spectra of **50a.**

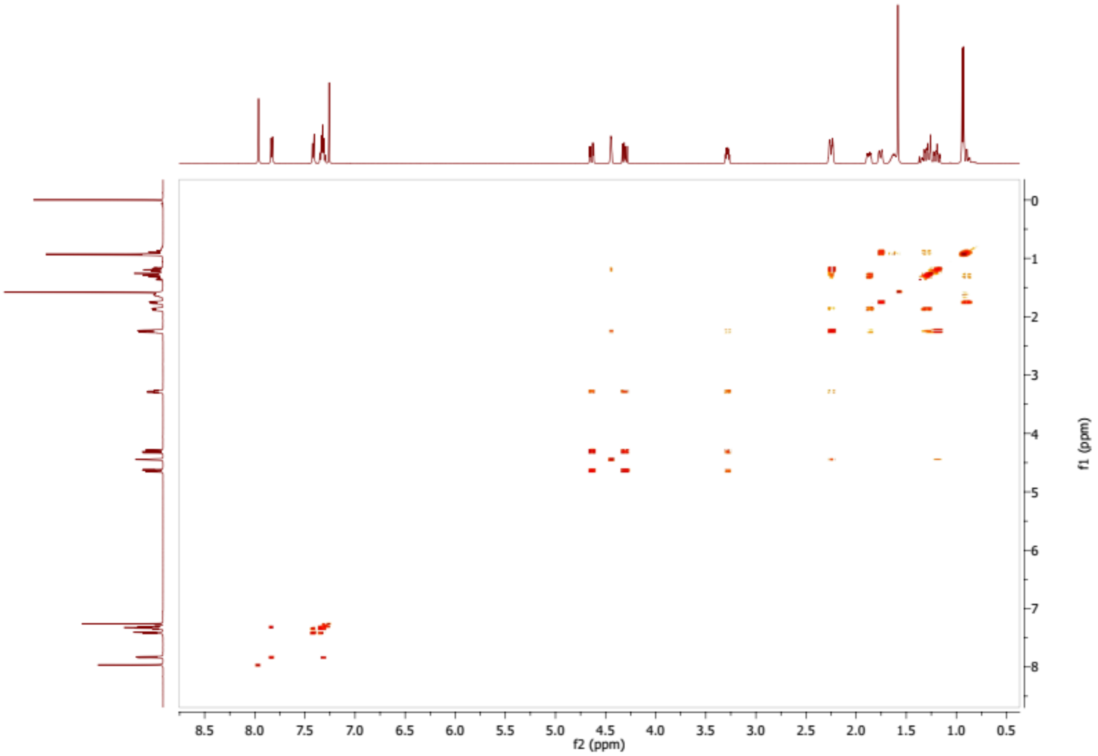

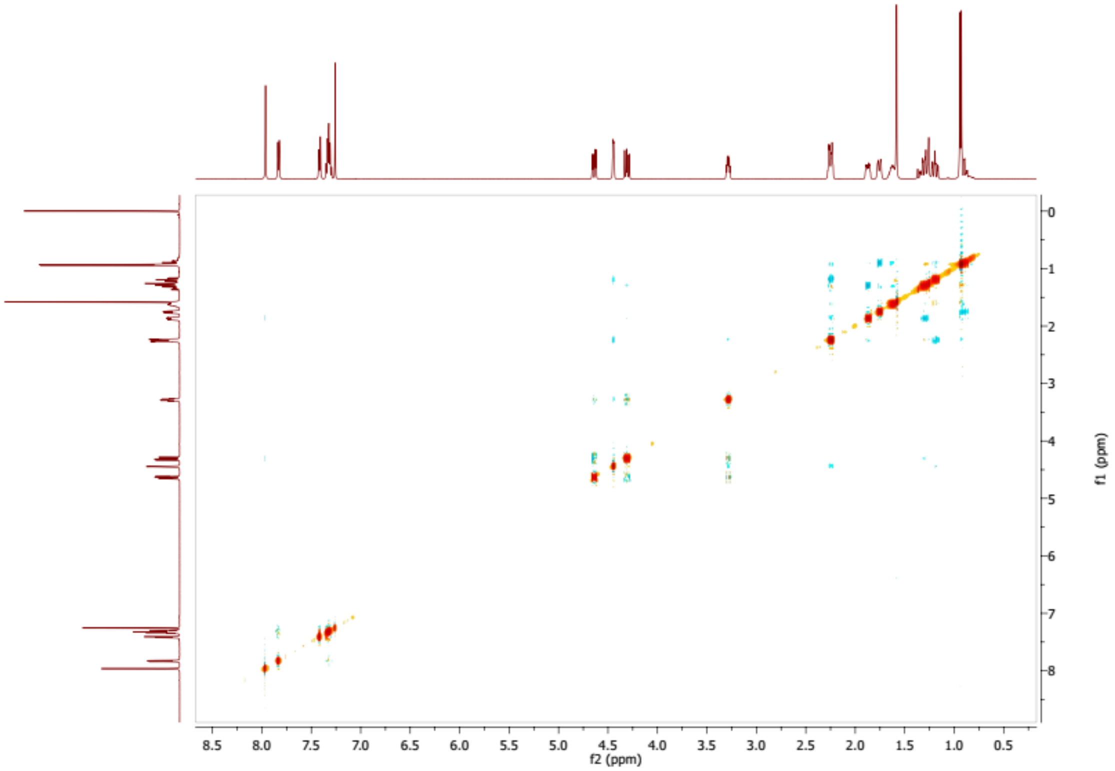

Figures S78-S79. COSY (500 MHz, $CDCl_3$) and NOESY (500 MHz, $CDCl_3$) NMR spectra of **50a.**

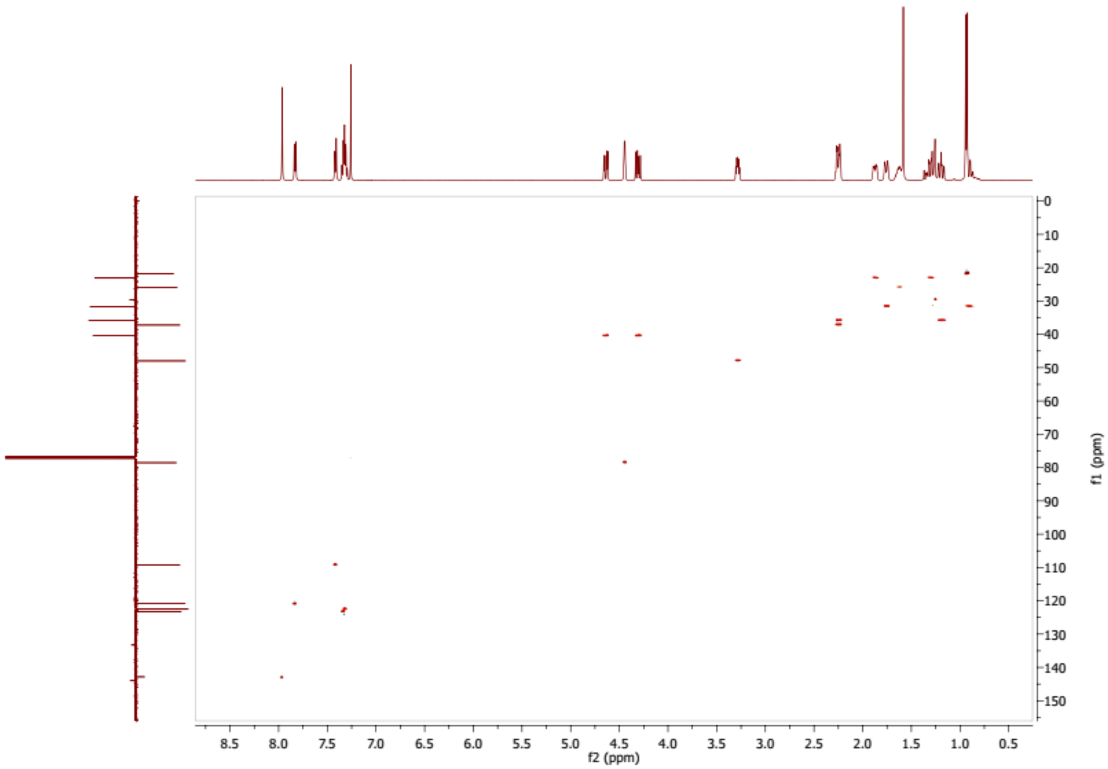

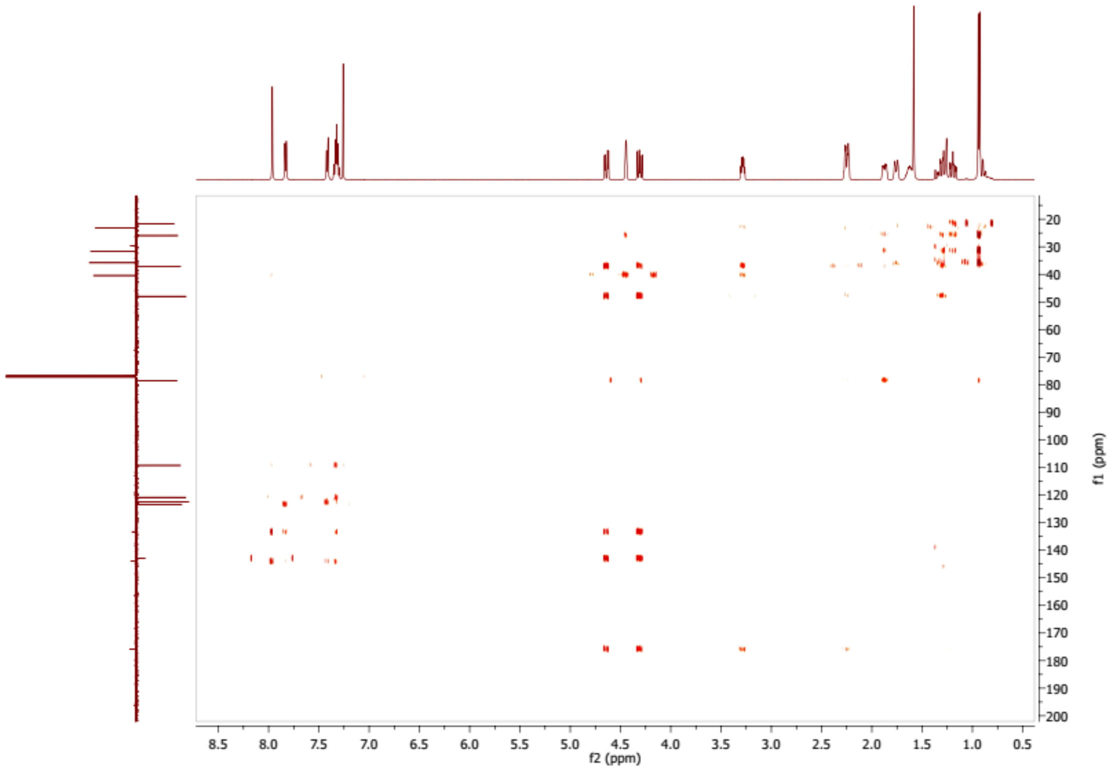

Figures S80-S81. HSQC (500 MHz, $CDCl_3$) and HMBC (500 MHz, $CDCl_3$) NMR spectra of **50a.**

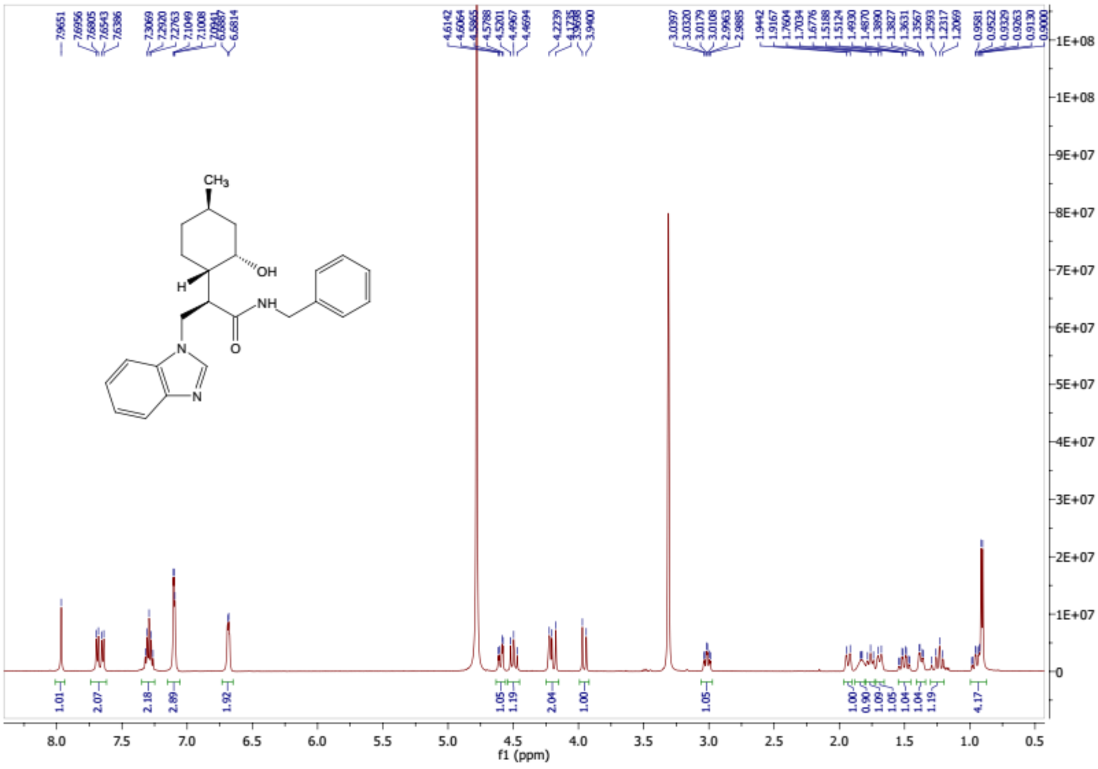

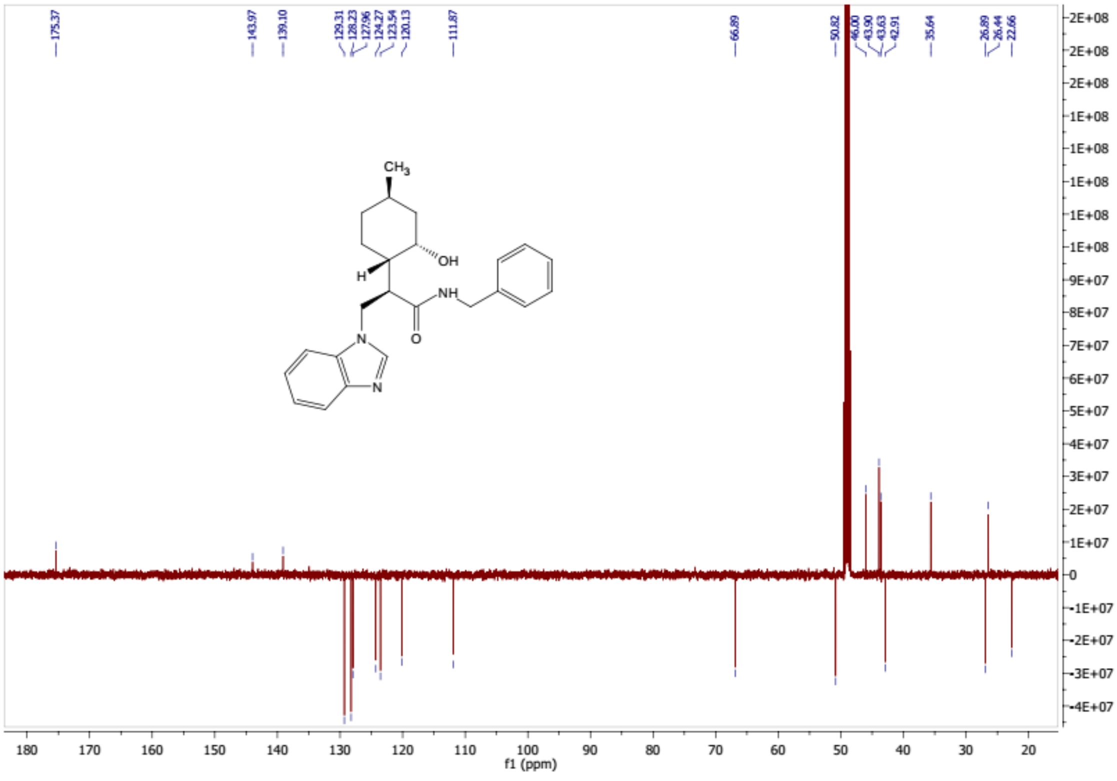

Figures S82-S83. ^{1}H (500 MHz, CD_3OD) and ^{13}C (125 MHz, CD_3OD) NMR spectra of **50b.**

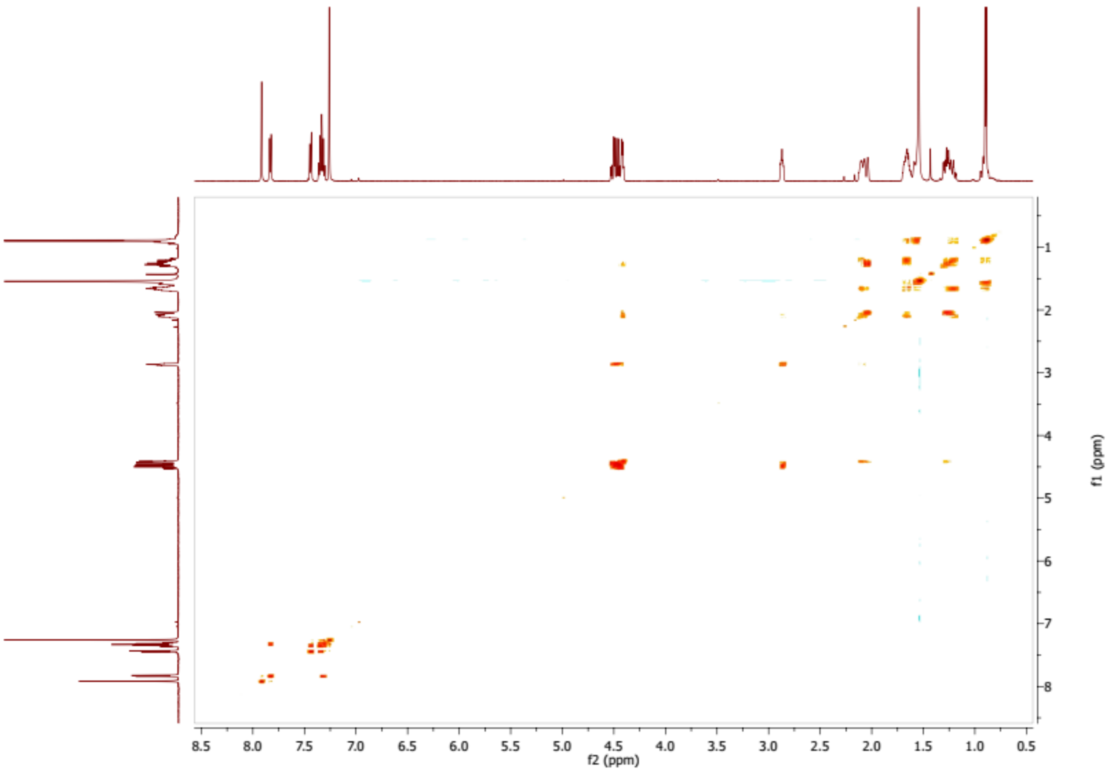

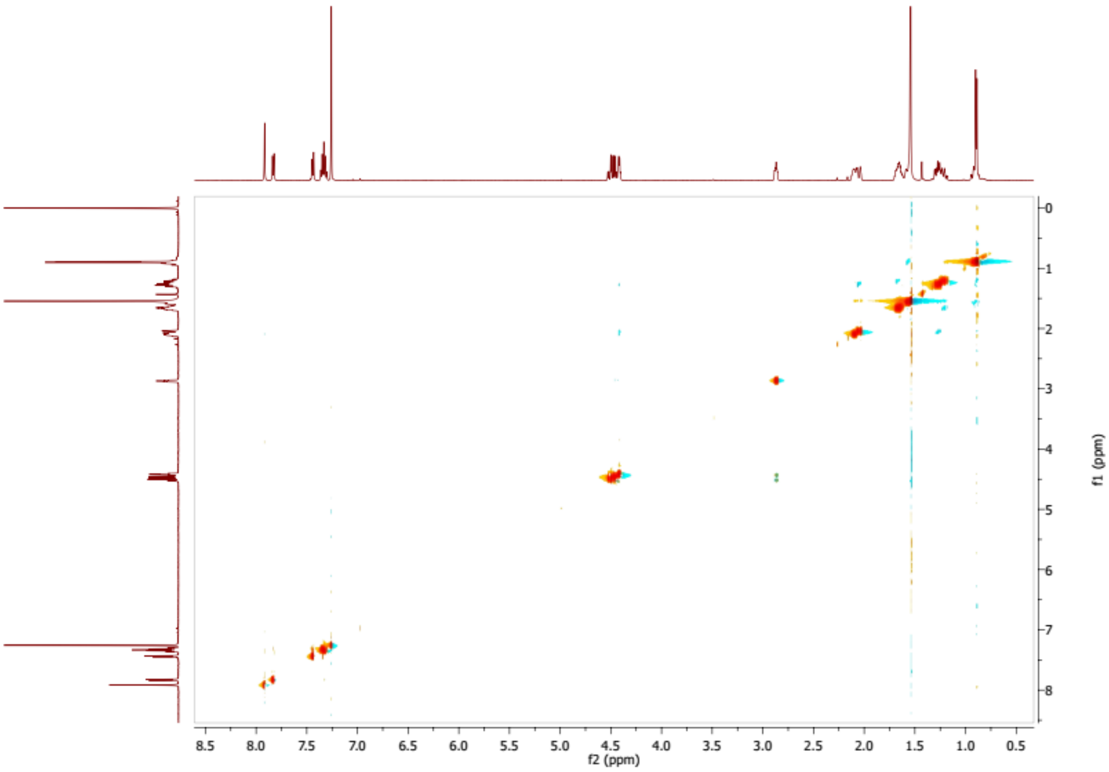

Figures S84-S85. COSY (500 MHz, $CDCl_3$) and NOESY (500 MHz, $CDCl_3$) NMR spectra of **50b.**

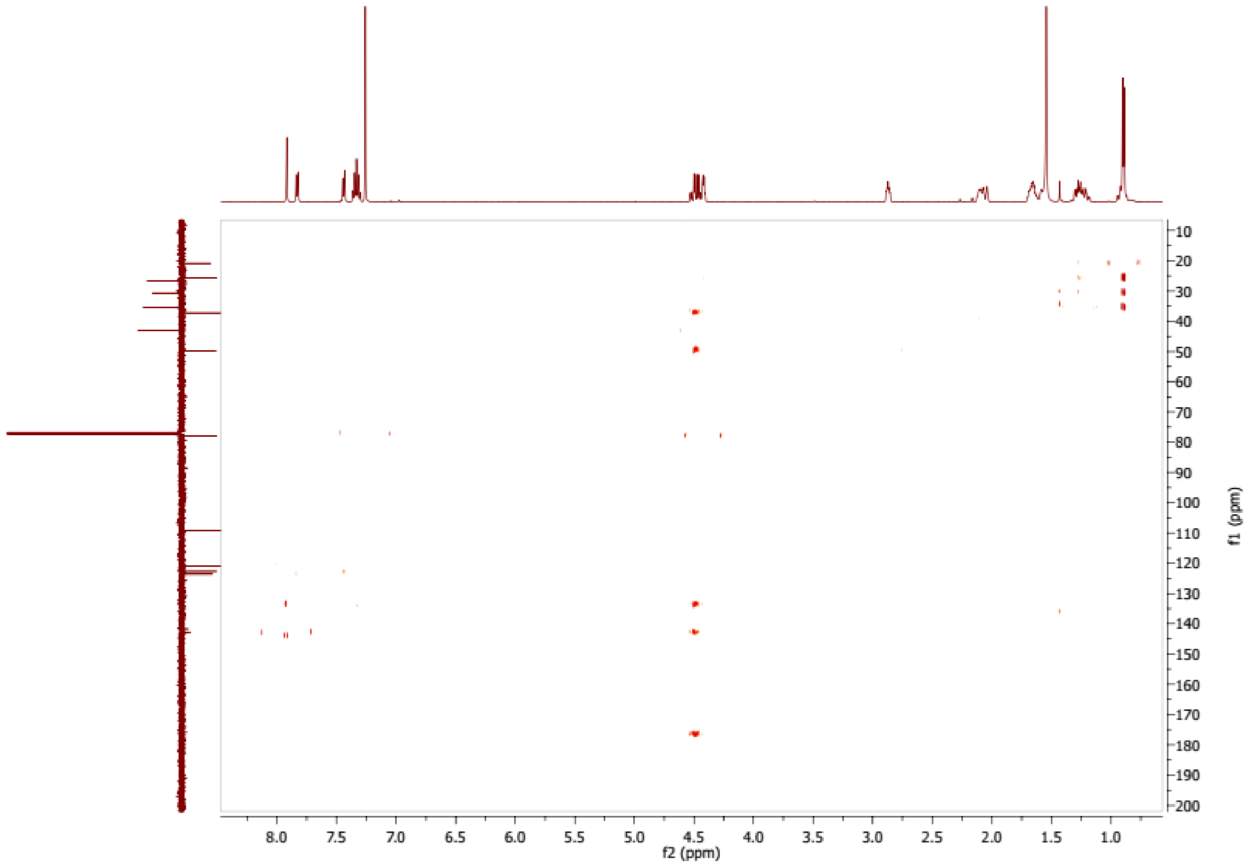

Figure S86. HMBC (500 MHz, $CDCl_3$) NMR spectra of **50b.**

2. Antimicrobial assay (results of all products)

Table S1. Biological screening data for every synthesized product (compounds **23**–**50**).

**Entry**	**Compound**	**MIC (μM) on the following bacteria:**		
		***S. aureus* ATCC 25923**	***S. aureus* MRSA ATCC 43300**	***E. coli* ATCC 25922**
1	**23**	**100**	>100	>100
2	**24**	**100**	>100	>100
3	**25**	**100**	>100	>100
4	**26**	**100**	>100	>100
5	**27**	**100**	>100	>100
6	**28**	**50**	>100	>100
7	**29**	**100**	>100	>100
8	**30**	**12.5**	>100	>100
9	**31**	>100	>100	>100
10	**32**	>100	>100	>100
11	**33**	>100	>100	>100
12	**34**	>100	>100	>100
13	**35**	>100	>100	>100
14	**36**	>100	>100	>100
15	**37**	>100	>100	>100
16	**38**	>100	>100	>100
17	**39**	>100	>100	>100
18	**40**	>100	>100	>100
19	**41**	>100	>100	>100
20	**42**	>100	>100	>100
21	**43**	>100	>100	>100
22	**44a**	>100	>100	>100
23	**44b**	>100	>100	>100
24	**45a**	>100	>100	>100
25	**45b**	>100	>100	>100

Table S1. Biological screening data for every synthesized product (compounds **23**–**50**). [continued]

Entry	Compound	MIC (μM) on the following bacteria:		
		S. aureus **ATCC 25923**	***S. aureus*** **MRSA ATCC 43300**	***E. coli*** **ATCC 25922**
26	**46a**	>100	>100	>100
27	**46b**	>100	>100	>100
28	**47a**	>100	>100	>100
29	**47b**	>100	>100	>100
30	**48a**	>100	>100	>100
31	**48b**	>100	>100	>100
32	**49a**	>100	>100	>100
33	**49b**	>100	>100	>100
34	**50a**	>100	>100	>100
35	**50b**	>100	>100	>100
36	**Ciprofloxacin**	4.2	4.2	2.1
37	**Tetracycline**	0.6	0.6	0.4

4. Molecular docking

Table S2. CDOCKER energy values for compounds **28** and **30.**

Compound	2W9S		4CJN		2ZCQ		3U2D	
	CDOCKER energy	NO. interactions	CDOCKER energy	NO. interactions	CDOCKER energy	NO. interactions	CDOCKER energy	NO. interactions
**28**	-8.9928	7	-8.72497	9	-33.7135	5	-23.3698	11
**30**	-1.28151	6	-3.79394	6	-24.8002	10	-15.5656	10

Table S3. In silico ADMET properties of compounds **28** and **30.**

Compound	Absorption level	Solubility level	BBB level	PPB level	CYP2D6 inhibition	Hepatotoxicity
**28**	0 (good)	3 (good)	2 (medium)	0 (<90%)	0 (non-inhibitor)	0 (nontoxic)
**30**	0 (good)	2 (low)	1 (high)	0 (<90%)	0 (non-inhibitor)	0 (nontoxic)

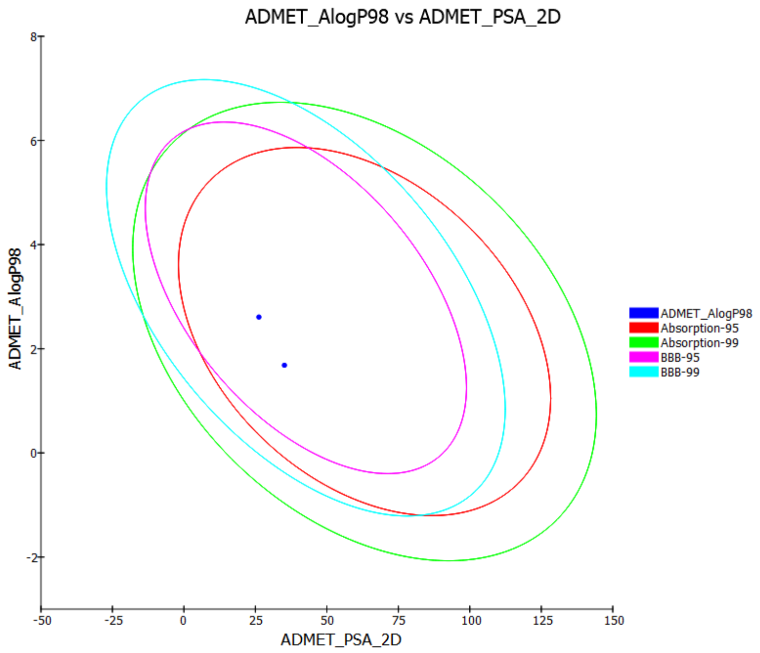

Figure S87. Plot of the Polar Surface Area (PSA) vs. LogP for a standard and test set showing the 95% and 99% confidence limit ellipses corresponding to the blood–brain barrier and intestinal absorption models of compounds **28** and **30.**
